# Influence of COVID-19 on trust in routine immunization, health information sources and pandemic preparedness in 23 countries in 2023

**DOI:** 10.1038/s41591-024-02939-2

**Published:** 2024-04-29

**Authors:** Jeffrey V. Lazarus, Trenton M. White, Katarzyna Wyka, Scott C. Ratzan, Kenneth Rabin, Heidi J. Larson, Federico Martinon-Torres, Ernest Kuchar, Salim S. Abdool Karim, Tamara Giles-Vernick, Selina Müller, Carolina Batista, Nellie Myburgh, Beate Kampmann, Ayman El-Mohandes

**Affiliations:** 1 Graduate School of Public Health and Health Policy, City University of New York, New York City, NY USA; 2 Barcelona Institute for Global Health, Barcelona, Spain; 3 Hospital Clínic, University of Barcelona, Barcelona, Spain; 4 London School of Hygiene and Tropical Medicine, London, UK; 5 Institute for Health Metrics and Evaluation, University of Washington, Seattle, WA USA; 6 University Clinic Hospital of Santiago de Compostela, Healthcare Research Institute of Santiago (IDIS), Santiago de Compostela, Spain; 7 Department of Pediatrics with Clinical Assessment Unit, Medical University of Warsaw, Warsaw, Poland; 8 Centre for the AIDS Program of Research in South Africa, Durban, South Africa; 9 Mailman School of Public Health, Columbia University, New York City, NY USA; 10 Anthropology and Ecology of Disease Emergence Unit, Institut Pasteur, Université Paris Cité, Paris, France; 11 Heidelberg Institute of Global Health, Heidelberg University Hospital, Heidelberg, Germany; 12 Baraka Impact Finance, Geneva, Switzerland; 13 Movement Health Foundation, Rio de Janeiro, Brazil; 14 Wits Health Consortium, Johannesburg, South Africa; 15 Charité Centre for Global Health, Berlin, Germany

**Keywords:** Public health, Communication

## Abstract

It is unclear how great a challenge pandemic and vaccine fatigue present to public health. We assessed perspectives on coronavirus disease 2019 (COVID-19) and routine immunization as well as trust in pandemic information sources and future pandemic preparedness in a survey of 23,000 adults in 23 countries in October 2023. The participants reported a lower intent to get a COVID-19 booster vaccine in 2023 (71.6%), compared with 2022 (87.9%). A total of 60.8% expressed being more willing to get vaccinated for diseases other than COVID-19 as a result of their experience during the pandemic, while 23.1% reported being less willing. Trust in 11 selected sources of vaccine information each averaged less than 7 on a 10-point scale with one’s own doctor or nurse and the World Health Organization, averaging a 6.9 and 6.5, respectively. Our findings emphasize that vaccine hesitancy and trust challenges remain for public health practitioners, underscoring the need for targeted, culturally sensitive health communication strategies.

## Abstract (web summary)

A survey of 23,000 adults in 23 countries in 2023 reports that the pandemic experience reduced participants’ willingness to be vaccinated for COVID-19 and receive routine vaccinations and reduced trust in recommendations from public health authorities.

## Main

The emergence of the severe acute respiratory syndrome coronavirus 2 virus in late 2019 precipitated a global health emergency that contributed to more than 7 million reported deaths globally as of 19 January 2024 (ref. ^1^) and an estimated 18.2 million excess deaths between 1 January 2020 and 31 December 2021 (ref. ^2^). The coronavirus disease 2019 (COVID-19) pandemic, requiring urgent international intervention, led to an accelerated pace of research and development of multiple safe, effective COVID-19 vaccines, which were first authorized for emergency use in December 2020^3^. The expeditious vaccine development and limited availability resulted in serious challenges in the equitable global distribution of vaccines, coupled with vaccine-related misinformation and mistrust of the science behind vaccine safety^4^.

Vaccine hesitancy^5^, pandemic fatigue^6^ and vaccine fatigue, defined as the ‘inertia or inaction toward vaccine information or instruction due to perceived burden and burnout’^7^, continue to present challenges to vaccine uptake in 2023. Although COVID-19 has been deprioritized as a substantial public health threat since 2023, the virus strains continue to circulate and, in some settings, lead to new increases in hospitalization and intensive care unit admission^1^. The potential impact of vaccine hesitancy on confidence in booster doses remains substantial^8^. In addition, documented spillover effects on routine immunization pose a threat for the reemergence of some childhood and adult vaccine-preventable diseases^9,10^.

In this Brief Communication, the fourth study in a series of annual global surveys across 23 countries (Brazil, Canada, China, Ecuador, France, Germany, Ghana, India, Italy, Kenya, Mexico, Nigeria, Peru, Poland, Russia, Singapore, South Africa, South Korea, Spain, Sweden, Türkiye, the United Kingdom and the United States)^11–13^, we report perspectives of adults in the general public on COVID-19 and routine immunization in late 2023, trust in pandemic information sources and collective preparedness to address any possible future pandemic. We also compare COVID-19 vaccine acceptance in 2023 to that in previous years to promote a better understanding of the current and future challenges public health authorities may face in encouraging vaccine uptake.

**Fig. 1:**
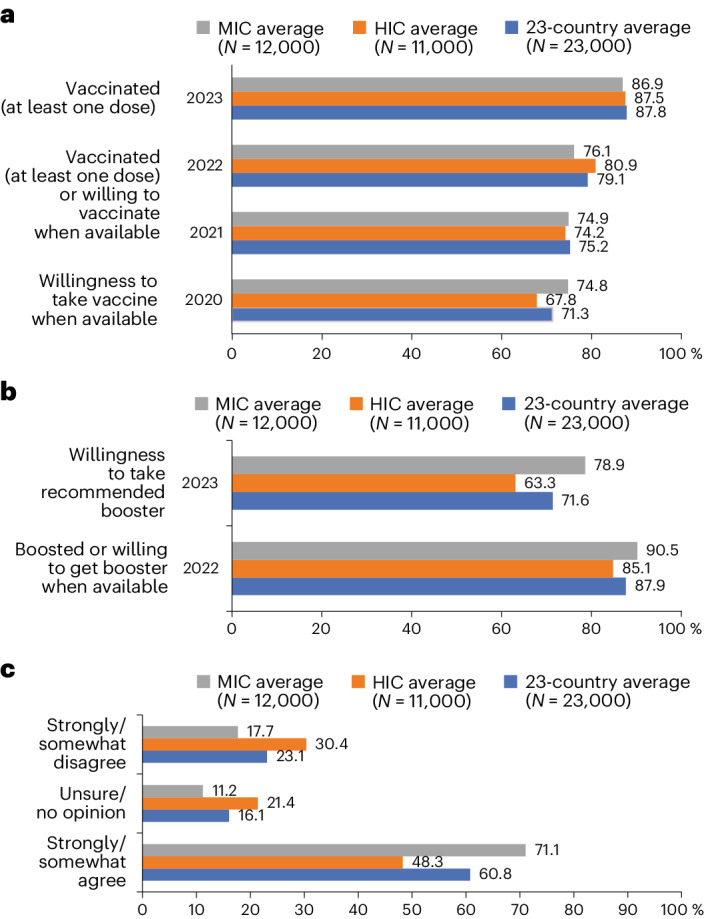
MICs: Brazil, China, Ecuador, Ghana, India, Kenya, Mexico, Nigeria, Peru, Russia, South Africa and Türkiye. **a**, COVID-19 vaccine acceptance among 23 countries, HICs and MICs. **b**, COVID-19 booster vaccine acceptance among 23 countries, HICs and MICs. **c**, Reported pandemic influence toward routine immunization. Four countries (Ghana, Kenya, Peru and Türkiye) were not included in the 2020 global survey. HICs: Canada, France, Germany, Italy, Poland, Singapore, South Korea, Spain, Sweden, the United Kingdom and the United States. ‘Routine immunization’ referrs to ‘other diseases (for example, flu, measles and viral hepatitis B)’ in the survey item.

**Extended Data Fig. 1:**
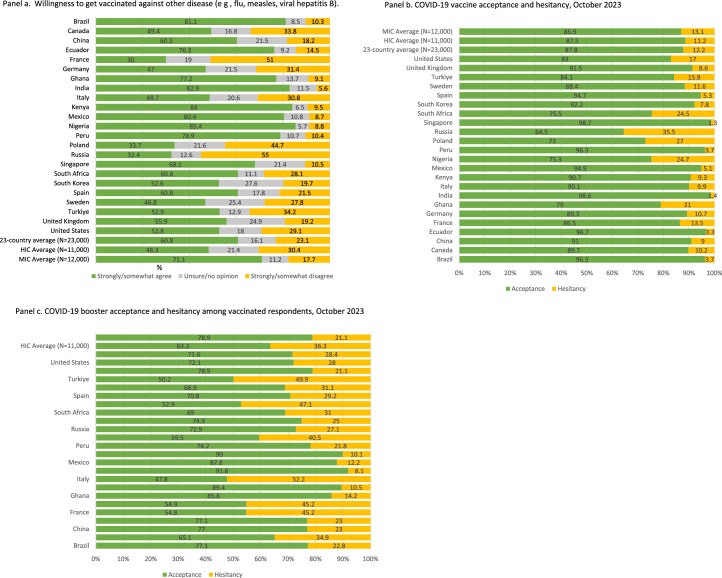
Reported willingness for routineimmunization, COVID-19 vaccine and booster acceptance and hesitancy in October 2023 by country. Sample size for each individual country n = 1,000. Middle-Income Countries (MIC): Brazil, China, Ecuador, Ghana, India, Kenya, Mexico, Nigeria, Peru, Russia, South Africa, Turkiye. High-Income Countries (HIC): Canada, France, Germany, Italy, Poland, Singapore, South Korea, Spain, Sweden, United Kingdom, United States. (**a**)“I am more willing to get vaccinated against other disease (e g, flu, measles,viral hepatitis B)”, (**b**) COVID-19 vaccine acceptance and hesitancy, October 2023, COVID-19 vaccine acceptance was defined as having received at least one dose of a COVID-19 vaccine. COVID-19 vaccine hesitancy was defined as not having received at least one dose of a COVID-19 vaccine. (**c**) COVID-19 booster acceptance and hesitancy among vaccinated respondents, October 2023. COVID-19 booster acceptance among vaccinated respondents was defined as willingness to take future recommended boosters (answer options “strongly agree” or “somewhat agree” to question “I will take the recommended COVID-19 booster”. COVID-19 booster hesitancy among vaccinated respondents was defined as having reportedeither “unsure/ no opinion” or “somewhat disagree” or “strongly disagree” to the same question.

The reported uptake of at least one COVID-19 vaccine dose rose to 87.8% in 2023 across the 23 countries (Fig. 1a), as compared with 36.9% in 2021 (*P* < 0.001) and 70.4% in 2022 (*P* = 0.002). The reported uptake of at least one COVID-19 vaccine was similar in middle-income countries (MICs; 86.9%) and high-income countries (HICs; 87.5%) (*P* = 0.381). COVID-19 vaccine booster acceptance among those vaccinated decreased from 87.9% in 2022 to 71.6% in 2023 (*P* < 0.001) (Fig. 1b). This decrease was most profound in HICs (from 85.1% to 63.3%, *P* < 0.001), compared with MICs (from 90.5% to 78.9%, *P* = 0.010). The perspectives on willingness to get vaccinated against diseases other than COVID-19 (for example, influenza, measles and hepatitis B) indicate that 60.8% of respondents may be more and 23.1% less willing to get vaccinated in 2023, following their experience during the COVID-19 pandemic (Fig. 1c). Individual country analyses on vaccine acceptance are available in Extended Data Fig. 1.

The COVID-19 pandemic led to widespread disruptions in routine immunization services globally, including for childhood doses, resulting in delayed and reduced vaccine uptake^10^. The results of this study demonstrate that 23.1% of respondents are less likely to accept vaccines for diseases other than COVID-19. Experience from the diversion of healthcare resources during the pandemic, along with lockdown measures and concerns about infection, highlights the need for resilient primary care systems, especially in maintaining access to crucial prevention interventions, such as routine childhood and adult vaccination. Other challenges, including disruptions to vaccine supply chains, underscore the importance of strengthening immunization systems and services to prevent future outbreaks^14,15^. Moreover, the extension of COVID-19 vaccine skepticism to other vaccines, including among parents who make vaccination decisions for their children^10^, signals a crucial need for ongoing efforts in vaccine education and trust building. Looking ahead, these insights should inform strategies to fortify healthcare systems against similar challenges to minimize disruptions and ensure continuity of essential health services, including routine vaccinations. Meanwhile, many communities are facing increased vulnerability to vaccine-preventable diseases^10^, highlighting the need for innovative strategies to ensure the continuity of routine immunization and COVID-19 vaccination campaigns to improve vaccine confidence.

**Extended Data Fig. 2:**
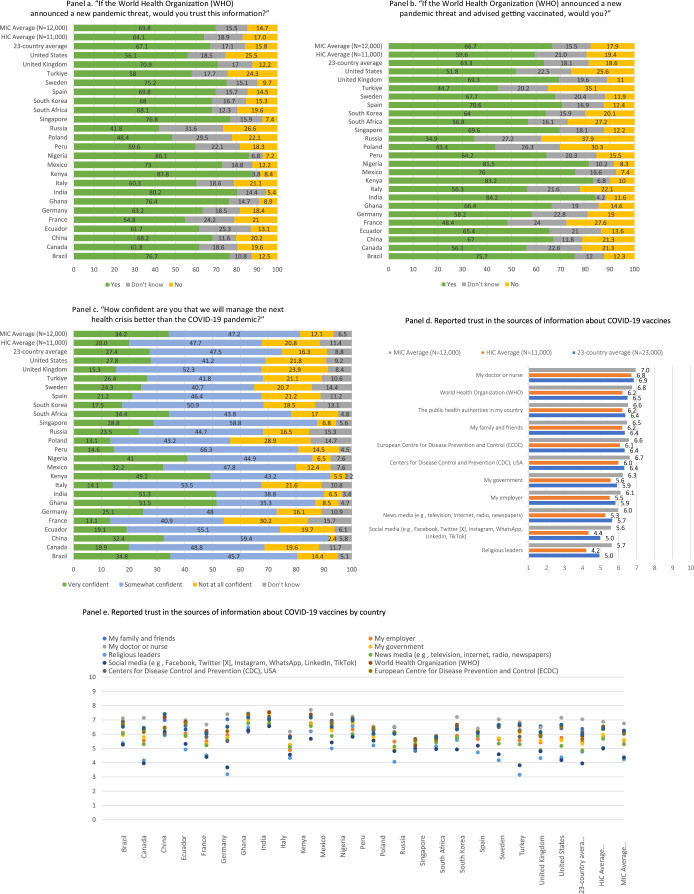
Reportedattitudes about future potential pandemic preparedness. Sample size for each individual country n = 1,000. Middle-Income Countries (MIC): Brazil, China, Ecuador, Ghana, India, Kenya, Mexico, Nigeria, Peru, Russia, South Africa, Turkiye. High-Income Countries (HIC): Canada, France, Germany, Italy, Poland, Singapore, South Korea, Spain, Sweden, United Kingdom, United States. (**a**) “If the World Health Organization (WHO) announced a new pandemic threat, would you trust thisinformation?”, (**b**) “If the World Health Organization (WHO) announced a newpandemic threat and advised getting vaccinated, would you?”, (**c**) “How confidentare you that we will manage the next health crisis better than the COVID-19 pandemic?”, (**d**) Reported trust in the sources of information about COVID-19 vaccines, (**e**) Reported trustin the sources of information about COVID-19 vaccines by country.

**Extended Data Fig. 3:**
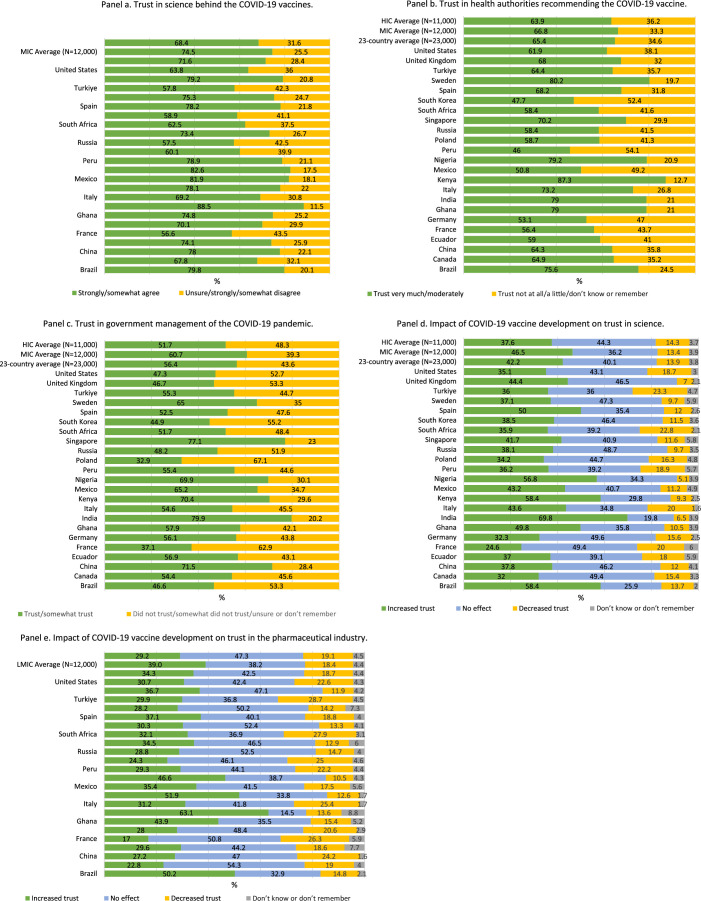
Trust in science, health authorities, government and pharmaceutical industry. Sample size for each individual country n = 1,000. Middle- Income Countries (MIC): Brazil, China, Ecuador, Ghana, India, Kenya, Mexico, Nigeria, Peru, Russia, South Africa, Turkiye. High-Income Countries (HIC): Canada, France, Germany, Italy, Poland, Singapore, South Korea, Spain, Sweden, United Kingdom, United States. Survey items: (**a**) ‘I trust the science behind the COVID-19 vaccines available to me,’ (**b**) ‘How much do you trust the health authorities that recommended you get a COVID-19 vaccine?,’ (**c**) ‘How much did you trust your government’s management of the COVID-19 pandemic in your country?,’ (**d**) ‘Did the development of COVID-19 vaccines affect your trust in science generally?,’ (**e**) ‘Did the development of the COVID-19 vaccines affect your trust in the pharmaceutical industry?’.

The survey responses on trust in sources that provide information or guidance on pandemic interventions revealed generally high levels of trust in those close to the individual, although all 11 studied sources averaged less than seven points on a ten-point scale. For example, ‘my doctor or nurse’ ranked highest at 6.9 and ‘my family and friends’ ranked at 6.4 (Extended Data Fig. 2d). Similarly, established health institutions such as the World Health Organization (WHO) (6.5) and the US Centers for Disease Control and Prevention (6.4) ranked high. Social media platforms (5.0) and religious leaders (5.0) each ranked neutrally (Extended Data Fig. 2d). There was variability across countries, for example, ‘religious leaders’ ranked 3.16 in Sweden and 3.19 in Germany but 6.57 in Nigeria and 6.72 in India, whereas ‘my doctor or nurse’ ranked 4.95 in Russia and 7.70 in Kenya (Extended Data Fig. 2e). Trust in health authorities that recommended COVID-19 vaccination was higher than trust in governments’ management of the COVID-19 pandemic at 65.4% and 56.4%, respectively (Extended Data Fig. 3). General trust in health authorities was 66.8% and 63.9% in MICs and HICs, respectively (*P* = 0.542), while general trust in government was 60.7% and 51.7% in MICs and HICs, respectively (*P* = 0.073). A decrease in perceived trust in science as a result of COVID-19 vaccine development was reported by 13.9% of respondents (MICs 13.4% and HICs 14.3%, *P* = 0.674). A decrease in perceived trust in the pharmaceutical industry as a result of COVID-19 vaccine development was reported by 18.7% of respondents (MICs 18.4% and HICs 19.1%, respectively, *P* = 0.772) (Extended Data Fig. 3). Trust in the science behind available COVID-19 vaccines was reported by 71.6% of respondents on average, with this value being 74.5% and 68.4% among MICs and HICs, respectively (*P* = 0.115) (Extended Data Fig. 3). The unprecedented speed of development, the novel application of mRNA technology and the proliferation of misinformation, particularly on social media, raised concerns among some about the thoroughness of testing and long-term safety of COVID-19 vaccines and contributed to increased skepticism regarding science generally, as well as its application to preventive and therapeutic applications in particular^16–18^. Moreover, factors such as prepandemic vaccine-related controversies and mistrust in pharmaceutical companies, governments and health institutions, sometimes the result of cultural beliefs or past negative experiences, have further complicated public health communication^16,19^.

**Fig. 2:**
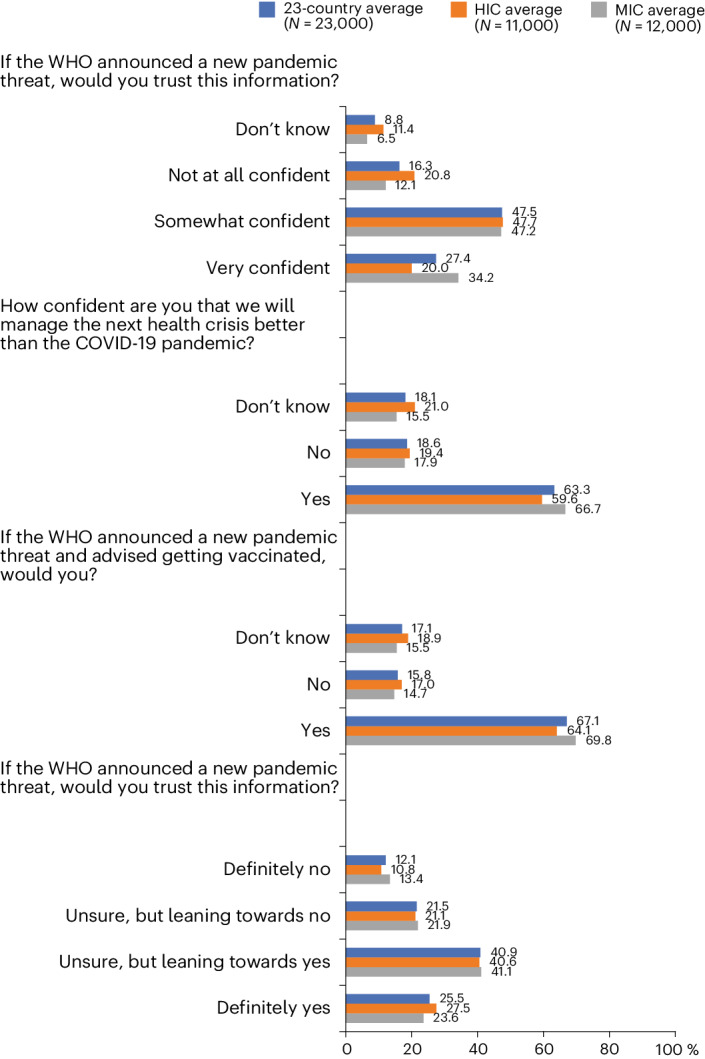
Reported trust in sources of COVID-19 information and reported COVID-19 treatment. MICs: Brazil, China, Ecuador, Ghana, India, Kenya, Mexico, Nigeria, Peru, Russia, South Africa and Türkiye. Four countries (Ghana, Kenya, Peru and Türkiye) were not included in the 2020 global survey. HICs: Canada, France, Germany, Italy, Poland, Singapore, South Korea, Spain, Sweden, the United Kingdom and the United States.

Perspectives on future pandemic preparedness reveal a mixed picture of confidence and trust among global populations. Approximately three-quarters (74.9%) of respondents are confident that society collectively will manage the next health crisis better than the COVID-19 pandemic, yet only 63.3% reported trusting a hypothetical WHO recommendation to vaccinate if such a crisis was announced (Fig. 2). Approximately a quarter of respondents in Russia (26.6%) and the United States (25.5%) express low trust in the WHO as a reliable source of information to announce a new pandemic threat (Extended Data Fig. 2a). Approximately half of respondents in Ghana (51.5%), India (51.3%) and Kenya (49.2%) report a high level of confidence in our collective ability to better manage the next potential health crisis (Extended Data Fig. 2c). A 2023 analysis in Kenya reporting 49.6% of respondents rating their own government’s management of the pandemic as very good or excellent may inform public confidence in future management capabilities^20^. Confidence in Ghana may be attributable to the government’s approach in preparing early readiness assessments, strategic and substantial investments in response planning and the effective use of surveillance technology^21^. India’s confidence in pandemic preparedness might be higher due to vaccine production capacity and public health investments in massive awareness campaigns and the rapid expansion of testing and contact tracing capabilities, despite having a large population and fragmented health system^22^. By contrast, 30.2% of respondents to our survey in France and 28.9% of respondents in Poland are ‘not at all confident’ in our collective ability, the highest percentages among the countries studied. These findings are comparable to panel data in France and Poland demonstrating low and decreasing trust in scientists among these populations during COVID-19^23^. Trust in the collective scientific and health communities to respond effectively to pandemic threats will require country-specific approaches that consider relevant sociocultural factors. How much individuals trust scientists and governments, respectively, has been observed as weakly related in Brazil and the United States, suggesting populations in these countries distinguish between these two health communicator groups, whereas the relationship was stronger in France, and populations view them as more closely aligned^23^. For example, in the United States and Brazil, a trend toward privatization and the erosion of the government’s role in mitigating public health threats exacerbated racial inequities and contributed to a fragmented response to the COVID-19 pandemic^24,25^. Ongoing global efforts to prepare for future global health threats promote a comprehensive ‘vaccines plus’ approach that incorporates social and behavioral preventive measures alongside rigorous testing and treatment^26^. Heightened vaccine hesitancy relative to COVID-19, pandemic fatigue and concerted disinformation campaigns have strong implications for plans to prevent or manage future pandemics, as well as a degree of spillover effect on our collective ability to control other vaccine-preventable diseases^27^. This may be particularly important as it pertains to routine childhood immunizations.

A vocal minority of vaccine-resistant populations continue to believe inaccurate and disproven claims, such as the effectiveness of ivermectin as a treatment for COVID-19 and some conspiracy theories, that drive resistance to vaccination^28,29^. Disinformation aiming to influence public opinion poses major challenges for communication campaigns that require heterogeneous data-driven precision public health approaches^30,31^. These strategies should focus on delivering clear, accurate and culturally sensitive information to specific communities through their preferred information channels and via trusted sources and on exposing the motivation of those behind disinformation. It is important to acknowledge that individuals often show a preference for information that aligns with their existing beliefs and perceive such information as more credible^32^. This biased selection and perception is more pronounced among those with higher health literacy^32^, which is a factor that health communication professionals must consider.

The critical need to catch up on routine immunizations and prepare for potential new pandemic threats, coupled with the continued spread of COVID-19, requires maintaining vigilance in addressing vaccine hesitancy globally. The varying degrees of hesitancy observed across different demographic groups and countries emphasize the importance of culturally and contextually relevant strategies that include the selection of welcomed credible sources as primary conduits of information to address and mitigate vaccine hesitancy. The findings of this study demonstrate that the WHO and the US Centers for Disease Control and Prevention, as well as the respondents’ personal doctor, were more highly trusted as sources of pandemic information. The communication of accurate and timely information, as well as countering misinformation, are pivotal in guiding public perception and behavior toward COVID-19 vaccination acceptance.

Furthermore, whole-of-society action has been recommended by pandemic researchers to address the thus far fragmented approaches seen in relation to pandemic preparedness and response^33,34^. Such an approach involves various sectors and actors in decision-making processes to build resilient systems and takes life risks other than health, such as employment, housing and food security status, into consideration. A proposed pandemic agreement is currently being debated in advance of the May 2024 World Health Assembly. It aims to strengthen global collaboration between countries and global health organizations, including the WHO, around improving One Health data monitoring and sharing, toward ensuring equitable access to preventive and therapeutic measures and strengthening health systems^35^. The intent of such an agreement would signal to Member States and their populations that pandemic preparedness to address the shortcomings of the COVID-19 pandemic response is being taken seriously, including the rapid, real-time country collaboration on surveillance and the equitable distribution of vaccines and other mitigation and elimination efforts.

Limitations to interpreting these data include the recognition of a fundamental discrepancy that may exist between the respondents’ reported willingness to receive the vaccine and their actual vaccination behavior. What people express in surveys can differ meaningfully from their actions^27^. Therefore, the findings regarding vaccine acceptance and hesitancy should not be directly equated with actual vaccine uptake; rather, the reported responses reflect attitudes and opinions at a specific point in time. As public perceptions of the COVID-19 pandemic and vaccination evolves, so too might their willingness to be vaccinated. This temporal aspect suggests that the acceptance levels reported in our study are subject to change due to a variety of factors, including new information about the virus and the vaccine, changes in public health recommendations and shifts in societal norms and attitudes toward vaccination. While our study assessed individuals’ perceptions of trust in sources of pandemic information, including governments and health authorities, we did not investigate the quality of country responses to the pandemic, which may be an important determinant of such trust, given its independent association with COVID-19 vaccination^20^. Our study’s design did not allow for a detailed analysis of the nuanced relationship between language, trust and cultural context, while early research on the impact of health communication language on vaccine hesitancy in bilingual settings may be mediated by cultural factors regarding trust in health and governing institutions^36^. We permitted participants to respond using their preferred language within their country.

This study reveals that a substantial proportion of individuals express resistance to vaccination and that concerns about COVID-19 vaccination appear to have spilled over to affect other vaccine-preventable diseases. This underscores the increasingly urgent necessity for sustained vaccine education and trust-building efforts. Moreover, although we found that people were generally confident that society will handle future health crises better, there remains a notable lack of trust and potential adherence to the recommendations of public health authorities. Health system preparedness for future outbreaks and global health threats should include improving vaccine accessibility and vaccine demand through effective, culturally and contextually relevant public communication strategies and innovative use of digital and social media in health education employing infodemic countermeasures.

## Methods

### Study design and sample

This study employed random stratified sampling in a 23-panel cross-sectional design (Extended Data Table 1 and Reporting Summary). A target quota was established for four strata (that is, age, gender, country-specific statistical regions and country-specific levels of education) according to the latest available country data for these strata and with a minimum quota of 50 participants per strata^37–41^. There were 23,000 participants, 1,000 from each country, the populations for which collectively represent nearly 60% of the world’s population^42^. MICs consisted of Brazil, China, Ecuador, Ghana, India, Kenya, Mexico, Nigeria, Peru, Russia, South Africa and Türkiye and HICs consisted of Canada, France, Germany, Italy, Poland, Singapore, South Korea, Spain, Sweden, the United Kingdom and the United States^43^. The details on participant recruitment are described in Reporting Summary.

### Survey instrument

The instrument (Supplementary Information) included 30 items from previous study iterations, 9 new items on misinformation and pandemic preparedness and 11 items on trusted sources of information selected by the authors following a scoping review of peer-reviewed primary research that used survey methodologies to assess these topics^44–57^. The selected items aimed to cover a broad spectrum of information channels that people might rely on for pandemic-related information. They include formal and informal sources, spanning from international health organizations to personal acquaintances, attempting to capture a comprehensive view of trust in different information environments and applicable for a global sample. The questionnaire was cross-culturally translated from English to the two most widely spoken lanugages in each country.

### Statistical analysis

Descriptive statistics were used to report COVID-19 vaccine uptake and booster acceptance. In 2022, COVID-19 booster acceptance was defined as having received at least one dose of a booster and if not, willingness to take the booster when it is available (answer options ‘strongly agree’ or ‘somewhat agree’ to question ‘I will take the COVID-19 booster dose(s) when it is available to me’). We also report the descriptive statistics for items related to reported attitudes toward routine immunization, trusted sources of information and future pandemic preparedness. The participants ranked the trustworthiness of these sources on a scale of 1 to 10, where 1 indicated ‘no trust at all’ and 10 represented ‘complete trust’. For each source of information, individual scores from participants within a country were aggregated to produce a single mean score for that source in that country. This method allowed for a concise representation of the collective trust level in each information source per country. The country-specific weighted estimates were used to compute 23-country average as well as averages for MIC and HIC country groupings. Independent sample *t*-tests were used to compare average estimates over time as well as for HIC and MIC country groups. All the analyses were conducted in SAS version 9.4 software. All the estimates reported in the paper have a maximum credibility interval of error of ±3.1 percentage points. The country-specific standard errors for each estimate are provided in Extended Data Table 2.

### Ethics and inclusion statement

This study was approved and the survey administered by Emerson College, Boston, USA under institutional review board protocol no. 20–023-F-E-6/12, which employed online data collection panels not requiring local review. Informed consent was obtained from participants after describing the study purpose and expected risks and benefits before participants were permitted to advance to the study questionnaire. We fully endorse the Nature Portfolio journals’ guidance on MIC authorship and inclusion. In this fourth-round study, the author list has expanded from 8 to 12 with stronger regional and gender balances. These authors (two authors from South Africa, one from Brazil, three from Spain, four from the United States and four from Poland, Germany and France) provided insights into the translation of the survey to local languages and interpretation and discussion of the results for their respective countries. We reviewed relevant studies from among the 23 studied countries in preparing the survey instrument and manuscript.

### Reporting summary

Further information on research design is available in the Nature Portfolio Reporting Summary linked to this article.

## Online content

Any methods, additional references, Nature Portfolio reporting summaries, source data, extended data, supplementary information, acknowledgements, peer review information; details of author contributions and competing interests; and statements of data and code availability are available at 10.1038/s41591-024-02939-2.

### Supplementary information

Supplementary Information

Reporting Summary

## Figures and Tables

**Extended Data Table 1:**
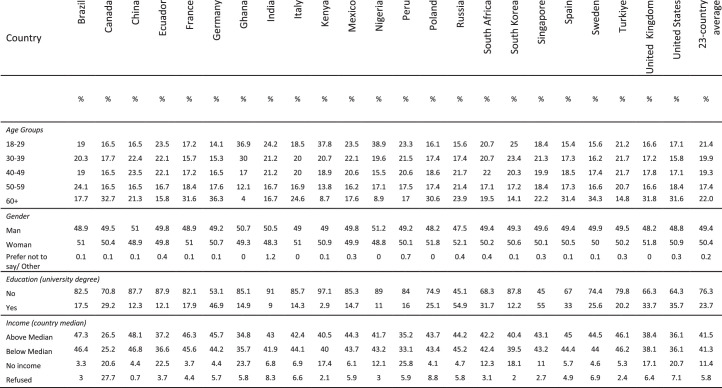
Sample characteristics by country (n = 23)

Sample size for each country (n = 1,000).

**Extended Data Table 2:**
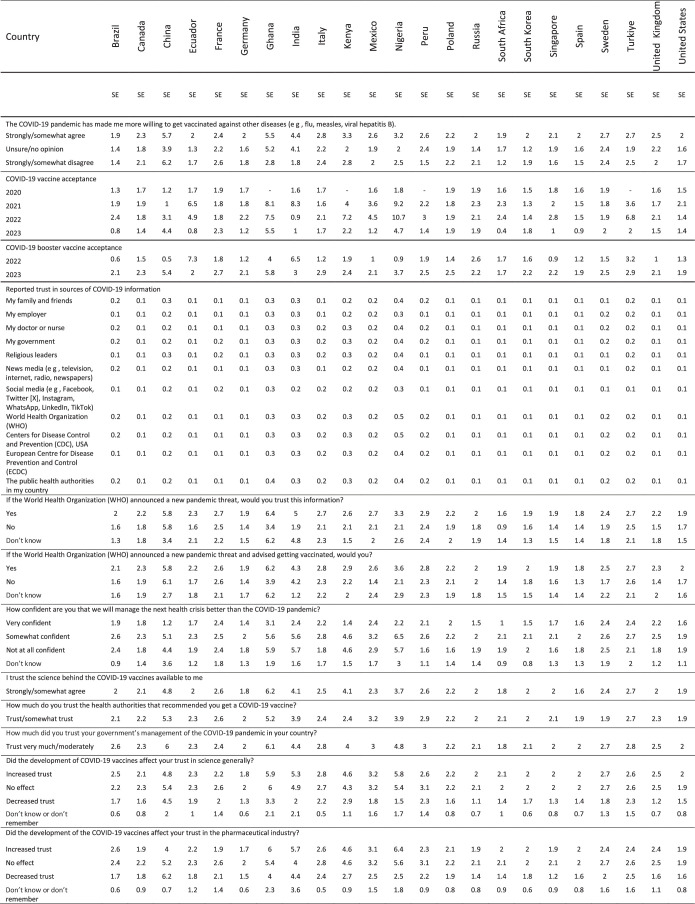
Standard errors for all variables by country (n = 23)

SE - standard error. Four countries (Ghana, Kenya, Peru and Türkiye) were not included in the 2020 global survey.

## Data Availability

The raw data generated in this study are available for download via Zenodo at 10.5281/zenodo.10568581 (ref. ^58^). All authors had access to the raw data.

## Code availability

All code for data analysis associated with the paper is available for download via Zenodo at 10.5281/zenodo.10568594 (ref. ^59^).
