# Supplementary material for: Influence of COVID-19 on trust in routine immunization, health information sources and pandemic preparedness in 23 countries in 2023
Source: Nat Med. 2024 Apr 29;30(6):1559–63. doi: 10.1038/s41591-024-02939-2 (PMC11186753; doi:10.1038/s41591-024-02939-2)
Supplement: Supplementary file 1 — Supplementary Information [file 41591_2024_2939_MOESM1_ESM.pdf]

nature medicine

Brief Communication

https://doi.org/10.1038/s41591-024-02939-2

Influence of COVID-19 on trust in routine immunization, health information sources and pandemic preparedness in 23 countries in 2023

In the format provided by the authors and unedited

Supplemental Material. Study questionnaire in each country.

Brazil 2
Canada 13
China 33
Ecuador 44
France 54
Ghana 75
India 85
Italy 106
Kenya 117
México 127
Nigeria 138
Peru 148
Poland 158
Russia 169
South Africa 180
South Korea 190
Singapore 202
Spain 212
Sweden 223
Türkiye 233
United Kingdom 243
United States 253

Brazil

COVID-VAC: Uma pesquisa global sobre as percepções da vacina para COVID-19

O objetivo dessa pesquisa é medir as reações dos residentes aos esforços de resposta à COVID-19. Os riscos nesta pesquisa de rotina são considerados mínimos. Você pode se sentir desconfortável considerando as implicações da COVID-19 e a participação neste estudo de pesquisa é totalmente voluntária e você pode interromper a participação a qualquer momento. Suas respostas permanecerão anônimas e confidenciais. A pesquisa deve levar menos de 5 minutos para ser concluída. Ao responder às perguntas, você está indicando que leu a descrição do estudo, tem mais de 18 anos e concorda com os termos descritos.

Se tiver dúvidas sobre os seus direitos como participante da pesquisa, você pode entrar em contato com o Conselho de Revisão Institucional do Emerson College (em inglês, IRB), que se preocupa com a proteção de voluntários em projetos de pesquisa. Você pode entrar em contato com o investigador principal pelo e-mail emersonpolling@emerson.edu ou com o presidente do IRB enviando um e-mail para human_subjects@emerson.edu.

Q1 "Primeiramente, indique o quanto você concorda com essa declaração: A COVID-19 ainda representa um sério risco para a saúde."

1 Concordo totalmente

2 Concordo parcialmente

3 Não tenho certeza/sem opinião

4 Discordo parcialmente

5 Discordo totalmente

Q2 "Vamos agora fazer algumas perguntas sobre as vacinas contra a COVID-19. A COVID-19 pode ser evitada através da vacinação."

1 Concordo totalmente

2 Concordo parcialmente

3 Não tenho certeza/sem opinião

4 Discordo parcialmente

5 Discordo totalmente

Q3 A doença COVID-19 representa mais riscos do que a vacina.

1 Concordo totalmente

2 Concordo parcialmente

3 Não tenho certeza/sem opinião

4 Discordo parcialmente

5 Discordo totalmente

Q4 As vacinas contra a COVID-19 que estão disponíveis para mim são seguras.

1 Concordo totalmente

2 Concordo parcialmente

3 Não tenho certeza/sem opinião

4 Discordo parcialmente

5 Discordo totalmente

Q5 Confio na ciência por trás das vacinas contra a COVID-19 que estão disponíveis para mim.

1 Concordo totalmente

2 Concordo parcialmente

3 Não tenho certeza/sem opinião

4 Discordo parcialmente

5 Discordo totalmente

Q6 As vacinas contra a COVID-19 disponíveis para mim são eficazes na prevenção de casos graves.

1 Concordo totalmente

2 Concordo parcialmente

3 Não tenho certeza/sem opinião

4 Discordo parcialmente

5 Discordo totalmente

Q7 Você recebeu pelo menos uma dose da vacina contra COVID-19?

1 Sim, recebi uma dose

2 Sim, recebi duas ou mais doses

3 Não

Q8 Quais das seguintes vacinas contra a COVID-19 você recebeu? Selecione todas as alternativas aplicáveis:

1 Moderna (Spikevax)

2 Pfizer (Comirnaty)

3 Johnson&Johnson (Janssen ou J&J)

4 Sinovac

5 Astra-Zeneca (Oxford, Vaxzevria)

6 COVAXIN

7 Outra (especifique)

8 Não sei dizer

Q9 Estou planejando tomar a vacina de reforço contra a COVID-19, conforme recomendado.

1 Concordo totalmente

2 Concordo parcialmente

3 Não tenho certeza/sem opinião

4 Discordo parcialmente

5 Discordo totalmente

Q10 Acredito que a pandemia de COVID-19 acabou.

1 Concordo totalmente

2 Concordo parcialmente

3 Não tenho certeza/sem opinião

4 Discordo parcialmente

5 Discordo totalmente

Q11 Qual foi o seu nível de confiança na administração do governo durante a pandemia de COVID-19 no seu país?

1 Não confiei

2 Não confiei em algumas coisas

3 Não tenho certeza

4 Confiei um pouco

5 Confiei

6 Não sei ou não me lembro

Q12 O desenvolvimento das vacinas contra a COVID-19 afetou a sua confiança na indústria farmacêutica?

1 Aumentou a minha confiança

2 Não teve nenhum efeito

3 Diminuiu a minha confiança

4 Não sei ou não me lembro

Q13 O desenvolvimento das vacinas contra a COVID-19 afetou a sua confiança na ciência em geral?

1 Aumentou a minha confiança

2 Não teve nenhum efeito

3 Diminuiu a minha confiança

4 Não sei ou não me lembro

Q14 Qual é o seu nível de confiança nas autoridades de saúde que recomendaram a vacinação contra a COVID-19?

1 Nenhuma

2 Um pouco

3 Moderadamente

4 Muito

5 Não sei ou não me lembro

Q15 Durante a pandemia, você teve que adiar ou cancelar algum atendimento ou serviço médico que precisava e que não estava relacionado à COVID-19?

1 Sim

2 Não

3 Não sei ou não me lembro

Q16 Futuramente, você seguiria uma exigência (obrigatoriedade) da vacinação por parte de alguma das seguintes organizações? Selecione todas as alternativas aplicáveis:

1 Setor público/governamental

2 Empregador

3 Escola ou universidade

4 Nenhum dos anteriores

5 Não sei dizer

Q17 No futuro, você seguiria uma exigência (obrigatoriedade) do uso de máscara por parte de alguma das seguintes instituições? Selecione todas as alternativas aplicáveis:

1 Setor público/governamental

2 Empregador

3 Escola ou universidade

4 Nenhum dos anteriores

5 Não sei dizer

Q18 Até onde você sabe, você tem ou já teve COVID-19?

1 Sim

2 Não

3 Não sei ou não me lembro

Q19 "A "COVID longa" foi definida como "sintomas que podem durar semanas ou meses após a recuperação de uma doença aguda".

Até onde você sabe, você tem ou já teve COVID longa? "

1 Sim

2 Não

3 Não sei ou não me lembro

Q20 Você tomou algum dos seguintes medicamentos para tratar a COVID-19? Selecione todas as alternativas aplicáveis:

1 Paxlovid

2 Molnupiravir (Lagevrio)

3 Anticorpos monoclonais (Olumiant/Baricitinibe)

4 Ivermectina

5 Medicina tradicional, extratos de ervas e terapias

6 Não sei ou não me lembro

7 Não, não tomei medicamentos para COVID-19

Q21 Algum parente ou amigo próximo seu faleceu devido à COVID-19? Selecione todas as alternativas aplicáveis:

1 Sim, no ano passado

2 Sim, há mais de um ano

3 Não

Q22 Fiquei satisfeito com os esforços de comunicação sobre as vacinas contra a COVID-19 realizados pelas autoridades de saúde do meu país.

1 Concordo totalmente

2 Concordo parcialmente

3 Não tenho certeza/não tenho opinião/não me lembro

4 Discordo parcialmente

5 Discordo totalmente

Q23 Tenho facilidade para diferenciar informações verídicas de informações falsas sobre a vacina contra a COVID-19 ao navegar na internet.

1 Concordo totalmente

2 Concordo parcialmente

3 Não tenho certeza/não tenho opinião/não me lembro

4 Discordo parcialmente

5 Discordo totalmente

Q24 Continuo me mantendo informado sobre as vacinas contra a COVID-19.

1 Concordo totalmente

2 Concordo parcialmente

3 Não tenho certeza/não tenho opinião/não me lembro

4 Discordo parcialmente

5 Discordo totalmente

Q25 Continuo atento às informações sobre vacinas em geral.

1 Concordo totalmente

2 Concordo parcialmente

3 Não tenho certeza/sem opinião

4 Discordo parcialmente

5 Discordo totalmente

Q26 A COVID-19 me incentivou a considerar a vacinação contra outras doenças (por exemplo, gripe, sarampo, hepatite B viral).

1 Concordo totalmente

2 Concordo parcialmente

3 Não tenho certeza/sem opinião

4 Discordo parcialmente

5 Discordo totalmente

Q27 A minha experiência com a vacina contra a COVID-19 afetou a minha confiança em outras vacinas.

1 Aumentou a minha confiança

2 Não teve nenhum efeito

3 Diminuiu a minha confiança

4 Não sei dizer

Q28 A cobertura dos meios de comunicação sobre as vacinas contra a COVID-19 afetou a minha confiança em outras vacinas.

1 Aumentou a minha confiança

2 Não teve nenhum efeito

3 Diminuiu a minha confiança

4 Não sei dizer

Q29 A cobertura das redes sociais sobre as vacinas contra a COVID-19 afetou a minha confiança em relação a outras vacinas.

1 Aumentou a minha confiança

2 Não teve nenhum efeito

3 Diminuiu a minha confiança

4 Não sei dizer

Q30 Caso uma vacina de RNAm seja aprovada para uma doença para a qual você tem predisposição, você a tomaria?

1 Definitivamente não

2 Não tenho certeza, mas provavelmente não

3 Não tenho certeza, mas provavelmente sim

4 Definitivamente sim

Q31 Se a Organização Mundial da Saúde (OMS) anunciasse uma nova ameaça de pandemia, você confiaria nessa informação?

1 Sim

2 Não

3 Não sei dizer

Q32 Se a Organização Mundial da Saúde (OMS) anunciasse uma nova ameaça de pandemia e recomendasse a vacinação, você seguiria essa orientação?

1 Sim

2 Não

3 Não sei dizer

Q33 "Em uma escala de 1 (confio totalmente) a 10 (nenhuma confiança), o quanto você confia nas seguintes fontes de informação sobre Vacinas contra a COVID-10: Minha família e meus amigos"

Q34 Meu empregador

Q35 Meu médico ou enfermeiro

Q36 Meu governo

Q37 Líderes religiosos

Q38 Meios de comunicação e notícias (incluindo televisão, internet, rádio, jornais)

Q39 Redes sociais (como o Facebook, Twitter [X], Instagram, WhatsApp, LinkedIn, TikTok)

Q40 Organização Mundial da Saúde (OMS)

Q41 Agências reguladoras de saúde como a Agência Nacional de Saúde (ANS) no Brasil e os Centros de Controle e Prevenção de Doenças (CDC) nos Estados Unidos

Q42 O Centro Europeu de Prevenção e Controle de Doenças (ECDC)

Q43 As autoridades de saúde pública do meu país

Q44 Qual é o seu nível de confiança de que enfrentaremos a próxima crise de saúde de forma mais eficaz em comparação com a pandemia de COVID-19?

1 Nem um pouco confiante

2 Um pouco confiante

3 Muito confiante

4 Não sei dizer

Q45 Com base nas informações que você já leu ou ouviu, você acredita que as mudanças climáticas e o aquecimento global sejam uma ameaça concreta, ou não?

1 É uma ameaça real

2 Não é uma ameaça real

Q46 Qual é a sua idade?

Q47 Qual é o seu sexo?

1 Masculino

2 Feminino

3 Prefiro não dizer

Q48 Escolaridade (específico do país)

1 Ensino médio incompleto

2 Ensino médio concluído

3 Universidade/Ensino técnico incompleto

4 Ensino técnico ou profissionalizante concluído

5 Educação universitária concluída

6 Pós-graduação concluída

7 Doutorado, Pós-doutorado ou equivalente concluído

Q49 Nível de renda mediana (específico do país)

1 Sim, a minha renda mensal é superior a 3.225 reais.

2 Sim, minha renda mensal é inferior a 3.225 reais.

3 Não, eu não tenho renda.

4 Recusou/Não respondeu

Q50 Regiões (específico do país)

1 Região Norte, Estados: Acre, Amapá, Amazonas, Pará, Rondônia, Roraima, Tocantins

2 Região Nordeste, Estados: Alagoas, Bahia, Ceará, Maranhão, Paraíba, Pernambuco, Piauí, Rio Grande do Norte, Sergipe

3 Centro-Oeste (Região Central), Estados: Goiás, Mato Grosso, Mato Grosso do Sul, Distrito Federal (Distrito Federal).

4 Região Sudeste, Estados: Espírito Santo, Minas Gerais, Rio de Janeiro, São Paulo

5 Região Sul. Estados: Paraná, Rio Grande do Sul, Santa Catarina

Canada

COVID-VAC: Une enquête mondiale sur la perception du vaccin contre la COVID-19

L'objectif de cette enquête est d'évaluer la réaction des résidents face aux mesures prises en réponse à la COVID-19. Les risques de cette enquête de routine sont considérés comme minimes. Il se peut que vous vous sentiez mal à l'aise par rapport aux répercussions de la COVID-19. Votre participation à cette étude de recherche est entièrement volontaire. Vous pouvez mettre un terme à votre participation à tout moment. Vos réponses resteront anonymes et confidentielles. Répondre à cette enquête devrait vous prendre moins de 5 minutes. En répondant aux questions, vous indiquez que vous avez lu la description de l'étude, que vous avez plus de 18 ans et que vous acceptez les conditions générales décrites.

Si vous avez des questions à propos de vos droits en tant que participant(e) à l'enquête, vous pouvez contacter le comité de protection des personnes (IRB) de l'Emerson College, s'occupant de la protection des participants volontaires aux projets de recherche. Vous pouvez contacter le chercheur principal par e-mail à l'adresse emersonpolling@emerson.edu ou le conseil de l'IRB en envoyant un e-mail à l'adresse human_subjects@emerson.edu.

Q1 "Tout d'abord, veuillez indiquer votre degré d'accord avec cette affirmation: La COVID-19 demeure une menace dangereuse pour la santé."

1 Fortement d’accord

2 Plutôt d’accord

3 Incertain(e)/aucune opinion

4 Plutôt en désaccord

5 Fortement en désaccord

Q2 "Voici maintenant quelques questions relatives aux vaccins contre la COVID-19. La vaccination permet d'éviter la COVID-19."

1 Fortement d’accord

2 Plutôt d’accord

3 Incertain(e)/aucune opinion

4 Plutôt en désaccord

5 Fortement en désaccord

Q3 Les risques de la COVID-19 sont plus importants que les risques du vaccin.

1 Fortement d’accord

2 Plutôt d’accord

3 Incertain(e)/aucune opinion

4 Plutôt en désaccord

5 Fortement en désaccord

Q4 Les vaccins contre la COVID-19 dont je dispose sont sûrs.

1 Fortement d’accord

2 Plutôt d’accord

3 Incertain(e)/aucune opinion

4 Plutôt en désaccord

5 Fortement en désaccord

Q5 Je fais confiance à la science qui sous-tend les vaccins COVID-19 mis à ma disposition.

1 Fortement d’accord

2 Plutôt d’accord

3 Incertain(e)/aucune opinion

4 Plutôt en désaccord

5 Fortement en désaccord

Q6 Les vaccins contre la COVID-19 dont je dispose sont efficaces pour protéger contre les formes graves de la COVID-19.

1 Fortement d'accord

2 Plutôt d'accord

3 Incertain(e)/aucune opinion

4 Plutôt en désaccord

5 Fortement en désaccord

Q7 Avez-vous reçu au moins une dose de vaccin contre la COVID-19?

1 Oui, j'ai reçu une dose

2 Oui, j'ai reçu deux doses ou plus

3 Non

Q8 Lequel des vaccins contre la COVID-19 suivants avez-vous reçu? Sélectionnez tout ce qui convient :

1 Moderna (Spikevax)

2 Pfizer (Comirnaty)

3 Johnson&Johnson (Janssen ou J&J)

4 Sinovac

5 Astra-Zeneca (Oxford, Vaxzevria)

6 COVAXIN

7 Autre (veuillez préciser)

8 Je ne sais pas

Q9 Je prendrai le vaccin de rappel contre la COVID-19 recommandé.

1 Fortement d'accord

2 Plutôt d'accord

3 Incertain(e)/aucune opinion

4 Plutôt en désaccord

5 Fortement en désaccord

Q10 Je crois que la pandémie de COVID-19 est terminée.

1 Fortement d'accord

2 Plutôt d'accord

3 Incertain(e)/aucune opinion

4 Plutôt en désaccord

5 Fortement en désaccord

Q11 Dans quelle mesure avez-vous eu confiance dans la gestion par votre gouvernement de la pandémie de COVID-19 dans votre pays?

1 Aucune confiance

2 Peu confiance

3 Incertain(e)

4 Assez confiance

5 Confiance

6 Je ne sais pas ou je ne me souviens pas

Q12 Le développement des vaccins contre la COVID-19 a-t-il affecté votre confiance dans l'industrie pharmaceutique?

1 Meilleure confiance

2 Aucun effet

3 Moindre confiance

4 Je ne sais pas ou je ne me souviens pas

Q13 Le développement des vaccins contre la COVID-19 a-t-il affecté votre confiance dans la science en général?

1 Meilleure confiance

2 Aucun effet

3 Moindre confiance

4 Je ne sais pas ou je ne me souviens pas

Q14 Dans quelle mesure faites-vous confiance aux autorités sanitaires qui vous ont recommandé de vous faire vacciner contre la COVID-19?

1 Pas du tout confiance

2 Peu confiance

3 Moyennement confiance

4 Grande confiance

5 Je ne sais pas ou je ne me souviens pas

Q15 Pendant la pandémie, avez-vous dû retarder ou annuler des services médicaux dont vous aviez besoin et qui n'étaient pas liés à la COVID-19?

1 Oui

2 Non

3 Je ne sais pas ou je ne me souviens pas

Q16 Respecteriez-vous à l'avenir une obligation vaccinale (mandat) émanant de l'un des organismes suivants? Sélectionnez tout ce qui convient:

1 Gouvernement

2 Employeur

3 École ou université

4 Aucun

5 Je ne sais pas

Q17 Respecteriez-vous à l'avenir l'obligation de porter un masque de protection (mandat) imposée par l'un des organismes suivants? Sélectionnez tout ce qui convient:

1 Gouvernement

2 Employeur

3 École ou université

4 Aucun

5 Je ne sais pas

Q18 A votre connaissance, avez-vous ou avez-vous déjà eu la COVID-19?

1 Oui

2 Non

3 Je ne sais pas ou je ne me souviens pas

Q19 "Le COVID longue a été définie comme « des symptômes qui peuvent durer des semaines ou des mois après la guérison d'une maladie aiguë ».

À votre connaissance, avez-vous ou avez-vous déjà eu la COVID longue? "

1 Oui

2 Non

3 Je ne sais pas ou je ne me souviens pas

Q20 Avez-vous pris l'un des médicaments suivants pour traiter la COVID-19? Sélectionnez tout ce qui convient:

1 Paxlovid

2 Molnupiravir (Lagevrio)

3 Anticorps monoclonaux (Olumiant/Baricitinib)

4 Ivermectin

5 Médicament traditionnel, extraits et traitements à base de plantes

6 Je ne sais pas ou je ne me souviens pas

7 Non, je n'ai pas pris de médicaments contre la COVID-19

Q21 Avez-vous perdu un membre de votre famille ou un ami proche à cause de la COVID-19? Sélectionnez tout ce qui convient:

1 Oui, au cours de la dernière année

2 Oui, il y a plus d'un an

3 Non

Q22 Je suis satisfait(e) des efforts de communication sur les vaccins contre la COVID-19 déployés par les autorités sanitaires de mon pays.

1 Fortement d'accord

2 Plutôt d'accord

3 Incertain(e)/aucune opinion/ne se souvient pas

4 Plutôt en désaccord

5 Fortement en désaccord

Q23 Il m'est facile de faire la différence entre des informations exactes et des informations erronées sur le vaccin contre la COVID-19 sur Internet.

1 Fortement d'accord

2 Plutôt d'accord

3 Incertain(e)/aucune opinion/ne se souvient pas

4 Plutôt en désaccord

5 Fortement en désaccord

Q24 Je prête attention aux informations concernant les vaccins contre la COVID-19.

1 Fortement d'accord

2 Plutôt d'accord

3 Incertain(e)/aucune opinion/ne se souvient pas

4 Plutôt en désaccord

5 Fortement en désaccord

Q25 Je continue à prêter attention aux informations concernant les vaccins en général.

1 Fortement d'accord

2 Plutôt d'accord

3 Incertain(e)/aucune opinion

4 Plutôt en désaccord

5 Fortement en désaccord

Q26 La pandémie de COVID-19 m'a incité(e) à me faire vacciner contre d'autres maladies (grippe, rougeole, hépatite virale B).

1 Fortement d'accord

2 Plutôt d'accord

3 Incertain(e)/aucune opinion

4 Plutôt en désaccord

5 Fortement en désaccord

Q27 Mon expérience avec le vaccin contre la COVID-19 a affecté ma confiance envers les autres vaccins.

1 Meilleure confiance

2 Aucun effet

3 Moindre confiance

4 Je ne sais pas

Q28 La couverture médiatique des vaccins contre la COVID-19 a affecté ma confiance envers les autres vaccins.

1 Meilleure confiance

2 Aucun effet

3 Moindre confiance

4 Je ne sais pas

Q29 La couverture médiatique des vaccins contre la COVID-19 sur les médias sociaux a affecté ma confiance envers les autres vaccins.

1 Meilleure confiance

2 Aucun effet

3 Moindre confiance

4 Je ne sais pas

Q30 Si un vaccin à ARNm est approuvé pour une maladie pour laquelle vous êtes à risque, le prendriez-vous?

1 Certainement pas

2 Incertain(e), mais j’aurais tenance à dire non

3 Incertain(e), mais j’aurais tendance à dire oui

4 Certainement

Q31 Si l'Organisation mondiale de la santé (OMS) annonçait une nouvelle menace de pandémie, feriez-vous confiance à cette information?

1 Oui

2 Non

3 Je ne sais pas

Q32 Si l'Organisation mondiale de la santé (OMS) annonçait une nouvelle menace de pandémie et conseillait de se faire vacciner, le feriez-vous?

1 Oui

2 Non

3 Je ne sais pas

Q33 "Sur une échelle de 1 (tout à fait confiance) à 10 (pas du tout confiance), quelle confiance accordez-vous aux sources d'information suivantes concernant Vaccins contre la COVID-19: Ma famille et mes amis"

Q34 Mon employeur

Q35 Mon médecin ou un membre du personnel infirmier

Q36 Mon gouvernement

Q37 Chefs religieux

Q38 Médias d'information (télévision, Internet, radio, journaux)

Q39 Médias sociaux (par exemple, Facebook, Twitter [X], Instagram, WhatsApp, LinkedIn, TikTok)

Q40 Organisation mondiale de la santé (OMS)

Q41 Centers for Disease Control and Prevention (CDC), État-Unis

Q42 Centre européen de prévention et de contrôle des maladies (ECDC)

Q43 Les autorités de santé publique de mon pays

Q44 Dans quelle mesure êtes-vous convaincu(e) que la prochaine crise sanitaire sera mieux gérée que la pandémie de COVID-19?

1 Pas du tout convaincu(e)

2 Assez convaincu(e)

3 Tout à fait convaincu(e)

4 Je ne sais pas

Q45 D'après ce que vous avez lu ou entendu, pensez-vous que le changement climatique/réchauffement de la planète constitue une menace réelle ou non?

1 Réelle menace

2 Pas une réelle menace

Q46 Quel âge avez-vous?

Q47 À quelle identité de genre vous associez-vous?

1 Homme

2 Femme

3 Je préfère ne pas répondre

Q48 Éducation (spécifique au pays)

1 Bac (général, professionnel et technologique) ou moins

2 Bac +2 (brevet de technicient supérieur ou autre)

3 Bac+3/4 (baccalauréat, maîtrise)

4 Bac+5 (maîtrise, écoles d'ingénieur, écoles d'art…)

5 Bac+7 (doctorat, post-doctorat, thèse)

Q49 Niveau de revenu moyen (spécifique au pays)

1 Oui, mon revenu mensuel est supérieur à 5 820 dollars canadiens.

2 Oui, mon revenu mensuel est inférieur à 5 820 dollars canadiens.

3 Non, je n'ai pas de revenu.

4 Refuse de répondre/N'a pas répondue

Q50 Régions (spécifique au pays)

1 "Les provinces de l'Atlantique: Nouveau-Brunswick, Terre-Neuve-et-Labrador, Nouvelle-Écosse, île-du-Prince-Édouard"

2 Centre du Canada: Ontario et Québec

3 Les provinces des Prairies: Alberta, Saskatchewan et Manitoba

4 La côte ouest: Colombie-Britannique

5 Les Territoires du Nord.

CANADA

COVID-VAC: A global survey of COVID-19 vaccine perceptions

The purpose of the survey is to measure resident's reactions to COVID-19 response efforts. The risks in this routine survey are considered minimal. You may feel uncomfortable considering the implications of COVID-19 and participation in this research study is completely voluntary and you may discontinue participation at any time. Your responses will remain anonymous and confidential. The survey should take less than 5 minutes to complete. By answering the questions, you are indicating that you have read the description of the study, are over the age of 18, and that you agree to the terms as described.

If you have questions about your rights as a research participant, you may contact the Emerson College Institutional Review Board (IRB), which is concerned with the protection of volunteers in research projects. You may reach the lead investigator via email at emersonpolling@emerson.edu or the Chair of the IRB by e-mailing human_subjects@emerson.edu.

Q1 "First, please indicate your level of agreement with this statement: COVID-19 remains a dangerous health threat."

1 Strongly agree

2 Somewhat agree

3 Unsure/no opinion

4 Somewhat disagree

5 Strongly disagree

Q2 "Now, here are some questions related to COVID-19 vaccines. COVID-19 can be prevented by vaccination."

1 Strongly agree

2 Somewhat agree

3 Unsure/no opinion

4 Somewhat disagree

5 Strongly disagree

Q3 The risks of COVID-19 disease are greater than the risks of the vaccine.

1 Strongly agree

2 Somewhat agree

3 Unsure/no opinion

4 Somewhat disagree

5 Strongly disagree

Q4 The COVID-19 vaccines available to me are safe.

1 Strongly agree

2 Somewhat agree

3 Unsure/no opinion

4 Somewhat disagree

5 Strongly disagree

Q5 I trust the science behind the COVID-19 vaccines available to me.

1 Strongly agree

2 Somewhat agree

3 Unsure/no opinion

4 Somewhat disagree

5 Strongly disagree

Q6 The COVID-19 vaccines available to me are effective in protecting against severe COVID-19.

1 Strongly agree

2 Somewhat agree

3 Unsure/no opinion

4 Somewhat disagree

5 Strongly disagree

Q7 Have you received at least one dose of a COVID-19 vaccine?

1 Yes, I received one dose

2 Yes, I received two or more doses

3 No

Q8 Which of the following COVID-19 vaccines did you receive? Select all that apply:

1 Moderna (Spikevax)

2 Pfizer (Comirnaty)

3 Johnson&Johnson (Janssen or J&J)

4 Sinovac

5 Astra-Zeneca (Oxford, Vaxzevria)

6 COVAXIN

7 Other (please specify)

8 Don’t know

Q9 I will take the recommended COVID-19 booster.

1 Strongly agree

2 Somewhat agree

3 Unsure/no opinion

4 Somewhat disagree

5 Strongly disagree

Q10 I believe the COVID-19 pandemic is over.

1 Strongly agree

2 Somewhat agree

3 Unsure/no opinion

4 Somewhat disagree

5 Strongly disagree

Q11 How much did you trust your government's management of the COVID-19 pandemic in your country?

1 Did not trust

2 Somewhat did not trust

3 Unsure

4 Somewhat trust

5 Trust

6 Don't know or don't remember

Q12 Did the development of the COVID-19 vaccines affect your trust in the pharmaceutical industry?

1 Increased trust

2 No effect

3 Decreased trust

4 Don't know or don't remember

Q13 Did the development of COVID-19 vaccines affect your trust in science generally?

1 Increased trust

2 No effect

3 Decreased trust

4 Don't know or don't remember

Q14 How much do you trust the health authorities that recommended you get a COVID-19 vaccine?

1 Not at all

2 A little

3 Moderately

4 Very much

5 Don’t know or don’t remember

Q15 During the pandemic, did you have to delay or cancel any medical services that you needed unrelated to COVID-19?

1 Yes

2 No

3 Don’t know or don’t remember

Q16 Would you follow a vaccine requirement (mandate) in the future from any of the following? Select all that apply:

1 Government

2 Employer

3 School or university

4 None of the above

5 Don’t know

Q17 Would you follow a facemask requirement (mandate) in the future from any of the following? Select all that apply:

1 Government

2 Employer

3 School or university

4 None of the above

5 Don’t know

Q18 To your knowledge, do you have or have you had COVID-19?

1 Yes

2 No

3 Don’t know or don’t remember

Q19 "Long-COVID has been defined as "symptoms that can last for weeks or months after recovery from acute illness". To your knowledge, do you have or have you had Long COVID?"

1 Yes

2 No

3 Don't know or don't remember

Q20 Did you take any of the following medicines to treat COVID-19? Select all that apply:

1 Paxlovid

2 Molnupiravir (Lagevrio)

3 Monoclonal antibodies (Olumiant/Baricitinib)

4 Ivermectin

5 Traditional medicine, herbal extracts and treatments

6 Don't know or don't remember

7 No, I did not take medicines for COVID-19

Q21 Have you lost a family member or close friend to COVID-19 disease? Select all that apply:

1 Yes, within the past year

2 Yes, more than a year ago

3 No

Q22 I was satisfied with the communication efforts on COVID-19 vaccines made by health authorities in my country.

1 Strongly agree

2 Somewhat agree

3 Unsure/no opinion/don't remember

4 Somewhat disagree

5 Strongly disagree

Q23 It is easy for me to know the difference between accurate and false information about the COVID-19 vaccine on the internet.

1 Strongly agree

2 Somewhat agree

3 Unsure/no opinion/don't remember

4 Somewhat disagree

5 Strongly disagree

Q24 I continue to pay attention to information on COVID-19 vaccines.

1 Strongly agree

2 Somewhat agree

3 Unsure/no opinion/don’t remember

4 Somewhat disagree

5 Strongly disagree

Q25 I continue to pay attention to information on vaccines in general.

1 Strongly agree

2 Somewhat agree

3 Unsure/no opinion

4 Somewhat disagree

5 Strongly disagree

Q26 The COVID-19 pandemic has made me more willing to get vaccinated against other diseases (e.g., flu, measles, viral hepatitis B).

1 Strongly agree

2 Somewhat agree

3 Unsure/no opinion

4 Somewhat disagree

5 Strongly disagree

Q27 My experience with the COVID-19 vaccine has affected my trust in other vaccines.

1 Increased trust

2 No effect

3 Decreased trust

4 Don’t know

Q28 Broadcast media coverage on COVID-19 vaccines has affected my trust in other vaccines.

1 Increased trust

2 No effect

3 Decreased trust

4 Don’t know

Q29 Social media coverage on COVID-19 vaccines has affected my trust in other vaccines.

1 Increased trust

2 No effect

3 Decreased trust

4 Don’t know

Q30 If an mRNA vaccine is approved for a disease for which you are at risk, would you take it?

1 Definitely no

2 Unsure, but leaning towards no

3 Unsure, but leaning towards yes

4 Definitely yes

Q31 If the World Health Organization (WHO) announced a new pandemic threat, would you trust this information?

1 Yes

2 No

3 Don’t know

Q32 If the World Health Organization (WHO) announced a new pandemic threat and advised getting vaccinated, would you?

1 Yes

2 No

3 Don’t know

Q33 "On a scale of 1 (trust completely) to 10 (do not trust at all) how much do you trust the following sources of information about COVID-19 vaccines: My family and friends"

Q34 My employer

Q35 My doctor or nurse

Q36 My government

Q37 Religious leaders

Q38 News media (e.g., television, internet, radio, newspapers)

Q39 Social media (e.g., Facebook, Twitter [X], Instagram, WhatsApp, LinkedIn, TikTok)

Q40 World Health Organization (WHO)

Q41 Centers for Disease Control and Prevention (CDC), USA

Q42 European Centre for Disease Prevention and Control (ECDC)

Q43 The public health authorities in my country

Q44 How confident are you that we will manage the next health crisis better than the COVID-19 pandemic?

1 Not at all confident

2 Somewhat confident

3 Very confident

4 Don’t know

Q45 From what you have read or heard, do you think climate change/global warming is a real threat or not a real threat?

1 Real threat

2 Not a real threat

Q46 What is your age?

Q47 What is your gender?

1 Male

2 Female

3 Prefer not to say

Q48 Education (country specific)

1 High school or less

2 Vocational degree

3 Some university or three-year degree

4 University graduate (four-year honours degree)

5 Post-university graduate (Master's, Doctorate, Law)

Q49 Median Income Level (country specific)

1 Yes, my monthly income is more than $5,820 Canadian

2 Yes, my monthly income is more than $5,820 Canadian

3 No, I do not have an income

4 Refused/ Did not answer

Q50 Regions (country specific)

1 Atlantic Provinces: New Brunswick, Newfoundland and Labrador, Nova Scotia, Prince Edward Island

2 Central Canada: Ontario and Quebec

3 Prairie Provinces: Alberta, Saskatchewan, and Manatoba

4 West Coast: British Columbia

5 Northern Territories

China

新冠病毒疫苗： 对新冠病毒疫苗认知的全球调查

标题：新冠病毒疫苗： 对新冠病毒疫苗认知的全球调查

本调查的目的，是衡量居民对新冠病毒应对措施的反应。 **本常**规调查的风险极小。 **您考**虑到新冠病毒时可能会感觉不适。参加本研究完全自愿，您可以随时停止参与。 **您的回答将保持匿名和保密。 完成本**调查最多需要 5 **分**钟。 **回答**这些问题，即表示您已阅读研究说明，并已年满 18 岁以上，并同意所述条款。

如果您对自己作为研究参加者的权利有任何疑问，请联系爱默生学院机构审查委员会 (IRB)， 该委员会关注研究项目中志愿者的保护问题。 **您**还可以通过电子邮件联系主要研究者：emersonpolling@emerson.edu **或** IRB **主席**：human_subjects@emerson.edu.

Q1 **"首先**，请表明您在多大程度上同意这一陈述：**新冠肺炎仍然是危**险的健康威胁。**"**

1 强烈同意

2 有些同意

3 不确定/无意见

4 有些不同意

5 强烈不同意

Q2 **"下面是几个与新冠肺炎疫苗有关的**问题。**新冠肺炎可以通**过疫苗预防。**"**

1 强烈同意

2 有些同意

3 不确定/无意见

4 有些不同意

5 强烈不同意

Q3 **新冠肺炎的**风险大于疫苗的风险。

1 强烈同意

2 有些同意

3 不确定/无意见

4 有些不同意

5 强烈不同意

Q4 **向我提供的新冠肺炎疫苗是安全的。**

1 强烈同意

2 有些同意

3 不确定/无意见

4 有些不同意

5 强烈不同意

Q5 我信任向我提供的新冠肺炎疫苗所依托的科学。

1 强烈同意

2 有些同意

3 不确定/无意见

4 有些不同意

5 强烈不同意

Q6 向我提供的新冠肺炎疫苗能有效地预防严重的新冠肺炎。

1 强烈同意

2 有些同意

3 不确定/无意见

4 有些不同意

5 强烈不同意

Q7 您是否已至少接种过一剂新冠肺炎疫苗？

1 是的，我接种过一剂

2 是的，我接种过两剂或以上

3 否

Q8 您接种过以下哪些新冠肺炎疫苗？请选择全部适用项：

1 莫德纳 (Spikevax)

2 辉瑞 (Comirnaty)

3 强生（Janssen 或 J&J）

4 科星

5 阿斯利康 (Oxford, VAxzevria)

6 COVAXIN

7 其他（请说明）

8 不知道

Q9 我会接种建议的新冠肺炎加强针。

1 强烈同意

2 有些同意

3 不确定/无意见

4 有些不同意

5 强烈不同意

Q10 我认为新冠肺炎大流行已经结束。

1 强烈同意

2 有些同意

3 不确定/无意见

4 有些不同意

5 强烈不同意

Q11 您在多大程度上信任您的政府在贵国应对新冠肺炎大流行方面的管理？

1 不信任

2 有些不信任

3 不确定

4 有些信任

5 信任

6 不知道或不记得

Q12 新冠肺炎疫苗的开发是否影响了您对制药行业的信任？

1 增加了信任

2 无影响

3 减少了信任

4 不知道或不记得

Q13 新冠肺炎疫苗的开发是否影响了您整体上对科学的信任？

1 增加了信任

2 无影响

3 减少了信任

4 不知道或不记得

Q14 您在多大程度上信任建议您接种新冠肺炎疫苗的卫生机构？

1 完全没有

2 有一点

3 中等

4 很多

5 不知道或不记得

Q15 在疫情期间，您是否推迟或取消了您需要的，但与新冠肺炎无关的医疗服务？

1 是

2 否

3 不知道或不记得

Q16 您将来是否会遵循来自以下任何一方的疫苗要求（强制性规定）？ 请选择全部适用项：

1 政府

2 雇主

3 学校或大学

4 以上皆非

5 不知道

Q17 您将来是否会遵循来自以下任何一方的口罩要求（强制性规定）？ 请选择全部适用项：

1 政府

2 雇主

3 学校或大学

4 以上皆非

5 不知道

Q18 据您所知，您是否患有或曾经患有新冠肺炎？

1 是

2 否

3 不知道或不记得

Q19 "长新冠的定义是："从急性病期恢复后，症状可持续数周或数月。"

据您所知，您是否患有或曾经患有长新冠？"

1 是

2 否

3 不知道或不记得

Q20 您是否服用过以下药物来治疗新冠肺炎？请选择全部适用项：

1 Paxlovid

2 莫努匹韦 (Lagevrio)

3 单克隆抗体 (Olumiant/Baricitinib)

4 伊维菌素

5 传统药物、草药提取物和治疗

6 不知道或不记得

7 否，我未服用药物来治疗新冠肺炎

Q21 您是否有家人或好友因罹患新冠肺炎去世？请选择全部适用项：

1 是的，在去年

2 是的，在一年以前

3 否

Q22　我对我国卫生部门在新冠肺炎疫苗方面的宣传工作感到满意。

1　强烈同意

2　有些同意

3　不确定/无意见/不记得

4　有些不同意

5　强烈不同意

Q23　我能够轻松分辨互联网上有关新冠肺炎疫苗的准确信息和错误信息。

1　强烈同意

2　有些同意

3　不确定/无意见/不记得

4　有些不同意

5　强烈不同意

Q24　我继续关注有关新冠肺炎疫苗的信息。

1　强烈同意

2　有些同意

3　不确定/无意见/不记得

4　有些不同意

5　强烈不同意

Q25　我继续关注有关疫苗的整体信息。

1　强烈同意

2　有些同意

3　不确定/无意见

4　有些不同意

5　强烈不同意

Q26 **新冠肺炎疫情**让我更愿意接种针对其他疾病的疫苗（如：流感、麻疹、乙型肝炎）。

1 强烈同意

2 有些同意

3 不确定/无意见

4 有些不同意

5 强烈不同意

Q27 **我的新冠肺炎疫苗**经历影响了我对其他疫苗的信任。

1 增加了信任

2 无影响

3 减少了信任

4 不知道

Q28 **广播媒体**对新冠肺炎疫苗的报道影响了我对其他疫苗的信任。

1 增加了信任

2 无影响

3 减少了信任

4 不知道

Q29 **社交媒体**对新冠肺炎疫苗的报道影响了我对其他疫苗的信任。

1 增加了信任

2 无影响

3 减少了信任

4 不知道

Q30 **如果**针对某种疾病的 **mRNA 疫苗**获得批准，而您有患病风险，您会接种吗？

1 肯定不接种

2 不确定，但倾向于不接种

3 不确定，但倾向于接种

4 肯定接种

Q31 如果世界卫生组织 (WHO) 宣布发生新的疫情威胁，您会信任该信息吗？

1 是

2 否

3 不知道

Q32 如果世界卫生组织 (WHO) 宣布发生新的疫情威胁，并建议接种疫苗，您会接种吗？

1 是

2 否

3 不知道

Q33 "请用 1（完全信任）到 10（完全不信任）的分值来评价您对以下信息来源关于新冠肺炎疫苗的信任程度。新冠肺炎疫苗：我的家人和朋友"

Q34 我的雇主

Q35 我的医生或护士

Q36 我的政府

Q37 宗教领袖

Q38 新闻媒体（如：电视、互联网、电台、报纸）

Q39 社交媒体（如：Facebook、Twitter [X]、Instagram、WhatsApp、LinkedIn、TikTok）

Q40 世界卫生组织 (WHO)

Q41 美国疾病控制与预防中心 **(CDC)**

Q42 欧洲疾病预防与控制中心 **(ECDC)**

Q43 我国的公共卫生部门

Q44 您对我们可以比新冠肺炎大流行更好地应对下一次卫生危机有多大信心？

1 完全无信心

2 有些信心

3 非常有信心

4 不知道

Q45 根据您读到或听到的信息，您认为气候变化**/全球**变暖是否为真实威胁？

1 真实威胁

2 并非真实威胁

Q46 您多大年纪？

Q47 您的性别是？

1 男性

2 女性

3 不愿回答

Q48 教育（针对具体国家）

1 小学

2 初中

3 高中（中学）

4 职业学校

5 大学或学院

6 已完成研究生教育

7 已完成博士、博士后或同等学历

Q49 中位收入水平（针对具体国家）

1 是的，我的月收入高于 1412 元人民币。

2 是的，我的月收入低于 1412 元人民币。

3 不，我没有收入。

4 拒绝回答/未回答

Q50 地区（根据具体国家）

1 东北及附近 (北京，河北，黑龙江，吉林，辽宁，山东，天津）;

2 北方中部 （内蒙古，山西）；

3 西北（甘肃，宁夏，陕西，青海，新疆）；

4 西南（重庆，广西，贵州，四川，西藏，云南）；

5 中南部 （安徽，河南，湖北，湖南，江西）

6 东南 （福建，广东，海南，江苏，上海，浙江）.

Ecuador

COVID-VAC: Una encuesta mundial sobre la percepción de la vacuna contra la COVID-19

El propósito de la encuesta es medir las reacciones de los residentes a los esfuerzos de respuesta de la COVID-19. Los riesgos de esta encuesta rutinaria se consideran mínimos. Es posible que se sienta incómodo al considerar las implicaciones de la COVID-19 y la participación en este estudio de investigación es completamente voluntaria y puede interrumpir su participación en cualquier momento. Sus respuestas serán anónimas y confidenciales. La encuesta debería durar menos de 5 minutos. Al responder a las preguntas, indica que ha leído la descripción del estudio, que es mayor de 18 años y que acepta los términos descritos.

Si tiene preguntas sobre sus derechos como participante en una investigación, puede ponerse en contacto con la Junta de Revisión Institucional (IRB) de Emerson College, que se ocupa de la protección de los voluntarios en los proyectos de investigación. Puede ponerse en contacto con el investigador principal a través del correo electrónico emersonpolling@emerson.edu o con el Presidente del IRB a través del correo electrónico human_subjects@emerson.edu.

Q1 "En primer lugar, por favor, indique su nivel de acuerdo con esta afirmación: El COVID-19 sigue siendo una peligrosa amenaza para la salud."

1 Sumamente de acuerdo

2 Algo de acuerdo

3 No estoy seguro(a)/sin opinión

4 Algo en desacuerdo

5 Sumamente en desacuerdo

Q2 "Ahora, estas son algunas preguntas relacionadas con las vacunas contra el COVID-19. El COVID-19 se puede prevenir con vacunación."

1 Sumamente de acuerdo

2 Algo de acuerdo

3 No estoy seguro(a)/sin opinión

4 Algo en desacuerdo

5 Sumamente en desacuerdo

Q3 Los riesgos de la enfermedad del COVID-19 son mayores que los riesgos de la vacuna.

1 Sumamente de acuerdo

2 Algo de acuerdo

3 No estoy seguro(a)/sin opinión

4 Algo en desacuerdo

5 Sumamente en desacuerdo

Q4 Las vacunas para el COVID-19 que hay disponibles para mí son seguras.

1 Sumamente de acuerdo

2 Algo de acuerdo

3 No estoy seguro(a)/sin opinión

4 Algo en desacuerdo

5 Sumamente en desacuerdo

Q5 Confío en la ciencia que hay detrás de las vacunas para el COVID-19 que hay disponibles para mí.

1 Sumamente de acuerdo

2 Algo de acuerdo

3 No estoy seguro(a)/sin opinión

4 Algo en desacuerdo

5 Sumamente en desacuerdo

Q6 Las vacunas para el COVID-19 que hay disponibles para mí son efectivas protegiéndome del COVID-19 grave.

1 Sumamente de acuerdo

2 Algo de acuerdo

3 No estoy seguro(a)/sin opinión

4 Algo en desacuerdo

5 Sumamente en desacuerdo

Q7 ¿Ha recibido usted como mínimo una dosis de vacuna para el COVID-19?

1 Sí, recibí una dosis

2 Sí, recibí dos o más dosis

3 No

Q8 ¿Cuál o cuáles de las siguientes vacunas para el COVID-19 recibió? Seleccione todas las opciones que correspondan:

1 Moderna (Spikevax)

2 Pfizer (Comirnaty)

3 Johnson&Johnson (Janssen o J&J)

4 Sinovac

5 Astra-Zeneca (Oxford, Vaxzevria)

6 COVAXIN

7 Otra opción (por favor, especifique)

8 No sé

Q9 Me administraré la dosis de refuerzo para el COVID-19 que se recomienda.

1 Sumamente de acuerdo

2 Algo de acuerdo

3 No estoy seguro(a)/sin opinión

4 Algo en desacuerdo

5 Sumamente en desacuerdo

Q10 Creo que la pandemia de COVID-19 ha terminado.

1 Sumamente de acuerdo

2 Algo de acuerdo

3 No estoy seguro(a)/sin opinión

4 Algo en desacuerdo

5 Sumamente en desacuerdo

Q11 ¿Cuánto confió en la gestión que hizo su gobierno de la pandemia de COVID-19 en su país?

1 No confié

2 No confié demasiado

3 No estoy seguro(a)

4 Confié algo

5 Confié

6 No sé o no recuerdo

Q12 ¿Afectó el desarrollo de las vacunas para el COVID-19 a su confianza en la industria farmacéutica?

1 Aumentó mi confianza

2 Sin efecto

3 Disminuyó mi confianza

4 No sé o no recuerdo

Q13 ¿Afectó el desarrollo de vacunas para el COVID-19 a su confianza en la ciencia en general?

1 Aumentó mi confianza

2 Sin efecto

3 Disminuyó mi confianza

4 No sé o no recuerdo

Q14 ¿Cuánto confía en las autoridades médicas que recomendaron que se pusiera una vacuna para el COVID-19?

1 Para nada

2 Un poco

3 Moderadamente

4 Mucho

5 No sé o no recuerdo

Q15 Durante la pandemia, ¿tuvo que aplazar o cancelar algún servicio médico que necesitaba, no relacionado con el COVID-19?

1 Sí

2 No

3 No sé o no recuerdo

Q16 ¿Seguiría una exigencia (mandato) de vacunación en el futuro de alguno de los siguientes? Seleccione todas las opciones que correspondan:

1 Gobierno

2 Empleador

3 Escuela o universidad

4 Ninguno de los anteriores

5 No sé

Q17 ¿Seguiría una exigencia (mandato) de cubrebocas en el futuro de alguno de los siguientes? Seleccione todas las opciones que correspondan:

1 Gobierno

2 Empleador

3 Escuela o universidad

4 Ninguno de los anteriores

5 No sé

Q18 En la medida que sepa, ¿tiene o ha tenido usted COVID-19?

1 Sí

2 No

3 No sé o no recuerdo

Q19 "El COVID persistente ha sido definido como "síntomas que pueden durar semanas o meses con posterioridad a la recuperación de una enfermedad grave".

En la medida que sepa, ¿tiene o ha tenido usted COVID-19 persistente? "

1 Sí

2 No

3 No sé o no recuerdo

Q20 ¿Tomó alguno de los siguientes medicamentos para tratar el COVID-19? Seleccione todas las opciones que correspondan:

1 Paxlovid

2 Molnupiravir (Lagevrio)

3 Anticuerpos monoclonales (Olumiant/Baricitinib)

4 Ivermectin

5 Medicina tradicional, extractos y tratamientos herbarios

6 No sé o no recuerdo

7 No, no tomé medicamentos para el COVID-19

Q21 ¿Ha perdido usted a un miembro de su familia o un amigo cercano debido a la enfermedad del COVID-19? Seleccione todas las opciones que correspondan:

1 Sí, durante el año pasado

2 Sí, hace más de un año

3 No

Q22 Estuve satisfecho(a) con los esfuerzos de comunicación sobre vacunas para el COVID-19 que hicieron las autoridades médicas en mi país.

1 Sumamente de acuerdo

2 Algo de acuerdo

3 No estoy seguro(a)/sin opinión/no recuerdo

4 Algo en desacuerdo

5 Sumamente en desacuerdo

Q23 Para mí es fácil saber la diferencia entre información exacta y falsa acerca de la vacuna para el COVID-19 en internet.

1 Sumamente de acuerdo

2 Algo de acuerdo

3 No estoy seguro(a)/sin opinión/no recuerdo

4 Algo en desacuerdo

5 Sumamente en desacuerdo

Q24 Sigo atento(a) a la información sobre vacunas para el COVID-19.

1 Sumamente de acuerdo

2 Algo de acuerdo

3 No estoy seguro(a)/sin opinión/no recuerdo

4 Algo en desacuerdo

5 Sumamente en desacuerdo

Q25 Sigo atento(a) a la información sobre vacunas en general.

1 Sumamente de acuerdo

2 Algo de acuerdo

3 No estoy seguro(a)/sin opinión

4 Algo en desacuerdo

5 Sumamente en desacuerdo

Q26 La pandemia de COVID-19 me ha hecho más dispuesto(a) a vacunarme contra otras enfermedades (p.ej. gripe, sarampión, hepatitis B vírica).

1 Sumamente de acuerdo

2 Algo de acuerdo

3 No estoy seguro(a)/sin opinión

4 Algo en desacuerdo

5 Sumamente en desacuerdo

Q27 Mi experiencia con la vacuna para el COVID-19 ha afectado a mi confianza en otras vacunas.

1 Aumentó mi confianza

2 Sin efecto

3 Disminuyó mi confianza

4 No sé

Q28 La cobertura de los medios sobre vacunas para el COVID-19 ha afectado a mi confianza en otras vacunas.

1 Aumentó mi confianza

2 Sin efecto

3 Disminuyó mi confianza

4 No sé

Q29 La cobertura en redes sociales sobre vacunas para el COVID-19 ha afectado a mi confianza en otras vacunas.

1 Aumentó mi confianza

2 Sin efecto

3 Disminuyó mi confianza

4 No sé

Q30 Si se aprobara una vacuna ARNm para una enfermedad de la que usted estuviera en riesgo, ¿se la pondría?

1 Definitivamente no

2 No estoy seguro(a), pero me inclino por el no

3 No estoy seguro(a), pero me inclino por el sí

4 Definitivamente sí

Q31 Si la Organización Mundial de la Salud (OMS) anunciara una nueva amenaza pandémica, ¿confiaría usted en esta información?

1 Sí

2 No

3 No sé

Q32 Si la Organización Mundial de la Salud (OMS) anunciara una nueva amenaza pandémica y aconsejara vacunarse, ¿lo haría?

1 Sí

2 No

3 No sé

Q33 "En una escala de 1 (confío completamente) y 10 (no confío para nada), por favor, indique cuánto confía en las siguientes fuentes de información sobre vacunas contra el COVID-19:

Mi familia y amigos"

Q34 Mi empleador

Q35 Mi médico o enfermero

Q36 Mi gobierno

Q37 Líderes religiosos

Q38 Medios de noticias (p.ej., televisión, internet, radio, prensa)

Q39 Redes sociales (p.ej., Facebook, Twitter [X], Instagram, WhatsApp, LinkedIn, TikTok)

Q40 Organización Mundial de la Salud (OMS)

Q41 Centros para el Control y la Prevención de Enfermedades (CMC, por sus siglas en inglés), EE.UU.

Q42 Centro Europeo para la Prevención y el Control de Enfermedades (ECDC, por sus siglas en inglés)

Q43 Las autoridades médicas públicas en mi país

Q44 ¿En qué medida está seguro(a) de que la próxima crisis de salud la gestionaremos mejor que la pandemia de COVID-19?

1 Para nada seguro(a)

2 Algo seguro(a)

3 Muy seguro(a)

4 No sé

Q45 Por lo que ha leído u oído, ¿cree que el cambio climático/calentamiento global es una amenaza real, o no es una amenaza real?

1 Amenaza real

2 No una amenaza real

Q46 ¿Cuál es su edad?

Q47 ¿Cuál es su género?

1 Masculino

2 Femenino

3 Prefiero no decir

Q48 Educación (específica por país)

1 Escuela primaria (grados 1-6)

2 Educación secundaria

3 Educación post-secundaria

4 Licenciatura

5 Grado de maestría

6 Doctorado

Q49 Nivel medio de ingreso (específico por país)

1 Sí, mi ingreso mensual es superior a 526 dólares americanos.

2 Sí, mi ingreso mensual es inferior a 526 dólares americanos.

3 No, no tengo un ingreso.

4 Rehusado/ No respondió

Q50 Regiones (específicas por país)

1 la Costa (costa)

2 Sierra (montañas)

3 Oriente (este)

4 las Islas Galápagos (oficialmente Archipiélago de Colón)

France

COVID-VAC : Une enquête mondiale sur la perception du vaccin contre la COVID-19

L'objectif de cette enquête est d'évaluer la réaction des résidents face aux mesures prises en réponse à la COVID-19. Les risques de cette enquête de routine sont considérés comme minimes. Il se peut que vous vous sentiez mal à l'aise par rapport aux répercussions de la COVID-19. Votre participation à cette étude de recherche est entièrement volontaire. Vous pouvez mettre un terme à votre participation à tout moment. Vos réponses resteront anonymes et confidentielles. Répondre à cette enquête devrait vous prendre moins de 5 minutes. En répondant aux questions, vous indiquez que vous avez lu la description de l'étude, que vous avez plus de 18 ans et que vous acceptez les conditions générales décrites.

Si vous avez des questions à propos de vos droits en tant que participant(e) à l'enquête, vous pouvez contacter le comité de protection des personnes (IRB) de l'Emerson College, s'occupant de la protection des participants volontaires aux projets de recherche. Vous pouvez contacter le chercheur principal par e-mail à l'adresse emersonpolling@emerson.edu ou le conseil de l'IRB en envoyant un e-mail à l'adresse human_subjects@emerson.edu.

Q1 "Tout d'abord, veuillez indiquer dans quelle mesure vous êtes d'accord avec cet énoncé: Le COVID-19 reste une maladie dangereuse."

1 Tout à fait d'accord

2 Plutôt d'accord

3 Pas sûr(e)/sans opinion

4 Plutôt pas d'accord

5 Pas du tout d'accord

Q2 "Maintenant, voici quelques questions sur les vaccins anti-COVID-19. Il est possible de prévenir le COVID-19 grâce à la vaccination."

1 Tout à fait d'accord

2 Plutôt d'accord

3 Pas sûr(e)/sans opinion

4 Plutôt pas d'accord

5 Pas du tout d'accord

Q3 Les risques liés au COVID-19 sont plus importants que ceux liés au vaccin.

1 Tout à fait d'accord

2 Plutôt d'accord

3 Pas sûr(e)/sans opinion

4 Plutôt pas d'accord

5 Pas du tout d'accord

Q4 Les vaccins anti-COVID-19 à ma disposition sont sûrs.

1 Tout à fait d'accord

2 Plutôt d'accord

3 Pas sûr(e)/sans opinion

4 Plutôt pas d'accord

5 Pas du tout d'accord

Q5 Je fais confiance à la science sous-jacente aux vaccins anti-COVID-19 qui sont à ma disposition.

1 Tout à fait d'accord

2 Plutôt d'accord

3 Pas sûr(e)/sans opinion

4 Plutôt pas d’accord

5 Pas du tout d’accord

Q6 Les vaccins anti-COVID-19 à ma disposition sont efficaces pour me protéger contre une forme grave du COVID-19.

1 Tout à fait d’accord

2 Plutôt d’accord

3 Pas sûr(e)/sans opinion

4 Plutôt pas d’accord

5 Pas du tout d’accord

Q7 Avez-vous reçu au moins une dose d’un vaccin anti-COVID-19?

1 Oui, j’ai reçu une dose

2 Oui, j’ai reçu au moins deux doses

3 Non

Q8 Quels vaccins anti-COVID-19 suivants avez-vous reçus? Plusieurs réponses possibles:

1 Moderna (Spikevax)

2 Pfizer (Comirnaty)

3 Johnson&Johnson (Janssen ou J&J)

4 Sinovac

5 Astra-Zeneca (Oxford, Vaxzevria)

6 COVAXIN

7 Autre (veuillez préciser)

8 Je ne sais pas

Q9 Je prendrai la dose de rappel recommandée contre le COVID-19.

1 Tout à fait d’accord

2 Plutôt d’accord

3 Pas sûr(e)/sans opinion

4 Plutôt pas d’accord

5 Pas du tout d’accord

Q10 Je pense que la pandémie de COVID-19 est terminée.

1 Tout à fait d’accord

2 Plutôt d’accord

3 Pas sûr(e)/sans opinion

4 Plutôt pas d’accord

5 Pas du tout d’accord

Q11 Dans quelle mesure aviez-vous confiance en votre gouvernement pour gérer la pandémie de COVID-19 dans votre pays?

1 Pas du tout confiance

2 Plutôt pas confiance

3 Pas sûr(e)

4 Plutôt confiance

5 Confiance

6 Ne sais pas ou ne me souviens pas

Q12 Le développement des vaccins anti-COVID-19 a-t-il affecté votre confiance dans l’industrie pharmaceutique?

1 A augmenté ma confiance

2 Aucun effet

3 A diminué ma confiance

4 Ne sais pas ou ne me souviens pas

Q13 Le développement des vaccins anti-COVID-19 a-t-il affecté votre confiance dans la science en général?

1 A augmenté ma confiance

2 Aucun effet

3 A diminué ma confiance

4 Ne sais pas ou ne me souviens pas

Q14 Dans quelle mesure accordez-vous votre confiance aux autorités sanitaires qui vous ont recommandé de faire un vaccin anti-COVID-19?

1 Pas du tout

2 Un peu

3 Modérément

4 Beaucoup

5 Ne sais pas ou ne me souviens pas

Q15 Pendant la pandémie, avez-vous dû repousser ou annuler des soins médicaux dont vous aviez besoin mais qui n'avaient aucun rapport avec le COVID-19?

1 Oui

2 Non

3 Ne sais pas ou ne me souviens pas

Q16 Suivriez-vous une exigence (obligation) de vaccination dans le futur provenant de l'une des entités suivantes? Plusieurs réponses possibles:

1 Gouvernement

2 Employeur

3 École ou université

4 Aucun de la liste

5 Je ne sais pas

Q17 Suivriez-vous une exigence (obligation) de port de masque dans le futur provenant de l'une des entités suivantes? Plusieurs réponses possibles:

1 Gouvernement

2 Employeur

3 École ou université

4 Aucun de la liste

5 Je ne sais pas

Q18 À votre connaissance, avez-vous ou avez-vous eu le COVID-19?

1 Oui

2 Non

3 Ne sais pas ou ne me souviens pas

Q19 "Le COVID long a été défini comme « des symptômes qui peuvent persister pendant des semaines ou des mois après la guérison d'une forme grave». À votre connaissance, avez-vous ou avez-vous eu un COVID long? "

1 Oui

2 Non

3 Ne sais pas ou ne me souviens pas

Q20 Avez-vous pris un des médicaments suivants pour traiter le COVID-19? Plusieurs réponses possibles:

1 Paxlovid

2 Molnupiravir (Lagevrio)

3 Anticorps monoclonaux (Olumiant/Baricitinib)

4 Ivermectine

5 Médecine traditionnelle, extraits végétaux et traitements à base de plantes

6 Ne sais pas ou ne me souviens pas

7 Non, je n'ai pas pris de médicaments pour traiter le COVID-19

Q21 Avez-vous perdu un proche (famille ou amis) à cause du COVID-19? Plusieurs réponses possibles:

1 Oui, au cours de l'année écoulée

2 Oui, il y a plus d'un an

3 Non

Q22 J'ai été satisfait(e) des efforts de communication sur les vaccins anti-COVID-19 que les autorités sanitaires ont déployé dans mon pays.

1 Tout à fait d'accord

2 Plutôt d'accord

3 Pas sûr(e)/sans opinion/ne me souviens pas

4 Plutôt pas d'accord

5 Pas du tout d'accord

Q23 Je fais facilement la différence entre les informations précises et les informations fausses qui circulent sur Internet au sujet du vaccin anti-COVID-19.

1 Tout à fait d'accord

2 Plutôt d'accord

3 Pas sûr(e)/sans opinion/ne me souviens pas

4 Plutôt pas d’accord

5 Pas du tout d’accord

Q24 Je continue de faire attention aux informations sur les vaccins anti-COVID-19.

1 Tout à fait d’accord

2 Plutôt d’accord

3 Pas sûr(e)/sans opinion/ne me souviens pas

4 Plutôt pas d’accord

5 Pas du tout d’accord

Q25 Je continue de faire attention aux informations sur les vaccins en général.

1 Tout à fait d’accord

2 Plutôt d’accord

3 Pas sûr(e)/sans opinion

4 Plutôt pas d’accord

5 Pas du tout d’accord

Q26 La pandémie de COVID-19 m’a incité à me faire vacciner contre d’autres maladies (p. ex. grippe, rougeole, hépatite virale B).

1 Tout à fait d’accord

2 Plutôt d’accord

3 Pas sûr(e)/sans opinion

4 Plutôt pas d’accord

5 Pas du tout d’accord

Q27 Mon expérience avec le vaccin anti-COVID-19 a affecté ma confiance dans les autres vaccins.

1 A augmenté ma confiance

2 Aucun effet

3 A diminué ma confiance

4 Je ne sais pas

Q28 La couverture des médias audiovisuels sur les vaccins anti-COVID-19 a affecté ma confiance dans les autres vaccins.

1 A augmenté ma confiance

2 Aucun effet

3 A diminué ma confiance

4 Je ne sais pas

Q29 La couverture des médias sociaux sur les vaccins anti-COVID-19 a affecté ma confiance dans les autres vaccins.

1 A augmenté ma confiance

2 Aucun effet

3 A diminué ma confiance

4 Je ne sais pas

Q30 Si un vaccin ARNm était approuvé pour une maladie pour laquelle vous présentiez un risque, vous feriez-vous vacciner?

1 Absolument pas

2 Pas sûr(e), mais plutôt non

3 Pas sûr(e), mais plutôt oui

4 Oui, absolument

Q31 Si l'Organisation mondiale de la santé (OMS) annonçait une nouvelle menace de pandémie, feriez-vous confiance à ces informations?

1 Oui

2 Non

3 Je ne sais pas

Q32 Si l'Organisation mondiale de la santé (OMS) annonçait une nouvelle menace de pandémie et conseillait de se faire vacciner, le feriez-vous?

1 Oui

2 Non

3 Je ne sais pas

Q33 "Sur une échelle de 1 (fait totalement confiance) à 10 (ne fait pas du tout confiance), dans quelle mesure accordez-vous votre confiance aux sources d'informations suivantes au sujet des vaccins anti-COVID-19: Ma famille et mes amis"

Q34 Mon employeur

Q35 Mon médecin ou mon infirmière

Q36 Mon gouvernement

Q37 Des chefs religieux

Q38 Les médias d'information (p. ex. télévision, Internet, radio, journaux)

Q39 Les médias sociaux (p. ex. Facebook, Twitter [X], Instagram, WhatsApp, LinkedIn, TikTok)

Q40 L'Organisation mondiale de la santé (OMS)

Q41 Les centres de prévention et de contrôle des maladies (CDC) (États-Unis)

Q42 Le centre européen de prévention et de contrôle des maladies (ECDC)

Q43 Les autorités de santé publique de mon pays

Q44 Dans quelle mesure êtes-vous convaincu(e) que la prochaine crise sanitaire sera mieux gérée que la pandémie de COVID-19?

1 Pas du tout convaincu(e)

2 Plutôt convaincu(e)

3 Vraiment convaincu(e)

4 Je ne sais pas

Q45 D’après ce que vous avez lu ou entendu, pensez-vous que le changement climatique/réchauffement de la planète est une menace réelle?

1 Une menace réelle

2 Pas une menace réelle

Q46 Quel âge avez-vous?

Q47 Êtes-vous...?

1 Un homme

2 Une femme

3 Je préfère ne pas répondre

Q48 Éducation (spécifique à chaque pays)

1 Cycle secondaire incomplet

2 Cycle secondaire complet (Baccalauréat ou équivalent)

3 Quelques années d’université ou de formation professionnelle

4 Formation professionnelle terminée (BTS, DUT ou équivalent)

5 Enseignement universitaire terminé (Bac+3)

6 Études supérieures terminées (Bac+5 : Master, diplôme d’ingénierie ou équivalent)

7 Doctorat, post-doctorat ou équivalent (Bac+8)

Q49 Niveau de revenu moyen (spécifique à chaque pays)

1 Oui, mon revenu mensuel est supérieur à 3 445 euros.

2 Oui, mon revenu mensuel est inférieur à 3 445 euros.

3 Non, je n’ai pas de revenu mensuel.

4 A refusé de répondre/N’a pas répondu

Q50 Régions (spécifiques à chaque pays)

1 Nord de la France : Normandie, Hauts-de-France, Ile-de-France.

2 Sud de la France : Provence-Alpes-Côte d’Azur, Occitanie, Corse.

3 Centre de la France : Centre-Val-de-Loire.

4 Est de la France : Grand Est, Auvergne-Rhône-Alpes, Bourgogne-Franche-Comté

5 Ouest de la France : Pays de La Loire, Bretagne, Nouvelle-Aquitaine (Aquitaine, Poitou-Charentes, Limousin)

GERMANY

COVID-Impfstoff: Eine weltweite Umfrage zur Wahrnehmung des COVID-19-Impfstoffs

Der Zweck der Umfrage ist es, die Wahrnehmung der Bevölkerung auf die Maßnahmen gegen COVID-19 zu erfassen. Die Risiken bei dieser Routinebefragung werden als minimal angesehen. Möglicherweise empfinden Sie es als unangenehm, über die Auswirkungen von COVID-19 nachzudenken. Die Teilnahme an dieser Studie ist völlig freiwillig und Sie können die Teilnahme jederzeit beenden. Ihre Antworten werden anonym und vertraulich behandelt. Die Umfrage dauert ungefähr 5 Minuten. Durch die Beantwortung der Fragen bestätigen Sie, dass Sie die Beschreibung der Studie gelesen haben, dass Sie über 18 Jahre alt sind und dass Sie mit den aufgeführten Bedingungen einverstanden sind.

Wenn Sie Fragen zu Ihren Rechten als Teilnehmer an Forschungsprojekten haben, können Sie sich an das Institutional Review Board (IRB) des Emerson College wenden, das sich mit den Rechten von Freiwilligen in Forschungsprojekten befasst. Sie können den Leiter der Studie per E-Mail unter emersonpolling@emerson.edu erreichen oder den Vorsitzenden des IRB per E-Mail unter human_subjects@emerson.edu.

Q1 "Zuallererst, geben Sie bitte an, inwieweit Sie dieser Aussage zustimmen: COVID-19 bleibt eine gefährliche Gesundheitsbedrohung."

1 Stimme vollkommen zu

2 Stimme einigermaßen zu

3 Unsicher/keine Meinung

4 Stimme eher nicht zu

5 Stimme überhaupt nicht zu

Q2 "Es folgen einige Fragen zu COVID-19-Impfungen. COVID-19 kann durch eine Impfung verhindert werden."

1 Stimme vollkommen zu

2 Stimme einigermaßen zu

3 Unsicher/keine Meinung

4 Stimme eher nicht zu

5 Stimme überhaupt nicht zu

Q3 Die Risiken einer COVID-19-Erkrankung sind größer als die Risiken der Impfung.

1 Stimme vollkommen zu

2 Stimme einigermaßen zu

3 Unsicher/keine Meinung

4 Stimme eher nicht zu

5 Stimme überhaupt nicht zu

Q4 Die COVID-19-Impfstoffe, die mir zur Verfügung stehen, sind sicher.

1 Stimme vollkommen zu

2 Stimme einigermaßen zu

3 Unsicher/keine Meinung

4 Stimme eher nicht zu

5 Stimme überhaupt nicht zu

Q5 Ich vertraue der Wissenschaft hinter den mir zur Verfügung stehenden COVID-19-Impfstoffen.

1 Stimme vollkommen zu

2 Stimme einigermaßen zu

3 Unsicher/keine Meinung

4 Stimme eher nicht zu

5 Stimme überhaupt nicht zu

Q6 Die mir zur Verfügung stehenden COVID-19-Impfstoffe schützen wirksam vor einem schweren Verlauf.

1 Stimme vollkommen zu

2 Stimme einigermaßen zu

3 Unsicher/keine Meinung

4 Stimme eher nicht zu

5 Stimme überhaupt nicht zu

Q7 Haben Sie mindestens eine Dosis eines COVID-19-Impfstoffs verabreicht bekommen?

1 Ja, ich habe eine Dosis verabreicht bekommen

2 Ja, ich habe zwei oder mehr Dosen verabreicht bekommen

3 Nein

Q8 Welche der folgenden COVID-19-Impfstoffe haben Sie verabreicht bekommen? Wählen Sie alle zutreffenden Optionen aus:

1 Moderna (Spikevax)

2 Pfizer (Comirnaty)

3 Johnson & Johnson (Janssen oder J&J)

4 Sinovac

5 Astra-Zeneca (Oxford, Vaxzevria)

6 COVAXIN

7 Sonstige (bitte angeben)

8 Weiß ich nicht

Q9 Ich werde mir die empfohlene COVID-19-Auffrischungsimpfung verabreichen lassen.

1 Stimme vollkommen zu

2 Stimme einigermaßen zu

3 Unsicher/keine Meinung

4 Stimme eher nicht zu

5 Stimme überhaupt nicht zu

Q10 Ich glaube, die COVID-19-Pandemie ist vorbei.

1 Stimme vollkommen zu

2 Stimme einigermaßen zu

3 Unsicher/keine Meinung

4 Stimme eher nicht zu

5 Stimme überhaupt nicht zu

Q11 Wie viel Vertrauen hatten Sie in Ihre Regierung, was den Umgang mit der COVID-19-Pandemie in Ihrem Land betrifft?

1 Überhaupt kein Vertrauen

2 Kein wirkliches Vertrauen

3 Unsicher

4 Wenig Vertrauen

5 Großes Vertrauen

6 Ich weiß nicht oder erinnere mich nicht

Q12 Hat die Herstellung der COVID-19-Impfstoffe Ihr Vertrauen in die Pharmaindustrie geschmälert?

1 Vertrauen erhöht

2 Keine Auswirkung

3 Vertrauen geschmälert

4 Ich weiß nicht oder erinnere mich nicht

Q13 Hat die Herstellung der COVID-19-Impfstoffe Ihr Vertrauen in die Wissenschaft im Allgemeinen beeinflusst?

1 Vertrauen erhöht

2 Keine Auswirkung

3 Vertrauen geschmälert

4 Ich weiß nicht oder erinnere mich nicht

Q14 Wie sehr vertrauen Sie den Gesundheitsbehörden, die Ihnen eine COVID-19-Impfung empfohlen haben?

1 Überhaupt nicht

2 Ein wenig

3 Ziemlich

4 Sehr stark

5 Ich weiß nicht oder erinnere mich nicht

Q15 Mussten Sie während der Pandemie medizinische Behandlungen verschieben oder absagen, die notwendig waren und nicht mit COVID-19 zusammenhingen?

1 Ja

2 Nein

3 Ich weiß nicht oder erinnere mich nicht

Q16 Würden Sie in Zukunft einer Impfpflicht (Mandat) von einem der folgenden Akteure nachkommen? Wählen Sie alle zutreffenden Optionen aus:

1 Regierung

2 Arbeitgeber

3 Schule oder Universität

4 Keine der genannten

5 Weiß ich nicht

Q17 Würden Sie in Zukunft einer Maskenpflicht (Mandat) für Mund und Nase von einem der folgenden Akteure nachkommen? Wählen Sie alle zutreffenden Optionen aus:

1 Regierung

2 Arbeitgeber

3 Schule oder Universität

4 Keine der genannten

5 Weiß ich nicht

Q18 Waren oder sind Sie Ihrer Kenntnis nach an COVID-19 erkrankt?

1 Ja

2 Nein

3 Ich weiß nicht oder erinnere mich nicht

Q19 "Long-COVID wurde definiert als „Symptome, die noch Wochen oder Monate nach der Genesung von einer akuten Erkrankung fortbestehen können“. Waren oder sind Sie Ihrer Kenntnis nach an Long-COVID erkrankt? "

1 Ja

2 Nein

3 Ich weiß nicht oder erinnere mich nicht

Q20 Haben Sie eines der folgenden Medikamente zur Behandlung von COVID-19 eingenommen? Wählen Sie alle zutreffenden Optionen aus:

1 Paxlovid

2 "Molnupiravir (Lagevrio)"

3 Monoklonale Antikörper (Olumiant/Baricitinib)

4 Ivermectin

5 Traditionelle Medizin, Kräuterextrakte und pflanzliche Heilmittel

6 Ich weiß nicht oder erinnere mich nicht

7 Nein, ich habe keine Medikamente gegen COVID-19 eingenommen

Q21 Haben Sie einen Familienangehörigen oder engen Freund durch eine COVID-19-Erkrankung verloren? Wählen Sie alle zutreffenden Optionen aus:

1 Ja, innerhalb des letzten Jahres

2 Ja, vor mehr als einem Jahr

3 Nein

Q22 Ich war zufrieden mit den Bemühungen der Gesundheitsbehörden in meinem Land, über die COVID-19-Impfung zu informieren.

1 Stimme vollkommen zu

2 Stimme einigermaßen zu

3 Unsicher/keine Meinung/erinnere mich nicht

4 Stimme eher nicht zu

5 Stimme überhaupt nicht zu

Q23 Es fällt mir leicht, den Unterschied zwischen richtigen und falschen Informationen über die COVID-19-Impfung im Internet zu erkennen.

1 Stimme vollkommen zu

2 Stimme einigermaßen zu

3 Unsicher/keine Meinung/erinnere mich nicht

4 Stimme eher nicht zu

5 Stimme überhaupt nicht zu

Q24 Ich verfolge weiterhin aufmerksam die Informationen über COVID-19-Impfungen.

1 Stimme vollkommen zu

2 Stimme einigermaßen zu

3 Unsicher/keine Meinung/erinnere mich nicht

4 Stimme eher nicht zu

5 Stimme überhaupt nicht zu

Q25 Ich achte weiterhin auf Informationen über Impfungen im Allgemeinen.

1 Stimme vollkommen zu

2 Stimme einigermaßen zu

3 Unsicher/keine Meinung

4 Stimme eher nicht zu

5 Stimme überhaupt nicht zu

Q26 Die COVID-19-Pandemie hat meine Bereitschaft erhöht, mich gegen andere Krankheiten (z. B. Grippe, Masern, Virushepatitis B) impfen zu lassen.

1 Stimme vollkommen zu

2 Stimme einigermaßen zu

3 Unsicher/keine Meinung

4 Stimme eher nicht zu

5 Stimme überhaupt nicht zu

Q27 Meine Erfahrung mit der COVID-19-Impfung hat mein Vertrauen in andere Impfungen beeinflusst.

1 Vertrauen erhöht

2 Keine Auswirkung

3 Vertrauen geschmälert

4 Weiß ich nicht

Q28 Die Medienberichterstattung über die COVID-19-Impfung hat mein Vertrauen in andere Impfungen beeinflusst.

1 Vertrauen erhöht

2 Keine Auswirkung

3 Vertrauen geschmälert

4 Weiß ich nicht

Q29 Die Berichterstattung in den sozialen Medien über die COVID-19-Impfung hat mein Vertrauen in andere Impfungen beeinflusst.

1 Vertrauen erhöht

2 Keine Auswirkung

3 Vertrauen geschmälert

4 Weiß ich nicht

Q30 Wenn eine mRNA-Impfung für eine Erkrankung zugelassen wird, zu deren Gefahrengruppe Sie gehören, würden Sie sich damit impfen lassen?

1 Definitiv nicht

2 Unsicher, aber tendenziell nein

3 Unsicher, aber tendenziell ja

4 Eindeutig ja

Q31 Wenn die Weltgesundheitsorganisation (WHO) eine neue Pandemie ankündigt, würden Sie dieser Information vertrauen?

1 Ja

2 Nein

3 Weiß ich nicht

Q32 Wenn die Weltgesundheitsorganisation (WHO) eine neue Pandemie ankündigt und zur Impfung rät, würden Sie sich impfen lassen?

1 Ja

2 Nein

3 Weiß ich nicht

Q33 "Auf einer Skala von 1 (volles Vertrauen) bis 10 (überhaupt kein Vertrauen), wie sehr vertrauen Sie den folgenden Informationsquellen über COVID-19-Impfungen: Meine Familie und Freunde"

Q34 Mein Arbeitgeber

Q35 Mein Arzt oder Krankenpfleger

Q36 Meine Regierung

Q37 Religiöse Anführer

Q38 Nachrichten-Medien (z. B. Fernsehen, Internet, Radio, Zeitungen)

Q39 Soziale Medien (z. B. Facebook, Twitter [X], Instagram, WhatsApp, LinkedIn, TikTok)

Q40 Weltgesundheitsorganisation (WHO)

Q41 Centers for Disease Control and Prevention (CDC), USA

Q42 Europäisches Zentrum für die Prävention und die Kontrolle von Krankheiten (ECDC)

Q43 Gesundheitsbehörden in meinem Land

Q44 Wie zuversichtlich sind Sie, dass wir die nächste medizinische Krise besser bewältigen werden als die COVID-19-Pandemie?

1 Überhaupt nicht zuversichtlich

2 Einigermaßen zuversichtlich

3 Sehr zuversichtlich

4 Weiß ich nicht

Q45 Glauben Sie nach dem, was Sie gelesen oder gehört haben, dass der Klimawandel/die globale Erwärmung eine echte Bedrohung ist oder nicht?

1 Echte Bedrohung

2 Keine echte Bedrohung

Q46 Wie alt sind Sie?

Q47 Bitte geben Sie Ihr Geschlecht an:

1 Männlich

2 Weiblich

3 Keine Angabe

Q48 Ausbildung (länderspezifisch)

1 Noch in der Schule

2 Realschulabschluss / mittlere Reife

3 Abitur / Hochschulreife

4 Diplom einer Berufsakademie / Berufsausbildung

5 Bachelor / Fachhochschulabschluss

6 Master / Dr. / PhD.

Q49 Mittleres Einkommensniveau (länderspezifisch)

1 Ja, mein monatliches Einkommen beträgt mehr als 4.011 Euro.

2 Ja, mein monatliches Einkommen beträgt weniger als 4.011 Euro.

3 Nein, ich habe kein Einkommen.

4 Antwort verweigert/ Nicht geantwortet

Q50 Regionen (länderspezifisch)

1 Nördliches Tiefland oder Norddeutsche Ebene

2 das zentrale Hochland

3 die Alpen

Ghana

COVID-VAC: A global survey of COVID-19 vaccine perceptions

The purpose of the survey is to measure resident's reactions to COVID-19 response efforts. The risks in this routine survey are considered minimal. You may feel uncomfortable considering the implications of COVID-19 and participation in this research study is completely voluntary and you may discontinue participation at any time. Your responses will remain anonymous and confidential. The survey should take less than 5 minutes to complete. By answering the questions, you are indicating that you have read the description of the study, are over the age of 18, and that you agree to the terms as described.

If you have questions about your rights as a research participant, you may contact the Emerson College Institutional Review Board (IRB), which is concerned with the protection of volunteers in research projects. You may reach the lead investigator via email at emersonpolling@emerson.edu or the Chair of the IRB by e-mailing human_subjects@emerson.edu.

Q1 "First, please indicate your level of agreement with this statement: COVID-19 remains a dangerous health threat."

1 Strongly agree

2 Somewhat agree

3 Unsure/no opinion

4 Somewhat disagree

5 Strongly disagree

Q2 "Now, here are some questions related to COVID-19 vaccines. COVID-19 can be prevented by vaccination."

1 Strongly agree

2 Somewhat agree

3 Unsure/no opinion

4 Somewhat disagree

5 Strongly disagree

Q3 The risks of COVID-19 disease are greater than the risks of the vaccine.

1 Strongly agree

2 Somewhat agree

3 Unsure/no opinion

4 Somewhat disagree

5 Strongly disagree

Q4 The COVID-19 vaccines available to me are safe.

1 Strongly agree

2 Somewhat agree

3 Unsure/no opinion

4 Somewhat disagree

5 Strongly disagree

Q5 I trust the science behind the COVID-19 vaccines available to me.

1 Strongly agree

2 Somewhat agree

3 Unsure/no opinion

4 Somewhat disagree

5 Strongly disagree

Q6 The COVID-19 vaccines available to me are effective in protecting against severe COVID-19.

1 Strongly agree

2 Somewhat agree

3 Unsure/no opinion

4 Somewhat disagree

5 Strongly disagree

Q7 Have you received at least one dose of a COVID-19 vaccine?

1 Yes, I received one dose

2 Yes, I received two or more doses

3 No

Q8 Which of the following COVID-19 vaccines did you receive? Select all that apply:

1 Moderna (Spikevax)

2 Pfizer (Comirnaty)

3 Johnson&Johnson (Janssen or J&J)

4 Sinovac

5 Astra-Zeneca (Oxford, Vaxzevria)

6 COVAXIN

7 Other (please specify)

8 Don’t know

Q9 I will take the recommended COVID-19 booster.

1 Strongly agree

2 Somewhat agree

3 Unsure/no opinion

4 Somewhat disagree

5 Strongly disagree

Q10 I believe the COVID-19 pandemic is over.

1 Strongly agree

2 Somewhat agree

3 Unsure/no opinion

4 Somewhat disagree

5 Strongly disagree

Q11 How much did you trust your government's management of the COVID-19 pandemic in your country?

1 Did not trust

2 Somewhat did not trust

3 Unsure

4 Somewhat trust

5 Trust

6 Don't know or don't remember

Q12 Did the development of the COVID-19 vaccines affect your trust in the pharmaceutical industry?

1 Increased trust

2 No effect

3 Decreased trust

4 Don't know or don't remember

Q13 Did the development of COVID-19 vaccines affect your trust in science generally?

1 Increased trust

2 No effect

3 Decreased trust

4 Don't know or don't remember

Q14 How much do you trust the health authorities that recommended you get a COVID-19 vaccine?

1 Not at all

2 A little

3 Moderately

4 Very much

5 Don’t know or don’t remember

Q15 During the pandemic, did you have to delay or cancel any medical services that you needed unrelated to COVID-19?

1 Yes

2 No

3 Don’t know or don’t remember

Q16 Would you follow a vaccine requirement (mandate) in the future from any of the following? Select all that apply:

1 Government

2 Employer

3 School or university

4 None of the above

5 Don’t know

Q17 Would you follow a face mask requirement (mandate) in the future from any of the following? Select all that apply:

1 Government

2 Employer

3 School or university

4 None of the above

5 Don’t know

Q18 To your knowledge, do you have or have you had COVID-19?

1 Yes

2 No

3 Don’t know or don’t remember

Q19 "Long-COVID has been defined as 'symptoms that can last for weeks or months after recovery from acute illness'. To your knowledge, do you have or have you had Long COVID? "

1 Yes

2 No

3 Don’t know or don’t remember

Q20 Did you take any of the following medicines to treat COVID-19? Select all that apply:

1 Paxlovid

2 Molnupiravir (Lagevrio)

3 Monoclonal antibodies (Olumiant/Baricitinib)

4 Ivermectin

5 Traditional medicine, herbal extracts and treatments

6 Don’t know or don’t remember

7 No, I did not take medicines for COVID-19

Q21 Have you lost a family member or close friend to COVID-19 disease? Select all that apply:

1 Yes, within the past year

2 Yes, more than a year ago

3 No

Q22 I was satisfied with the communication efforts on COVID-19 vaccines made by health authorities in my country.

1 Strongly agree

2 Somewhat agree

3 Unsure/no opinion/don’t remember

4 Somewhat disagree

5 Strongly disagree

Q23 It is easy for me to know the difference between accurate and false information about the COVID-19 vaccine on the Internet.

1 Strongly agree

2 Somewhat agree

3 Unsure/no opinion/don’t remember

4 Somewhat disagree

5 Strongly disagree

Q24 I continue to pay attention to information on COVID-19 vaccines.

1 Strongly agree

2 Somewhat agree

3 Unsure/no opinion/don’t remember

4 Somewhat disagree

5 Strongly disagree

Q25 I continue to pay attention to information on vaccines in general.

1 Strongly agree

2 Somewhat agree

3 Unsure/no opinion

4 Somewhat disagree

5 Strongly disagree

Q26 The COVID-19 pandemic has made me more willing to get vaccinated against other diseases (e.g., flu, measles, viral hepatitis B).

1 Strongly agree

2 Somewhat agree

3 Unsure/no opinion

4 Somewhat disagree

5 Strongly disagree

Q27 My experience with the COVID-19 vaccine has affected my trust in other vaccines.

1 Increased trust

2 No effect

3 Decreased trust

4 Don’t know

Q28 Broadcast media coverage on COVID-19 vaccines has affected my trust in other vaccines.

1 Increased trust

2 No effect

3 Decreased trust

4 Don’t know

Q29 Social media coverage on COVID-19 vaccines has affected my trust in other vaccines.

1 Increased trust

2 No effect

3 Decreased trust

4 Don’t know

Q30 If an mRNA vaccine is approved for a disease for which you are at risk, would you take it?

1 Definitely no

2 Unsure, but leaning towards no

3 Unsure, but leaning towards yes

4 Definitely yes

Q31 If the World Health Organization (WHO) announced a new pandemic threat, would you trust this information?

1 Yes

2 No

3 Don’t know

Q32 If the World Health Organization (WHO) announced a new pandemic threat and advised getting vaccinated, would you?

1 Yes

2 No

3 Don’t know

Q33 "On a scale of 1 (trust completely) to 10 (do not trust at all) how much do you trust the following sources of information about COVID-19 vaccines: My family and friends"

Q34 My employer

Q35 My doctor or nurse

Q36 My government

Q37 Religious leaders

Q38 News media (e.g., television, Internet, radio, newspapers)

Q39 Social media (e.g., Facebook, Twitter [X], Instagram, WhatsApp, LinkedIn, TikTok)

Q40 World Health Organization (WHO)

Q41 Centers for Disease Control and Prevention (CDC), USA

Q42 European Centre for Disease Prevention and Control (ECDC)

Q43 The public health authorities in my country

Q44 How confident are you that we will manage the next health crisis better than the COVID-19 pandemic?

1 Not at all confident

2 Somewhat confident

3 Very confident

4 Don’t know

Q45 From what you have read or heard, do you think climate change/global warming is a real threat or not a real threat?

1 Real threat

2 Not a real threat

Q46 What is your age?

Q47 What is your gender?

1 Male

2 Female

3 Prefer not to say

Q48 Education (country specific)

1 Primary school

2 Junior Secondary/High School

3 Senior Secondary School

4 University Bachleor's Degree

5 Postgraduate Education Completed

6 Doctorate, Post-doctorate or equivalent Completed

Q49 Median Income Level (country specific)

1 Yes, my monthly income is more than 2,263 Ghanaian Cedi.

2 Yes, my monthly income is less than 2,263 Ghanaian Cedi.

3 No, I do not have an income.

4 Refused/ Did not answer

Q50 Regions (country specific)

1 Ashanti

2 Greater Accra

3 Eastern

4 Northern

5 Western

6 Bono

7 Volta

8 Central

9 Upper East

10 Upper West

11 Oti

12 Bono East

13 Ahafo

14 Savannah

15 Western North

16 North East

India

शीर्षक: COVID-VAC: COVID-19 वैक्सीन सम्बन्धी दृष्टिकोणों का वैश्विक सर्वेक्षण

इस सर्वेक्षण का उद्देश्य, COVID-19 सम्बन्धी जवाब देने की कोशिशों के प्रति निवासियों की प्रतिक्रियाओं का मूल्यांकन करना है. इस नियमित सर्वेक्षण के जोखिमों को न्यूनतम माना गया है. हो सकता है कि आपको COVID-19 के परिणामों पर विचार करके असहज महसूस हो और इस शोध अध्ययन में प्रतिभागिता पूरी तरह से स्वैच्छिक है और आप इसमें प्रतिभागिता को किसी भी समय समाप्त कर सकते हैं. आपके जवाब पूरी तरह अनाम और गोपनीय रहेंगे। यह सर्वेक्षण पूरा करने में लगभग 5 मिनट से भी कम समय लगेगा. सवालों के जवाब देकर, आप यह इंगित कर रहे हैं कि आपने अध्ययन के विवरण को पढ़ लिया है, आपकी उम्र 18 वर्ष से अधिक है और यह कि आप बताई गई शर्तों से सहमत हैं.

अगर आप अनुसंधान के प्रतिभागी के तौर पर अपने अधिकारों के बारे में कोई सवाल पूछना चाहते हैं, तो आप Emerson College के संस्थागत समीक्षा बोर्ड (IRB) से संपर्क कर सकते हैं, जो शोध परियोजना में वॉलंटियर की सुरक्षा से संबंधित है. आप जांचकर्ता से ईमल के ज़रिए emersonpolling@emerson.edu पर या IRB के अध्यक्ष से human_subjects@emerson.edu पर ईमेल करके संपर्क कर सकते हैं.

प्र 1 "सबसे पहले, कृपया इंगित करें कि नीचे दिए गए कथन से आप किस स्तर तक सहमत हैं: COVID-19 स्वास्थ्य के लिए अभी भी गंभीर खतरा बना हुआ है।"

1 पूरी तरह से सहमत

2 कुछ हद तक सहमत

3 पक्का नहीं कह सकते/कोई राय नहीं देंगे

4 कुछ हद तक असहमत हैं

5 पूरी तरह से असहमत

प्र2 "अब, यहां COVID-19 वैक्सीन से संबंधित कुछ सवाल दिए गए हैं। COVID-19 को वैक्सीन लगवा कर रोका जा सकता है।"

1 पूरी तरह से सहमत

2 कुछ हद तक सहमत

3 पक्का नहीं कह सकते/कोई राय नहीं देंगे

4 कुछ हद तक असहमत हैं

5 पूरी तरह से असहमत

प्र3 COVID-19 बीमारी का जोखिम, वैक्सीन के जोखिमों से ज़्यादा है।

1 पूरी तरह से सहमत

2 कुछ हद तक सहमत

3 पक्का नहीं कह सकते/कोई राय नहीं देंगे

4 कुछ हद तक असहमत हैं

5 पूरी तरह से असहमत

प्र4 मुझे COVID-19 के जो वैक्सीन उपलब्ध हैं, वे सुरक्षित हैं।

1 पूरी तरह से सहमत

2 कुछ हद तक सहमत

3 पक्का नहीं कह सकते/कोई राय नहीं देंगे

4 कुछ हद तक असहमत हैं

5 पूरी तरह से असहमत

प्र5 मुझे अपने लिए उपलब्ध COVID-19 वैक्सीन की वैज्ञानिकता पर भरोसा है।

1 पूरी तरह से सहमत

2 कुछ हद तक सहमत

3 पक्का नहीं कह सकते/कोई राय नहीं देंगे

4 कुछ हद तक असहमत हैं

5 पूरी तरह से असहमत

प्र6 मेरे लिए उपलब्ध COVID-19 वैक्सीन, मुझे COVID-19 की गंभीर बीमारी से सुरक्षा देने में प्रभावी है।

1 पूरी तरह से सहमत

2 कुछ हद तक सहमत

3 पक्का नहीं कह सकते/कोई राय नहीं देंगे

4 कुछ हद तक असहमत हैं

5 पूरी तरह से असहमत

प्र7 क्या आपको COVID-19 वैक्सीन की कम-से-कम एक खुराक मिल गई है?

1 हां, मुझे एक खुराक मिल गई है।

2 हां, मुझे दो या इससे ज़्यादा खुराक मिल गई हैं।

3 नहीं

प्र8 आपको COVID-19 के वैक्सीन में से कौन सा वैक्सीन मिला है? वे सभी विकल्प चुनें, जो लागू होते हैं:

1 Moderna (Spikevax)

2 Pfizer (Comirnaty)

3 Johnson&Johnson (Janssen या J&J)

4 Sinovac

5 Astra-Zeneca (Oxford, Vaxzevria)

6 COVAXIN

7 अन्य (कृपया बताएं)

8 पता नहीं

प्र9 मैं सुझाया गया COVID-19 बूस्टर लूंगा/लूंगी।

1 पूरी तरह से सहमत

2 कुछ हद तक सहमत

3 पक्का नहीं कह सकते/कोई राय नहीं देंगे

4 कुछ हद तक असहमत हैं

5 पूरी तरह से असहमत

प्र10 मुझे लगता है कि COVID-19 महामारी खत्म हो गई है।

1 पूरी तरह से सहमत

2 कुछ हद तक सहमत

3 पक्का नहीं कह सकते/कोई राय नहीं देंगे

4 कुछ हद तक असहमत हैं

5 पूरी तरह से असहमत

प्र11 आपको अपने देश में COVID-19 महामारी के अपनी सरकार के प्रबंधन पर कितना भरोसा था?

1 भरोसा नहीं था

2 कुछ हद तक भरोसा नहीं था

3 पक्का नहीं कह सकते

4 कुछ हद तक भरोसा था

5 भरोसा था

6 पता नहीं या याद नहीं आ रहा

प्र12 क्या COVID-19 वैक्सीन बढ़ने से फ़ार्मास्यूटिकल उद्योग को लेकर आपके भरोसे पर प्रभाव डाला?

1 भरोसा बढ़ा

2 कोई असर नहीं

3 भरोसा कम हुआ

4 पता नहीं या याद नहीं आ रहा

प्र13 क्या COVID-19 वैक्सीन बढ़ने से विज्ञान में आपके भरोसे पर प्रभाव डाला?

1 भरोसा बढ़ा

2 कोई असर नहीं

3 भरोसा कम हुआ

4 पता नहीं या याद नहीं आ रहा

प्र14 आप उन स्वास्थ्य प्राधिकारियों पर कितना भरोसा करते/करती हैं , जिन्होंने आपको COVID-19 वैक्सीन लेने का सुझाव दिया था?

1 बिल्कुल नहीं

2 थोड़ा-बहुत

3 ठीक ठाक

4 बहुत अधिक

5 पता नहीं या याद नहीं आ रहा

प्र15 महामारी के दौरान, क्या आपको अपने लिए ज़रूरी किसी ऐसी चिकित्सा सेवा में देरी या उसे कैंसिल करना पड़ा, जो COVID-19 से संबंधित नहीं थी?

1 हां

2 नहीं

3 पता नहीं या याद नहीं आ रहा

प्र16 क्या आप इनमें से किसी की ओर से भी भविष्य में वैक्सीन की मांग (अनुदेश) का अनुसरण करेंगे? वे सभी विकल्प चुनें, जो लागू होते हैं:

1 सरकारी

2 नियोक्ता

3 स्कूल या यूनिवर्सिटी

4 इनमें से कोई नहीं

5 पता नहीं

प्र.17 क्या आप इनमें से किसी की ओर से भी भविष्य में फ़ेसमास्क की मांग (अनुदेश) का अनुसरण करेंगे? वे सभी विकल्प चुनें, जो लागू होते हैं:

1 सरकारी

2 नियोक्ता

3 स्कूल या यूनिवर्सिटी

4 इनमें से कोई नहीं

5 पता नहीं

प्र18 आपकी जानकारी में, क्या आपको COVID-19 है या हुआ था?

1 हां

2 नहीं

3 पता नहीं या याद नहीं आ रहा

प्र19 "लंबे समय तक चलने वाली COVID को "तीव्र बीमारी से रिकवरी के बाद कई हफ़्तों या कई महीनों तक चलने वाले लक्षणों" के रूप में परिभाषित किया गया है। आपकी जानकारी में, क्या आपको लंबे समय तक चलने वाला COVID है या हुआ था? "

1 हां

2 नहीं

3 पता नहीं या याद नहीं आ रहा

प्र20 क्या COVID-19 का उपचार करने के लिए आपने इनमें से कोई भी दवा ली थी? वे सभी विकल्प चुनें, जो लागू होते हैं:

1 Paxlovid

2 Molnupiravir (Lagevrio)

3 Monoclonal antibodies (Olumiant/Baricitinib)

4 Ivermectin

5 पारंपरिक दवा, हर्बल एक्सट्रैक्ट और उपचार

6 पता नहीं या याद नहीं आ रहा

7 नहीं, मैंने COVID-19 के लिए दवाएं नहीं ली थीं

प्र21 क्या आपने COVID-19 बीमारी की वजह से अपने परिवार के किसी सदस्य या करीबी दोस्त को खो दिया है? वे सभी विकल्प चुनें, जो लागू होते हैं:

1 हां, पिछले एक वर्ष के अंदर

2 हां, एक वर्ष से अधिक समय पहले

3 नहीं

प्र22 मैं अपने देश में स्वास्थ्य प्राधिकरण द्वारा COVID-19 वैक्सीन के संबंध में संचार की कोशिशों से संतुष्ट था.

1 पूरी तरह से सहमत

2 कुछ हद तक सहमत

3 पक्का नहीं कह सकते/कोई राय नहीं/याद नहीं है

4 कुछ हद तक असहमत हैं

5 पूरी तरह से असहमत

प्र23 इंटरनेट पर COVID-19 वैक्सीन के बारे में सही और गलत जानकारी के बीच अंतर को पहचानना मेरे लिए आसान है.

1 पूरी तरह से सहमत

2 कुछ हद तक सहमत

3 पक्का नहीं कह सकते/कोई राय नहीं/याद नहीं है

4 कुछ हद तक असहमत हैं

5 पूरी तरह से असहमत

प्र24 मैं COVID-19 वैक्सीनों के बारे में जानकारी पर अभी भी ध्यान देता हूं.

1 पूरी तरह से सहमत

2 कुछ हद तक सहमत

3 पक्का नहीं कह सकते/कोई राय नहीं/याद नहीं है

4 कुछ हद तक असहमत हैं

5 पूरी तरह से असहमत

प्र25 मैं वैक्सीनों के बारे में जानकारी पर अभी भी लगातार ध्यान देता हूं।

1 पूरी तरह से सहमत

2 कुछ हद तक सहमत

3 पक्का नहीं कह सकते/कोई राय नहीं देंगे

4 कुछ हद तक असहमत हैं

5 पूरी तरह से असहमत

**प्र26	COVID-19 महामारी ने मुझे अन्य बीमारियों (उदा. फ़्लू, मीज़ल्स, वायरल हेपिटाइटिस B) की तुलना में वैक्सीन लगवाने के लिए ज़्यादा इच्छुक बना दिया है।**

1	पूरी तरह से सहमत

2	कुछ हद तक सहमत

3	पक्का नहीं कह सकते/कोई राय नहीं देंगे

4	कुछ हद तक असहमत हैं

5	पूरी तरह से असहमत

**प्र27	COVID-19 वैक्सीन के बारे में मेरे अनुभव ने अन्य वैक्सीन पर मेरे भरोसे को प्रभावित किया है।**

1	भरोसा बढ़ा

2	कोई असर नहीं

3	भरोसा कम हुआ

4	पता नहीं

**प्र28	COVID-19 वैक्सीन के बारे में प्रसारण मीडिया कवरेज ने अन्य वैक्सीन पर मेरे भरोसे को प्रभावित किया है।**

1	भरोसा बढ़ा

2	कोई असर नहीं

3	भरोसा कम हुआ

4	पता नहीं

**प्र29	COVID-19 वैक्सीन के बारे में सोशल मीडिया कवरेज ने अन्य वैक्सीन में मेरे भरोसे को प्रभावित किया है।**

1	भरोसा बढ़ा

2	कोई असर नहीं

3	भरोसा कम हुआ

4	पता नहीं

**प्र30	अगर किसी ऐसी बीमारी के बारे में, जिसका आपको खतरा है, mRNA वैक्सीन स्वीकृत हो, तो क्या आप उसे लेना चाहेंगे?**

1	बिल्कुल नहीं

2 पक्का नहीं कह सकता/सकती, लेकिन मेरा झुकाव उसे न लेने की ओर है

3 पक्का नहीं कह सकता/सकती, लेकिन मेरा झुकाव उसे लेने की ओर है

4 बिल्कुल हां

प्र31 अगर विश्व स्वास्थ्य संगठन (WHO) ने किसी नई महामारी के खतरे की घोषणा की, तो क्या आप इस जानकारी पर भरोसा करेंगे?

1 हां

2 नहीं

3 पता नहीं

प्र32 अगर विश्व स्वास्थ्य संगठन (WHO) ने किसी नई महामारी के खतरे की घोषणा की, और वैक्सीन लगवाने का सुझाव दिया तो क्या आप वैक्सीन लगवाएंगे?

1 हां

2 नहीं

3 पता नहीं

प्र33 "1 (पूरी तरह भरोसा करेंगे) से लेकर 10 (बिल्कुल भी भरोसा नहीं करेंगे) के पैमाने पर आप जानकारी के इन स्रोतों के बारे में कितना भरोसा करते हैं COVID-19 वैक्सीन: मेरे परिवार के लोग और दोस्त"

प्र34 मेरा नियोक्ता

प्र35 मेरा डॉक्टर या नर्स

प्र36 मेरी सरकार

प्र37 धार्मिक नेतृत्व

प्र38 समाचार मीडिया (उदा. टेलीविज़न, इंटरनेट, रेडियो, समाचार-पत्र)

प्र39 सोशल मीडिया (उदा., Facebook, Twitter [X], Instagram, WhatsApp, LinkedIn, TikTok)

प्र40 विश्व स्वास्थ्य संगठन (WHO)

प्र41 सेंटर फ़ॉर डिसीज़ कंट्रोल एंड प्रिवेंशन (CDC), अमेरिका

प्र42 यूरोपियन सेंटर फ़ॉर डिसीज़ प्रिवेंशन एंड कंट्रोल (ECDC)

प्र43 मेरे देश के सार्वजनिक स्वास्थ्य प्राधिकरण

प्र44 आपको इस बात का कितना भरोसा है कि हम COVID-19 महामारी की तुलना में स्वास्थ्य संबंधी अगली आपदा को बेहतर तरीके से प्रबंधित कर लेंगे?

1 बिल्कुल भी भरोसा नहीं

2 कुछ हद तक भरोसा

3 बहुत अधिक भरोसा

4 पता नहीं

प्र45 आपने जो पढ़ा या सुना है, क्या आप सोचते हैं कि मौसम में बदलाव/ग्लोबल वॉर्मिंग वाकई खतरा है या बिल्कुल भी खतरा नहीं है?

1 वाकई खतरा है

2 बिल्कुल भी खतरा नहीं है

प्र46 आपकी उम्र क्या है?

प्र47 आप यह हैं....?

1 पुरुष

2 महिला

3 बताना पसंद नहीं करेंगे

प्र48 शिक्षा (देश विशिष्ट)

1 अपूर्ण सेकंडरी शिक्षा

2 सेकंडरी शिक्षा पूर्ण

3 यूनिवर्सिटी की कुछ शिक्षा या वोकेशनल प्रमाणपत्र

4 वोकेशनल या पेशेवर प्रमाणपत्र पूर्ण किया

5 यूनिवर्सिटी शिक्षा पूर्ण

6 पोस्ट ग्रेजुएट शिक्षा पूर्ण

7 डॉक्टोरेट, पोस्ट डॉक्टोरेट या इसके समतुल्य शिक्षा पूर्ण

प्र49 माध्यमिक आय स्तर (देश विशिष्ट)

1 हां, मेरी मासिक आय 16,211 भारतीय रुपये है।

2 हां, मेरी मासिक आय 16,211 भारतीय रुपयों से कम है।

3 नहीं, मेरी कोई आय नहीं है।

4 अस्वीकार्य / उत्तर नहीं दिया

प्र50 क्षेत्र (देश विशिष्ट)

1 मध्य प्रदेश

2 पूर्वी भारत: पश्चिम बंगाल, ओडिशा, झारखंड, बिहार, छत्तीसगढ़

3 उत्तरी भारत: हरियाणा, हिमाचल प्रदेश, पंजाब, राजस्थान, उत्तर प्रदेश, उत्तराखंड

4 उत्तर-पूर्वी भारत: त्रिपुरा, सिक्किम, नगालैंड, मिज़ोरम, मेघालय, मणिपुर, आसाम, अरुणाचल प्रदेश

5 दक्षिणी भारत: आंध्र प्रदेश,कर्नाटक, केरल, तमिलनाडु, तेलंगाना

6 पश्चिमी भारत:गोआ,गुजरात,महाराष्ट्र

INDIA

COVID-VAC: A global survey of COVID-19 vaccine perceptions

The purpose of the survey is to measure resident's reactions to COVID-19 response efforts. The risks in this routine survey are considered minimal. You may feel uncomfortable considering the implications of COVID-19 and participation in this research study is completely voluntary and you may discontinue participation at any time. Your responses will remain anonymous and confidential. The survey should take less than 5 minutes to complete. By answering the questions, you are indicating that you have read the description of the study, are over the age of 18, and that you agree to the terms as described.

If you have questions about your rights as a research participant, you may contact the Emerson College Institutional Review Board (IRB), which is concerned with the protection of volunteers in research projects. You may reach the lead investigator via email at emersonpolling@emerson.edu or the Chair of the IRB by e-mailing human_subjects@emerson.edu.

Q1 "First, please indicate your level of agreement with this statement: COVID-19 remains a dangerous health threat."

1 Strongly agree

2 Somewhat agree

3 Unsure/no opinion

4 Somewhat disagree

5 Strongly disagree

Q2 "Now, here are some questions related to COVID-19 vaccines. COVID-19 can be prevented by vaccination."

1 Strongly agree

2 Somewhat agree

3 Unsure/no opinion

4 Somewhat disagree

5 Strongly disagree

Q3 The risks of COVID-19 disease are greater than the risks of the vaccine.

1 Strongly agree

2 Somewhat agree

3 Unsure/no opinion

4 Somewhat disagree

5 Strongly disagree

Q4 The COVID-19 vaccines available to me are safe.

1 Strongly agree

2 Somewhat agree

3 Unsure/no opinion

4 Somewhat disagree

5 Strongly disagree

Q5 I trust the science behind the COVID-19 vaccines available to me.

1 Strongly agree

2 Somewhat agree

3 Unsure/no opinion

4 Somewhat disagree

5 Strongly disagree

Q6 The COVID-19 vaccines available to me are effective in protecting against severe COVID-19.

1 Strongly agree

2 Somewhat agree

3 Unsure/no opinion

4 Somewhat disagree

5 Strongly disagree

Q7 Have you received at least one dose of a COVID-19 vaccine?

1 Yes, I received one dose

2 Yes, I received two or more doses

3 No

Q8 Which of the following COVID-19 vaccines did you receive? Select all that apply:

1 Moderna (Spikevax)

2 Pfizer (Comirnaty)

3 Johnson&Johnson (Janssen or J&J)

4 Sinovac

5 Astra-Zeneca (Oxford, Vaxzevria)

6 COVAXIN

7 Other (please specify)

8 Don't know

Q9 I will take the recommended COVID-19 booster.

1 Strongly agree

2 Somewhat agree

3 Unsure/no opinion

4 Somewhat disagree

5 Strongly disagree

Q10 I believe the COVID-19 pandemic is over.

1 Strongly agree

2 Somewhat agree

3 Unsure/no opinion

4 Somewhat disagree

5 Strongly disagree

Q11 How much did you trust your government's management of the COVID-19 pandemic in your country?

1 Did not trust

2 Somewhat did not trust

3 Unsure

4 Somewhat trust

5 Trust

6 Don't know or don't remember

Q12 Did the development of the COVID-19 vaccines affect your trust in the pharmaceutical industry?

1 Increased trust

2 No effect

3 Decreased trust

4 Don't know or don't remember

Q13 Did the development of COVID-19 vaccines affect your trust in science generally?

1 Increased trust

2 No effect

3 Decreased trust

4 Don't know or don't remember

Q14 How much do you trust the health authorities that recommended you get a COVID-19 vaccine?

1 Not at all

2 A little

3 Moderately

4 Very much

5 Don’t know or don’t remember

Q15 During the pandemic, did you have to delay or cancel any medical services that you needed unrelated to COVID-19?

1 Yes

2 No

3 Don’t know or don’t remember

Q16 Would you follow a vaccine requirement (mandate) in the future from any of the following? Select all that apply:

1 Government

2 Employer

3 School or university

4 None of the above

5 Don’t know

Q17 Would you follow a face mask requirement (mandate) in the future from any of the following? Select all that apply:

1 Government

2 Employer

3 School or university

4 None of the above

5 Don’t know

Q18 To your knowledge, do you have or have you had COVID-19?

1 Yes

2 No

3 Don’t know or don’t remember

Q19 "Long-COVID has been defined as 'symptoms that can last for weeks or months after recovery from acute illness'. To your knowledge, do you have or have you had Long COVID? "

1 Yes

2 No

3 Don’t know or don’t remember

Q20 Did you take any of the following medicines to treat COVID-19? Select all that apply:

1 Paxlovid

2 Molnupiravir (Lagevrio)

3 Monoclonal antibodies (Olumiant/Baricitinib)

4 Ivermectin

5 Traditional medicine, herbal extracts and treatments

6 Don’t know or don’t remember

7 No, I did not take medicines for COVID-19

Q21 Have you lost a family member or close friend to COVID-19 disease? Select all that apply:

1 Yes, within the past year

2 Yes, more than a year ago

3 No

Q22 I was satisfied with the communication efforts on COVID-19 vaccines made by health authorities in my country.

1 Strongly agree

2 Somewhat agree

3 Unsure/no opinion/don’t remember

4 Somewhat disagree

5 Strongly disagree

Q23 It is easy for me to know the difference between accurate and false information about the COVID-19 vaccine on the Internet.

1 Strongly agree

2 Somewhat agree

3 Unsure/no opinion/don’t remember

4 Somewhat disagree

5 Strongly disagree

Q24 I continue to pay attention to information on COVID-19 vaccines.

1 Strongly agree

2 Somewhat agree

3 Unsure/no opinion/don't remember

4 Somewhat disagree

5 Strongly disagree

Q25 I continue to pay attention to information on vaccines in general.

1 Strongly agree

2 Somewhat agree

3 Unsure/no opinion

4 Somewhat disagree

5 Strongly disagree

Q26 The COVID-19 pandemic has made me more willing to get vaccinated against other diseases (e.g., flu, measles, viral hepatitis B).

1 Strongly agree

2 Somewhat agree

3 Unsure/no opinion

4 Somewhat disagree

5 Strongly disagree

Q27 My experience with the COVID-19 vaccine has affected my trust in other vaccines.

1 Increased trust

2 No effect

3 Decreased trust

4 Don't know

Q28 Broadcast media coverage on COVID-19 vaccines has affected my trust in other vaccines.

1 Increased trust

2 No effect

3 Decreased trust

4 Don’t know

Q29 Social media coverage on COVID-19 vaccines has affected my trust in other vaccines.

1 Increased trust

2 No effect

3 Decreased trust

4 Don’t know

Q30 If an mRNA vaccine is approved for a disease for which you are at risk, would you take it?

1 Definitely no

2 Unsure, but leaning towards no

3 Unsure, but leaning towards yes

4 Definitely yes

Q31 If the World Health Organization (WHO) announced a new pandemic threat, would you trust this information?

1 Yes

2 No

3 Don’t know

Q32 If the World Health Organization (WHO) announced a new pandemic threat and advised getting vaccinated, would you?

1 Yes

2 No

3 Don’t know

Q33 "On a scale of 1 (trust completely) to 10 (do not trust at all) how much do you trust the following sources of information about COVID-19 vaccines: My family and friends"

Q34 My employer

Q35 My doctor or nurse

Q36 My government

Q37 Religious leaders

Q38 News media (e.g., television, Internet, radio, newspapers)

Q39 Social media (e.g., Facebook, Twitter [X], Instagram, WhatsApp, LinkedIn, TikTok)

Q40 World Health Organization (WHO)

Q41 Centers for Disease Control and Prevention (CDC), USA

Q42 European Centre for Disease Prevention and Control (ECDC)

Q43 The public health authorities in my country

Q44 How confident are you that we will manage the next health crisis better than the COVID-19 pandemic?

1 Not at all confident

2 Somewhat confident

3 Very confident

4 Don’t know

Q45 From what you have read or heard, do you think climate change/global warming is a real threat or not a real threat?

1 Real threat

2 Not a real threat

Q46 What is your age?

Q47 What is your gender?

1 Male

2 Female

3 Prefer not to say

Q48 Education (country specific)

1 Incomplete Secondary Education

2 Secondary Education Completed

3 Some University or Vocational Certification

4 Vocational or Professional Certification Completed

5 University Education Completed

6 Postgraduate Education Completed

7 Doctorate, Post-doctorate or equivalent Completed

Q49 Median Income Level (country specific)

1 Yes, my monthly income is more than 25,562 Kenyan Shillings.

2 Yes, my monthly income is less than 25,562 Kenyan Shillings.

3 No, I do not have an income.

4 Refused/ Did not answer

Q50 Regions (country specific)

1 Madhya Pradesh

2 East India: West Bengal, Odisha, Jharkhand, Bihar, Chhattisgarh

3 North India: Haryana, Himachal Pradesh, Punjab, Rajasthan, Uttar Pradesh, Uttarakhand

4 Northeast India: Tripura, Sikkim, Nagaland, Mizoram, Meghalaya, Manipur, Assam, Arunachal Pradesh

5 South India: Andhra Pradesh, Karnataka, Kerala, Tamil Nadu, Telangana

6 Western India: Goa, Gujarat, Maharashtra

Italy

COVID-VAC: Un sondaggio globale sulle percezioni del vaccino per il COVID-19

Lo scopo di questo sondaggio è valutare le reazioni degli abitanti agli sforzi di contenimento del COVID-19. I rischi di questi sondaggi di routine sono minimi. In alcuni frangenti Lei potrebbe sentirsi a disagio a considerare le implicazioni del COVID-19. La partecipazione a questo studio di ricerca è completamente volontaria e Lei può interromperla in qualsiasi momento. Le Sue risposte resteranno anonime e riservate. Il sondaggio richiederà meno di 5 minuti per essere completato. Rispondendo alle domande, Lei dichiara di aver letto la descrizione dello studio, di avere oltre 18 anni di età e di accettare i termini come descritti.

Per qualsiasi domanda sui Suoi diritti in quanto partecipante alla ricerca può contattare l'Emerson College Institutional Review Board (IRB), che si occupa della tutela dei volontari nei progetti di ricerca. Può contattare il responsabile via email all'indirizzo emersonpolling@emerson.edu o il Consiglio di IRB via email all'indirizzo human_subjects@emerson.edu.

Q1 "Per prima cosa, indichi il Suo livello di accordo con questa affermazione: Il COVID-19 rimane una pericolosa minaccia per la salute."

1 Fortemente d'accordo

2 Abbastanza d'accordo

3 Non sono sicuro/non ho un'opinione

4 Abbastanza in disaccordo

5 Fortemente in disaccordo

Q2 "Ora vorremmo porLe alcune domande relative ai vaccini COVID-19. La vaccinazione può prevenire il COVID-19."

1 Fortemente d'accordo

2 Abbastanza d'accordo

3 Non sono sicuro/non ho un'opinione

4 Abbastanza in disaccordo

5 Fortemente in disaccordo

Q3 I rischi legati al COVID-19 sono maggiori dei rischi del vaccino.

1 Fortemente d'accordo

2 Abbastanza d'accordo

3 Non sono sicuro/non ho un'opinione

4 Abbastanza in disaccordo

5 Fortemente in disaccordo

Q4 I vaccini COVID-19 a mia disposizione sono sicuri.

1 Fortemente d'accordo

2 Abbastanza d'accordo

3 Non sono sicuro/non ho un'opinione

4 Abbastanza in disaccordo

5 Fortemente in disaccordo

Q5 Mi fido della scienza alla base dei vaccini COVID-19 a mia disposizione.

1 Fortemente d'accordo

2 Abbastanza d'accordo

3 Non sono sicuro/non ho un'opinione

4 Abbastanza in disaccordo

5 Fortemente in disaccordo

Q6 I vaccini COVID-19 a mia disposizione sono efficaci nella protezione contro le forme più gravi di COVID-19.

1 Fortemente d'accordo

2 Abbastanza d'accordo

3 Non sono sicuro/non ho un'opinione

4 Abbastanza in disaccordo

5 Fortemente in disaccordo

Q7 Ha ricevuto almeno una dose di un vaccino COVID-19?

1 Sì, ho ricevuto una dose

2 Sì, ho ricevuto due o più dosi

3 No

Q8 Quali dei seguenti vaccini COVID-19 ha ricevuto? Selezionare tutte le risposte pertinenti:

1 Moderna (Spikevax)

2 Pfizer (Comirnaty)

3 Johnson&Johnson (Janssen o J&J)

4 Sinovac

5 Astra-Zeneca (Oxford, Vaxzevria)

6 COVAXIN

7 Altro (specificare)

8 Non so

Q9 Assumerò il booster COVID-19 consigliato.

1 Fortemente d'accordo

2 Abbastanza d'accordo

3 Non sono sicuro/non ho un'opinione

4 Abbastanza in disaccordo

5 Fortemente in disaccordo

Q10 Credo che la pandemia di COVID-19 sia finita.

1 Fortemente d'accordo

2 Abbastanza d'accordo

3 Non sono sicuro/non ho un'opinione

4 Abbastanza in disaccordo

5 Fortemente in disaccordo

Q11 Quanto si è fidato della gestione della pandemia di COVID-19 da parte del governo del Suo Paese?

1 Non mi sono fidato

2 Mi sono fidato poco

3 Non sono sicuro

4 Mi sono fidato abbastanza

5 Mi sono fidato

6 Non so o non ricordo

Q12 Lo sviluppo dei vaccini COVID-19 ha influito sulla Sua fiducia nell'industria farmaceutica?

1 La fiducia è aumentata

2 Nessun effetto

3 La fiducia è diminuita

4 Non so o non ricordo

Q13 Lo sviluppo dei vaccini COVID-19 ha influito sulla Sua fiducia nella scienza in generale?

1 La fiducia è aumentata

2 Nessun effetto

3 La fiducia è diminuita

4 Non so o non ricordo

Q14 Quanto si fida delle autorità sanitarie che Le hanno raccomandato di fare un vaccino COVID-19?

1 Per nulla

2 Poco

3 Moderatamente

4 Molto

5 Non so o non ricordo

Q15 Durante la pandemia, ha dovuto ritardare o annullare qualsiasi servizio medico di cui aveva bisogno non correlato al COVID-19?

1 SÌ

2 No

3 Non so o non ricordo

Q16 Rispetterebbe un obbligo vaccinale in futuro da uno dei seguenti? Selezionare tutte le risposte pertinenti:

1 Governo

2 Datore di lavoro

3 Scuola o università

4 Nessuna di queste opzioni

5 Non so

Q17 Rispetterebbe un obbligo di uso della mascherina in futuro da uno dei seguenti? Selezionare tutte le risposte pertinenti:

1 Governo

2 Datore di lavoro

3 Scuola o università

4 Nessuna di queste opzioni

5 Non so

Q18 Per quanto ne sa, ha o ha avuto il COVID-19?

1 SÌ

2 No

3 Non so o non ricordo

Q19 "Il "Long COVID" è stato definito come ""sintomi che possono durare per settimane o mesi dopo il recupero da una malattia acuta"". Per quanto ne sa, ha o ha avuto il "Long COVID"? "

1 SÌ

2 No

3 Non so o non ricordo

Q20 Ha assunto uno dei seguenti farmaci per trattare il COVID-19? Selezionare tutte le risposte pertinenti:

1 Paxlovid

2 Molnupiravir (Lagevrio)

3 Anticorpi monoclonali (Olumiant/Baricitinib)

4 Ivermectina

5 Medicina tradizionale, estratti e trattamenti a base di erbe

6 Non so o non ricordo

7 No, non ho assunto farmaci per il COVID-19

Q21 Ha perso un familiare o un amico intimo a causa della malattia da COVID-19? Selezionare tutte le risposte pertinenti:

1 Sì, nell'ultimo anno

2 Sì, più di un anno fa

3 No

Q22 Mi ritengo soddisfatto degli sforzi di comunicazione sui vaccini COVID-19 compiuti dalle autorità sanitarie nel mio paese.

1 Fortemente d'accordo

2 Abbastanza d'accordo

3 Non sono sicuro/non ho opinioni/non ricordo

4 Abbastanza in disaccordo

5 Fortemente in disaccordo

Q23 È facile per me conoscere la differenza tra informazioni vere e false sul vaccino COVID-19 su Internet.

1 Fortemente d'accordo

2 Abbastanza d'accordo

3 Non sono sicuro/non ho opinioni/non ricordo

4 Abbastanza in disaccordo

5 Fortemente in disaccordo

Q24 Continuo a prestare attenzione alle informazioni sui vaccini COVID-19.

1 Fortemente d'accordo

2 Abbastanza d'accordo

3 Non sono sicuro/non ho opinioni/non ricordo

4 Abbastanza in disaccordo

5 Fortemente in disaccordo

Q25 Continuo a prestare attenzione alle informazioni sui vaccini in generale.

1 Fortemente d'accordo

2 Abbastanza d'accordo

3 Non sono sicuro/non ho un'opinione

4 Abbastanza in disaccordo

5 Fortemente in disaccordo

Q26 La pandemia di COVID-19 mi ha reso più disposto a vaccinarmi contro altre malattie (ad es. influenza, morbillo, epatite virale B).

1 Fortemente d'accordo

2 Abbastanza d'accordo

3 Non sono sicuro/non ho un'opinione

4 Abbastanza in disaccordo

5 Fortemente in disaccordo

Q27 La mia esperienza con il vaccino COVID-19 ha influito sulla mia fiducia in altri vaccini.

1 La fiducia è aumentata

2 Nessun effetto

3 La fiducia è diminuita

4 Non so

Q28 La copertura mediatica sui vaccini COVID-19 ha influito sulla mia fiducia in altri vaccini.

1 La fiducia è aumentata

2 Nessun effetto

3 La fiducia è diminuita

4 Non so

Q29 La copertura dei social media sui vaccini COVID-19 ha influito sulla mia fiducia in altri vaccini.

1 La fiducia è aumentata

2 Nessun effetto

3 La fiducia è diminuita

4 Non so

Q30 Se un vaccino mRNA venisse approvato per una malattia per la quale Lei è a rischio, lo prenderebbe?

1 Assolutamente no

2 Incerto, ma credo di no

3 Incerto, ma credo di sì

4 Assolutamente sì

Q31 Se l'Organizzazione Mondiale della Sanità (OMS) annunciasse una nuova minaccia pandemica, si fiderebbe di queste informazioni?

1 Sì

2 No

3 Non so

Q32 Se l'Organizzazione Mondiale della Sanità (OMS) annunciasse una nuova minaccia pandemica e consigliasse di vaccinarsi, lo farebbe?

1 Sì

2 No

3 Non so

Q33 "Su una scala da 1 (mi fido completamente) a 10 (non mi fido affatto) quanto si fida delle seguenti fonti di informazioni sui vaccini COVID-19? Amici o parenti"

Q34 Il mio datore di lavoro

Q35 Il mio medico o infermiere

Q36 Il mio governo

Q37 Leader religiosi

Q38 Mezzi di informazione (ad es. televisione, internet, radio, giornali)

Q39 Social media (ad es. Facebook, Twitter [X], Instagram, WhatsApp, LinkedIn, TikTok)

Q40 Organizzazione Mondiale della Sanità (OMS)

Q41 Centri per il controllo e la prevenzione delle malattie (CDC), USA

Q42 Centro europeo per la prevenzione e il controllo delle malattie (ECDC)

Q43 Le autorità sanitarie pubbliche nel mio Paese

Q44 Quanto è fiducioso sul fatto che sapremo gestire la prossima crisi sanitaria meglio della pandemia di COVID-19?

1 Per nulla fiducioso

2 Abbastanza fiducioso

3 Molto fiducioso

4 Non so

Q45 Da quello che ha letto o sentito, pensa che il cambiamento climatico/riscaldamento globale sia una minaccia reale o meno?

1 È una minaccia reale

2 Non è una minaccia reale

Q46 Quanti anni ha?

Q47 Qual è il Suo genere?

1 Uomo

2 Donna

3 Preferisco non rispondere

Q48 Istruzione (specifica per paese)

1 Istruzione Secondaria non completata

2 Istruzione Secondaria completata

3 Certificazione professionale o università non completata

4 Certificazione professionale o università completata

5 Istruzione universitaria completata

6 Istruzione post-universitaria completata

7 Dottorato, post-dottorato o equivalente completato

Q49 Livello di reddito mediano (specifico per paese)

1 Sì, il mio reddito mensile è superiore a 2.832 euro.

2 Sì, il mio reddito mensile è inferiore a 2.832 euro.

3 No, non ho un reddito.

4 Rifiutato/ Non ha risposto

Q50 Regioni (specifica per paese)

1 Abruzzo

2 Basilicata

3 Calabria

4 Campania

5 Emilia-Romagna

6 Friuli-Venezia Giulia

7 Lazio

8 Liguria

9 Lombardia

10 Marche

11	Molise
12	Piemonte
13	Puglia
14	Sardegna
15	Sicilia
16	Toscana
17	Trentino-Alto Adige
18	Umbria
19	Valle d’Aosta
20	Veneto

Kenya

COVID-VAC: A global survey of COVID-19 vaccine perceptions

The purpose of the survey is to measure resident's reactions to COVID-19 response efforts. The risks in this routine survey are considered minimal. You may feel uncomfortable considering the implications of COVID-19 and participation in this research study is completely voluntary and you may discontinue participation at any time. Your responses will remain anonymous and confidential. The survey should take less than 5 minutes to complete. By answering the questions, you are indicating that you have read the description of the study, are over the age of 18, and that you agree to the terms as described.

If you have questions about your rights as a research participant, you may contact the Emerson College Institutional Review Board (IRB), which is concerned with the protection of volunteers in research projects. You may reach the lead investigator via email at emersonpolling@emerson.edu or the Chair of the IRB by e-mailing human_subjects@emerson.edu.

Q1 "First, please indicate your level of agreement with this statement: COVID-19 remains a dangerous health threat."

1 Strongly agree

2 Somewhat agree

3 Unsure/no opinion

4 Somewhat disagree

5 Strongly disagree

Q2 "Now, here are some questions related to COVID-19 vaccines.COVID-19 can be prevented by vaccination."

1 Strongly agree

2 Somewhat agree

3 Unsure/no opinion

4 Somewhat disagree

5 Strongly disagree

Q3 The risks of COVID-19 disease are greater than the risks of the vaccine.

1 Strongly agree

2 Somewhat agree

3 Unsure/no opinion

4 Somewhat disagree

5 Strongly disagree

Q4 The COVID-19 vaccines available to me are safe.

1 Strongly agree

2 Somewhat agree

3 Unsure/no opinion

4 Somewhat disagree

5 Strongly disagree

Q5 I trust the science behind the COVID-19 vaccines available to me.

1 Strongly agree

2 Somewhat agree

3 Unsure/no opinion

4 Somewhat disagree

5 Strongly disagree

Q6 The COVID-19 vaccines available to me are effective in protecting against severe COVID-19.

1 Strongly agree

2 Somewhat agree

3 Unsure/no opinion

4 Somewhat disagree

5 Strongly disagree

Q7 Have you received at least one dose of a COVID-19 vaccine?

1 Yes, I received one dose

2 Yes, I received two or more doses

3 No

Q8 Which of the following COVID-19 vaccines did you receive? Select all that apply:

1 Moderna (Spikevax)

2 Pfizer (Comirnaty)

3 Johnson&Johnson (Janssen or J&J)

4 Sinovac

5 Astra-Zeneca (Oxford, Vaxzevria)

6 COVAXIN

7 Other (please specify)

8 Don't know

Q9 I will take the recommended COVID-19 booster.

1 Strongly agree

2 Somewhat agree

3 Unsure/no opinion

4 Somewhat disagree

5 Strongly disagree

Q10 I believe the COVID-19 pandemic is over.

1 Strongly agree

2 Somewhat agree

3 Unsure/no opinion

4 Somewhat disagree

5 Strongly disagree

Q11 How much did you trust your government's management of the COVID-19 pandemic in your country?

1 Did not trust

2 Somewhat did not trust

3 Unsure

4 Somewhat trust

5 Trust

6 Don't know or don't remember

Q12 Did the development of the COVID-19 vaccines affect your trust in the pharmaceutical industry?

1 Increased trust

2 No effect

3 Decreased trust

4 Don't know or don't remember

Q13 Did the development of COVID-19 vaccines affect your trust in science generally?

1 Increased trust

2 No effect

3 Decreased trust

4 Don't know or don't remember

Q14 How much do you trust the health authorities that recommended you get a COVID-19 vaccine?

1 Not at all

2 A little

3 Moderately

4 Very much

5 Don’t know or don’t remember

Q15 During the pandemic, did you have to delay or cancel any medical services that you needed unrelated to COVID-19?

1 Yes

2 No

3 Don’t know or don’t remember

Q16 Would you follow a vaccine requirement (mandate) in the future from any of the following? Select all that apply:

1 Government

2 Employer

3 School or university

4 None of the above

5 Don’t know

Q17 Would you follow a face mask requirement (mandate) in the future from any of the following? Select all that apply:

1 Government

2 Employer

3 School or university

4 None of the above

5 Don’t know

Q18 To your knowledge, do you have or have you had COVID-19?

1 Yes

2 No

3 Don’t know or don’t remember

Q19 "Long-COVID has been defined as 'symptoms that can last for weeks or months after recovery from acute illness'. To your knowledge, do you have or have you had Long COVID? "

1 Yes

2 No

3 Don’t know or don’t remember

Q20 Did you take any of the following medicines to treat COVID-19? Select all that apply:

1 Paxlovid

2 Molnupiravir (Lagevrio)

3 Monoclonal antibodies (Olumiant/Baricitinib)

4 Ivermectin

5 Traditional medicine, herbal extracts and treatments

6 Don’t know or don’t remember

7 No, I did not take medicines for COVID-19

Q21 Have you lost a family member or close friend to COVID-19 disease? Select all that apply:

1 Yes, within the past year

2 Yes, more than a year ago

3 No

Q22 I was satisfied with the communication efforts on COVID-19 vaccines made by health authorities in my country.

1 Strongly agree

2 Somewhat agree

3 Unsure/no opinion/don’t remember

4 Somewhat disagree

5 Strongly disagree

Q23 It is easy for me to know the difference between accurate and false information about the COVID-19 vaccine on the Internet.

1 Strongly agree

2 Somewhat agree

3 Unsure/no opinion/don’t remember

4 Somewhat disagree

5 Strongly disagree

Q24 I continue to pay attention to information on COVID-19 vaccines.

1 Strongly agree

2 Somewhat agree

3 Unsure/no opinion/don't remember

4 Somewhat disagree

5 Strongly disagree

Q25 I continue to pay attention to information on vaccines in general.

1 Strongly agree

2 Somewhat agree

3 Unsure/no opinion

4 Somewhat disagree

5 Strongly disagree

Q26 The COVID-19 pandemic has made me more willing to get vaccinated against other diseases (e.g., flu, measles, viral hepatitis B).

1 Strongly agree

2 Somewhat agree

3 Unsure/no opinion

4 Somewhat disagree

5 Strongly disagree

Q27 My experience with the COVID-19 vaccine has affected my trust in other vaccines.

1 Increased trust

2 No effect

3 Decreased trust

4 Don't know

Q28 Broadcast media coverage on COVID-19 vaccines has affected my trust in other vaccines.

1 Increased trust

2 No effect

3 Decreased trust

4 Don’t know

Q29 Social media coverage on COVID-19 vaccines has affected my trust in other vaccines.

1 Increased trust

2 No effect

3 Decreased trust

4 Don’t know

Q30 If an mRNA vaccine is approved for a disease for which you are at risk, would you take it?

1 Definitely no

2 Unsure, but leaning towards no

3 Unsure, but leaning towards yes

4 Definitely yes

Q31 If the World Health Organization (WHO) announced a new pandemic threat, would you trust this information?

1 Yes

2 No

3 Don’t know

Q32 If the World Health Organization (WHO) announced a new pandemic threat and advised getting vaccinated, would you?

1 Yes

2 No

3 Don’t know

Q33 "On a scale of 1 (trust completely) to 10 (do not trust at all) how much do you trust the following sources of information about COVID-19 vaccines: My family and friends"

Q34 My employer

Q35 My doctor or nurse

Q36 My government

Q37 Religious leaders

Q38 News media (e.g., television, Internet, radio, newspapers)

Q39 Social media (e.g., Facebook, Twitter [X], Instagram, WhatsApp, LinkedIn, TikTok)

Q40 World Health Organization (WHO)

Q41 Centers for Disease Control and Prevention (CDC), USA

Q42 European Centre for Disease Prevention and Control (ECDC)

Q43 The public health authorities in my country

Q44 How confident are you that we will manage the next health crisis better than the COVID-19 pandemic?

1 Not at all confident

2 Somewhat confident

3 Very confident

4 Don’t know

Q45 From what you have read or heard, do you think climate change/global warming is a real threat or not a real threat?

1 Real threat

2 Not a real threat

Q46 What is your age?

Q47 What is your gender?

1 Male

2 Female

3 Prefer not to say

Q48 Education (country specific)

1 Primary Education

2 Secondary School

3 University Bachelor Degree

4 Postgraduate Education Completed

5 Doctorate, Post-doctorate or equivalent Completed

Q49 Median Income Level (country specific)

1 Yes, my monthly income is more than 25,562 Kenyan Shillings.

2 Yes, my monthly income is less than 25,562 Kenyan Shillings.

3 No, I do not have an income.

4 Refused/ Did not answer

Q50 Regions (country specific)

1 Nairobi

2 Central

3 Coast

4 Eastern

5 North Eastern

6 Nyana

7 Rift Valley

8 Western

Mexico

COVID-VAC: Una encuesta mundial sobre la percepción de la vacuna contra la COVID-19

El propósito de la encuesta es medir las reacciones de los residentes a los esfuerzos de respuesta de la COVID-19. Los riesgos de esta encuesta rutinaria se consideran mínimos. Es posible que se sienta incómodo al considerar las implicaciones de la COVID-19 y la participación en este estudio de investigación es completamente voluntaria y puede interrumpir su participación en cualquier momento. Sus respuestas serán anónimas y confidenciales. La encuesta debería durar menos de 5 minutos. Al responder a las preguntas, indica que ha leído la descripción del estudio, que es mayor de 18 años y que acepta los términos descritos.

Si tiene preguntas sobre sus derechos como participante en una investigación, puede ponerse en contacto con la Junta de Revisión Institucional (IRB) de Emerson College, que se ocupa de la protección de los voluntarios en los proyectos de investigación. Puede ponerse en contacto con el investigador principal a través del correo electrónico emersonpolling@emerson.edu o con el Presidente del IRB a través del correo electrónico human_subjects@emerson.edu.

Q1 "En primer lugar, indique su grado de acuerdo con esta afirmación: El COVID-19 sigue siendo una peligrosa amenaza para la salud."

1 Totalmente de acuerdo

2 Algo de acuerdo

3 No estoy seguro/sin opinión

4 Algo en desacuerdo

5 Totalmente en desacuerdo

Q2 "Aquí hay algunas preguntas relacionadas con las vacunas contra el COVID-19. El COVID-19 se puede prevenir mediante la vacunación."

1 Totalmente de acuerdo

2 Algo de acuerdo

3 No estoy seguro/sin opinión

4 Algo en desacuerdo

5 Totalmente en desacuerdo

Q3 Los riesgos de la enfermedad COVID-19 son mayores que los riesgos de la vacuna.

1 Totalmente de acuerdo

2 Algo de acuerdo

3 No estoy seguro/sin opinión

4 Algo en desacuerdo

5 Totalmente en desacuerdo

Q4 Las vacunas contra el COVID-19 que tengo a mi disposición son seguras.

1 Totalmente de acuerdo

2 Algo de acuerdo

3 No estoy seguro/sin opinión

4 Algo en desacuerdo

5 Totalmente en desacuerdo

Q5 Confío en la ciencia que respalda las vacunas contra el COVID-19 que tengo a mi disposición.

1 Totalmente de acuerdo

2 Algo de acuerdo

3 No estoy seguro/sin opinión

4 Algo en desacuerdo

5 Totalmente en desacuerdo

Q6 Las vacunas contra el COVID-19 que tengo a mi disposición son eficaces para protegerme contra el COVID-19 grave.

1 Totalmente de acuerdo

2 Algo de acuerdo

3 No estoy seguro/sin opinión

4 Algo en desacuerdo

5 Totalmente en desacuerdo

Q7 ¿Ha recibido al menos una dosis de la vacuna contra el COVID-19?

1 Sí, recibí una dosis

2 Sí, recibí dos o más dosis

3 No

Q8 ¿Cuál de las siguientes vacunas contra el COVID-19 recibió? Seleccione todas las que correspondan:

1 Moderna (Spikevax)

2 Pfizer (Comirnaty)

3 Johnson&Johnson (Janssen o J&J)

4 Sinovac

5 Astra-Zeneca (Oxford, Vaxzevria)

6 COVAXIN

7 Otras (especifique)

8 No sé

Q9 Tomaré el refuerzo de COVID-19 recomendado.

1 Totalmente de acuerdo

2 Algo de acuerdo

3 No estoy seguro/sin opinión

4 Algo en desacuerdo

5 Totalmente en desacuerdo

Q10 Creo que la pandemia de COVID-19 ha terminado.

1 Totalmente de acuerdo

2 Algo de acuerdo

3 No estoy seguro/sin opinión

4 Algo en desacuerdo

5 Totalmente en desacuerdo

Q11 ¿En qué medida confió en la gestión gubernamental de la pandemia de COVID-19 en su país?

1 No confié

2 No confié en absoluto

3 No estoy seguro

4 Confié un poco

5 Confíe

6 No lo sé o no me acuerdo

Q12 ¿Afectó el desarrollo de las vacunas contra el COVID-19 su confianza en la industria farmacéutica?

1 Incrementó mi confianza

2 Sin efecto

3 Disminuyó mi confianza

4 No lo sé o no me acuerdo

Q13 ¿Afectó el desarrollo de las vacunas contra el COVID-19 su confianza en la ciencia en general?

1 Incrementó mi confianza

2 Sin efecto

3 Disminuyó mi confianza

4 No lo sé o no me acuerdo

Q14 ¿Hasta qué punto confía en las autoridades sanitarias que le recomendaron vacunarse contra el COVID-19?

1 En absoluto

2 Un poco

3 Moderadamente

4 Mucho

5 No lo sé o no me acuerdo

Q15 Durante la pandemia, ¿tuvo que retrasar o cancelar algún servicio médico que necesitaba no relacionado con el COVID-19?

1 Sí

2 No

3 No lo sé o no me acuerdo

Q16 ¿Seguiría en el futuro una exigencia (mandato) de vacunación de alguno de los siguientes? Seleccione todas las que correspondan:

1 Gobierno

2 Empleador

3 Escuela o universidad

4 Ninguna de las anteriores

5 No sé

Q17 ¿Seguiría en el futuro una exigencia (mandato) de uso de mascarilla de alguno de los siguientes? Seleccione todas las que correspondan:

1 Gobierno

2 Empleador

3 Escuela o universidad

4 Ninguna de las anteriores

5 No sé

Q18 Que usted sepa, ¿tiene o ha tenido COVID-19?

1 Sí

2 No

3 No lo sé o no me acuerdo

Q19 "El COVID prolongado se ha definido como "síntomas que pueden durar semanas o meses después de la recuperación de una enfermedad aguda". Que usted sepa, ¿tiene o ha tenido COVID prolongada? "

1 Sí

2 No

3 No lo sé o no me acuerdo

Q20 ¿Tomó alguno de los siguientes medicamentos para tratar el COVID-19? Seleccione todas las que correspondan:

1 Paxlovid

2 Molnupiravir (Lagevrio)

3 Anticuerpos monoclonales (Olumiant/Baricitinib)

4 Ivermectina

5 Medicina tradicional, extractos y tratamientos a base de plantas

6 No lo sé o no me acuerdo

7 No, no he tomado medicamentos para el COVID-19

Q21 ¿Ha perdido a un familiar o amigo cercano a causa del COVID-19? Seleccione todas las que correspondan:

1 Sí, en el último año

2 Sí, hace más de un año

3 No

Q22 Estuve satisfecho con los esfuerzos de comunicación sobre las vacunas contra el COVID-19 realizados por las autoridades sanitarias de mi país.

1 Totalmente de acuerdo

2 Algo de acuerdo

3 No estoy seguro/sin opinión/no me acuerdo

4 Algo en desacuerdo

5 Totalmente en desacuerdo

Q23 Para mí es fácil distinguir entre información exacta y falsa sobre la vacuna contra el COVID-19 en Internet.

1 Totalmente de acuerdo

2 Algo de acuerdo

3 No estoy seguro/sin opinión/no me acuerdo

4 Algo en desacuerdo

5 Totalmente en desacuerdo

Q24 Sigo prestando atención a la información sobre las vacunas contra el COVID-19.

1 Totalmente de acuerdo

2 Algo de acuerdo

3 No estoy seguro/sin opinión/no me acuerdo

4 Algo en desacuerdo

5 Totalmente en desacuerdo

Q25 Sigo prestando atención a la información sobre las vacunas en general.

1 Totalmente de acuerdo

2 Algo de acuerdo

3 No estoy seguro/sin opinión

4 Algo en desacuerdo

5 Totalmente en desacuerdo

Q26 La pandemia de COVID-19 ha hecho que esté más dispuesto a vacunarme contra otras enfermedades (p. ej., gripe, sarampión, hepatitis B).

1 Totalmente de acuerdo

2 Algo de acuerdo

3 No estoy seguro/sin opinión

4 Algo en desacuerdo

5 Totalmente en desacuerdo

Q27 Mi experiencia con la vacuna contra el COVID-19 ha afectado mi confianza en otras vacunas.

1 Incrementó mi confianza

2 Sin efecto

3 Disminuyó mi confianza

4 No sé

Q28 La cobertura de los medios de comunicación sobre las vacunas contra el COVID-19 ha afectado mi confianza en otras vacunas.

1 Incrementó mi confianza

2 Sin efecto

3 Disminuyó mi confianza

4 No sé

Q29 La cobertura de las redes sociales sobre las vacunas contra el COVID-19 ha afectado mi confianza en otras vacunas.

1 Incrementó mi confianza

2 Sin efecto

3 Disminuyó mi confianza

4 No sé

Q30 Si se aprobara una vacuna de ARNm para una enfermedad de riesgo, ¿la tomaría?

1 Definitivamente no

2 No estoy seguro, pero me inclino por el no

3 No estoy seguro, pero me inclino por el sí

4 Definitivamente sí

Q31 Si la Organización Mundial de la Salud (OMS) anunciara una nueva amenaza de pandemia, ¿confiaría en esta información?

1 Sí

2 No

3 No sé

Q32 Si la Organización Mundial de la Salud (OMS) anunciara una nueva amenaza de pandemia y recomendara vacunarse, ¿lo haría usted?

1 Sí

2 No

3 No sé

Q33 "En una escala del 1 (confía plenamente) al 10 (no confía en absoluto), ¿en qué medida confía en las siguientes fuentes de información sobre las vacunas contra el COVID-19? Mi familia y mis amigos"

Q34 Mi empleador

Q35 Mi médico o enfermero

Q36 Mi gobierno

Q37 Líderes religiosos

Q38 Medios de comunicación (p. ej., televisión, Internet, radio, periódicos, etc.)

Q39 Redes sociales (p. ej., Facebook, Twitter [X], Instagram, WhatsApp, LinkedIn, TikTok)

Q40 Organización Mundial de la Salud (OMS)

Q41 Centros para el Control y la Prevención de Enfermedades (CDC), EE. UU.

Q42 Centro Europeo para la Prevención y el Control de Enfermedades (ECDC)

Q43 Las autoridades sanitarias de mi país

Q44 ¿Hasta qué punto confía en que gestionaremos la próxima crisis sanitaria mejor que la pandemia de COVID-19?

1 No confío en absoluto

2 Confío un poco

3 Confío mucho

4 No sé

Q45 Por lo que ha leído o escuchado, ¿cree que el cambio climático/calentamiento global es una amenaza real o no?

1 Es una amenaza real

2 No es una amenaza real

Q46 ¿Cuál es su edad?

Q47 ¿Cuál es su género?

1 Hombre

2 Mujer

3 Prefiero no decirlo

Q48 Educación (según el país)

1 Primaria

2 Secundaria

3 Preparatoria

4 Estudios de postgrado

5 Licenciatura universitaria

6 Formación de posgrado completada

7 Doctorado, posdoctorado o equivalente completado

Q49 Nivel promedio de ingresos (según el país)

1 Sí, mis ingresos mensuales superan los 14 671 pesos mexicanos.

2 Sí, mis ingresos mensuales son inferiores a 14 671 pesos mexicanos.

3 No, no tengo ingresos.

4 Rechazado/No contestó

Q50 Regiones (según el país)

1 Baja California,

2 las Tierras bajas costeras del Pacífico,

3 la Meseta Mexicana,

4 la Sierra Madre Oriental,

5 la Sierra Madre Occidental,

6 la Cordillera Neo-Volcánica,

7 la llanura costera del Golfo,

8 las Tierras altas del sur,

9 Península de Yucatán.

Nigeria

COVID-VAC: A global survey of COVID-19 vaccine perceptions

The purpose of the survey is to measure resident's reactions to COVID-19 response efforts. The risks in this routine survey are considered minimal. You may feel uncomfortable considering the implications of COVID-19 and participation in this research study is completely voluntary and you may discontinue participation at any time. Your responses will remain anonymous and confidential. The survey should take less than 5 minutes to complete. By answering the questions, you are indicating that you have read the description of the study, are over the age of 18, and that you agree to the terms as described.

If you have questions about your rights as a research participant, you may contact the Emerson College Institutional Review Board (IRB), which is concerned with the protection of volunteers in research projects. You may reach the lead investigator via email at emersonpolling@emerson.edu or the Chair of the IRB by e-mailing human_subjects@emerson.edu.

Q1 "First, please indicate your level of agreement with this statement: COVID-19 remains a dangerous health threat."

1 Strongly agree

2 Somewhat agree

3 Unsure/no opinion

4 Somewhat disagree

5 Strongly disagree

Q2 "Now, here are some questions related to COVID-19 vaccines. COVID-19 can be prevented by vaccination."

1 Strongly agree

2 Somewhat agree

3 Unsure/no opinion

4 Somewhat disagree

5 Strongly disagree

Q3 The risks of COVID-19 disease are greater than the risks of the vaccine.

1 Strongly agree

2 Somewhat agree

3 Unsure/no opinion

4 Somewhat disagree

5 Strongly disagree

Q4 The COVID-19 vaccines available to me are safe.

1 Strongly agree

2 Somewhat agree

3 Unsure/no opinion

4 Somewhat disagree

5 Strongly disagree

Q5 I trust the science behind the COVID-19 vaccines available to me.

1 Strongly agree

2 Somewhat agree

3 Unsure/no opinion

4 Somewhat disagree

5 Strongly disagree

Q6 The COVID-19 vaccines available to me are effective in protecting against severe COVID-19.

1 Strongly agree

2 Somewhat agree

3 Unsure/no opinion

4 Somewhat disagree

5 Strongly disagree

Q7 Have you received at least one dose of a COVID-19 vaccine?

1 Yes, I received one dose

2 Yes, I received two or more doses

3 No

Q8 Which of the following COVID-19 vaccines did you receive? Select all that apply:

1 Moderna (Spikevax)

2 Pfizer (Comirnaty)

3 Johnson&Johnson (Janssen or J&J)

4 Sinovac

5 Astra-Zeneca (Oxford, Vaxzevria)

6 COVAXIN

7 Other (please specify)

8 Don’t know

Q9 I will take the recommended COVID-19 booster.

1 Strongly agree

2 Somewhat agree

3 Unsure/no opinion

4 Somewhat disagree

5 Strongly disagree

Q10 I believe the COVID-19 pandemic is over.

1 Strongly agree

2 Somewhat agree

3 Unsure/no opinion

4 Somewhat disagree

5 Strongly disagree

Q11 How much did you trust your government's management of the COVID-19 pandemic in your country?

1 Did not trust

2 Somewhat did not trust

3 Unsure

4 Somewhat trust

5 Trust

6 Don't know or don't remember

Q12 Did the development of the COVID-19 vaccines affect your trust in the pharmaceutical industry?

1 Increased trust

2 No effect

3 Decreased trust

4 Don't know or don't remember

Q13 Did the development of COVID-19 vaccines affect your trust in science generally?

1 Increased trust

2 No effect

3 Decreased trust

4 Don't know or don't remember

Q14 How much do you trust the health authorities that recommended you get a COVID-19 vaccine?

1 Not at all

2 A little

3 Moderately

4 Very much

5 Don’t know or don’t remember

Q15 During the pandemic, did you have to delay or cancel any medical services that you needed unrelated to COVID-19?

1 Yes

2 No

3 Don’t know or don’t remember

Q16 Would you follow a vaccine requirement (mandate) in the future from any of the following? Select all that apply:

1 Government

2 Employer

3 School or university

4 None of the above

5 Don’t know

Q17 Would you follow a face mask requirement (mandate) in the future from any of the following? Select all that apply:

1 Government

2 Employer

3 School or university

4 None of the above

5 Don’t know

Q18 To your knowledge, do you have or have you had COVID-19?

1 Yes

2 No

3 Don’t know or don’t remember

Q19 "Long-COVID has been defined as 'symptoms that can last for weeks or months after recovery from acute illness'. To your knowledge, do you have or have you had Long COVID? "

1 Yes

2 No

3 Don’t know or don’t remember

Q20 Did you take any of the following medicines to treat COVID-19? Select all that apply:

1 Paxlovid

2 Molnupiravir (Lagevrio)

3 Monoclonal antibodies (Olumiant/Baricitinib)

4 Ivermectin

5 Traditional medicine, herbal extracts and treatments

6 Don’t know or don’t remember

7 No, I did not take medicines for COVID-19

Q21 Have you lost a family member or close friend to COVID-19 disease? Select all that apply:

1 Yes, within the past year

2 Yes, more than a year ago

3 No

Q22 I was satisfied with the communication efforts on COVID-19 vaccines made by health authorities in my country.

1 Strongly agree

2 Somewhat agree

3 Unsure/no opinion/don’t remember

4 Somewhat disagree

5 Strongly disagree

Q23 It is easy for me to know the difference between accurate and false information about the COVID-19 vaccine on the Internet.

1 Strongly agree

2 Somewhat agree

3 Unsure/no opinion/don’t remember

4 Somewhat disagree

5 Strongly disagree

Q24 I continue to pay attention to information on COVID-19 vaccines.

1 Strongly agree

2 Somewhat agree

3 Unsure/no opinion/don't remember

4 Somewhat disagree

5 Strongly disagree

Q25 I continue to pay attention to information on vaccines in general.

1 Strongly agree

2 Somewhat agree

3 Unsure/no opinion

4 Somewhat disagree

5 Strongly disagree

Q26 The COVID-19 pandemic has made me more willing to get vaccinated against other diseases (e.g., flu, measles, viral hepatitis B).

1 Strongly agree

2 Somewhat agree

3 Unsure/no opinion

4 Somewhat disagree

5 Strongly disagree

Q27 My experience with the COVID-19 vaccine has affected my trust in other vaccines.

1 Increased trust

2 No effect

3 Decreased trust

4 Don't know

Q28 Broadcast media coverage on COVID-19 vaccines has affected my trust in other vaccines.

1 Increased trust

2 No effect

3 Decreased trust

4 Don’t know

Q29 Social media coverage on COVID-19 vaccines has affected my trust in other vaccines.

1 Increased trust

2 No effect

3 Decreased trust

4 Don’t know

Q30 If an mRNA vaccine is approved for a disease for which you are at risk, would you take it?

1 Definitely no

2 Unsure, but leaning towards no

3 Unsure, but leaning towards yes

4 Definitely yes

Q31 If the World Health Organization (WHO) announced a new pandemic threat, would you trust this information?

1 Yes

2 No

3 Don’t know

Q32 If the World Health Organization (WHO) announced a new pandemic threat and advised getting vaccinated, would you?

1 Yes

2 No

3 Don’t know

Q33 "On a scale of 1 (trust completely) to 10 (do not trust at all) how much do you trust the following sources of information about COVID-19 vaccines: My family and friends"

Q34 My employer

Q35 My doctor or nurse

Q36 My government

Q37 Religious leaders

Q38 News media (e.g., television, Internet, radio, newspapers)

Q39 Social media (e.g., Facebook, Twitter [X], Instagram, WhatsApp, LinkedIn, TikTok)

Q40 World Health Organization (WHO)

Q41 Centers for Disease Control and Prevention (CDC), USA

Q42 European Centre for Disease Prevention and Control (ECDC)

Q43 The public health authorities in my country

Q44 How confident are you that we will manage the next health crisis better than the COVID-19 pandemic?

1 Not at all confident

2 Somewhat confident

3 Very confident

4 Don’t know

Q45 From what you have read or heard, do you think climate change/global warming is a real threat or not a real threat?

1 Real threat

2 Not a real threat

Q46 What is your age?

Q47 What is your gender?

1 Male

2 Female

3 Prefer not to say

Q48 Education (country specific)

1 Primary

2 Junior Secondary

3 Senior Secondary

4 Technival or Vocational

5 Higher techinical

6 Undergraduate teritary program

7 Masters degree

8 Doctorate degree

Q49 Median Income Level (country specific)

1 Yes, my monthly income is more than 140,884 Nigerian Naira.

2 Yes, my monthly income is less than 140,884 Nigerian Naira.

3 No, I do not have an income.

4 Refused/ Did not answer

Q50 Regions (country specific)

1 North Central (also known as Middle Belt): Benue. Kogi. Kwara. Nasarawa. Niger.

2 North East: Adamawa. Bauchi. Borno. Gombe. Taraba.

3 North West: Jigawa. Kaduna. Kano. Katsina.

4 South East: Abia. Anambra. Ebonyi. Enugu. .

5 South South (also known as Niger Delta region) Akwa Ibom. Bayelsa. Cross River. Rivers.

6 South West: Ekiti. Lagos. Ogun. Ondo.

Peru

COVID-VAC: Una encuesta mundial sobre la percepción de la vacuna contra la COVID-19

El propósito de la encuesta es medir las reacciones de los residentes a los esfuerzos de respuesta de la COVID-19. Los riesgos de esta encuesta rutinaria se consideran mínimos. Es posible que se sienta incómodo al considerar las implicaciones de la COVID-19 y la participación en este estudio de investigación es completamente voluntaria y puede interrumpir su participación en cualquier momento. Sus respuestas serán anónimas y confidenciales. La encuesta debería durar menos de 5 minutos. Al responder a las preguntas, indica que ha leído la descripción del estudio, que es mayor de 18 años y que acepta los términos descritos.

Si tiene preguntas sobre sus derechos como participante en una investigación, puede ponerse en contacto con la Junta de Revisión Institucional (IRB) de Emerson College, que se ocupa de la protección de los voluntarios en los proyectos de investigación. Puede ponerse en contacto con el investigador principal a través del correo electrónico emersonpolling@emerson.edu o con el Presidente del IRB a través del correo electrónico human_subjects@emerson.edu.

Q1 "En primer lugar, indique su grado de acuerdo con esta afirmación: El COVID-19 sigue siendo una peligrosa amenaza para la salud."

1 Totalmente de acuerdo

2 Algo de acuerdo

3 No estoy seguro/sin opinión

4 Algo en desacuerdo

5 Totalmente en desacuerdo

Q2 "Aquí hay algunas preguntas relacionadas con las vacunas contra el COVID-19. El COVID-19 se puede prevenir mediante la vacunación."

1 Totalmente de acuerdo

2 Algo de acuerdo

3 No estoy seguro/sin opinión

4 Algo en desacuerdo

5 Totalmente en desacuerdo

Q3 Los riesgos de la enfermedad COVID-19 son mayores que los riesgos de la vacuna.

1 Totalmente de acuerdo

2 Algo de acuerdo

3 No estoy seguro/sin opinión

4 Algo en desacuerdo

5 Totalmente en desacuerdo

Q4 Las vacunas contra el COVID-19 que tengo a mi disposición son seguras.

1 Totalmente de acuerdo

2 Algo de acuerdo

3 No estoy seguro/sin opinión

4 Algo en desacuerdo

5 Totalmente en desacuerdo

Q5 Confío en la ciencia que respalda las vacunas contra el COVID-19 que tengo a mi disposición.

1 Totalmente de acuerdo

2 Algo de acuerdo

3 No estoy seguro/sin opinión

4 Algo en desacuerdo

5 Totalmente en desacuerdo

Q6 Las vacunas contra el COVID-19 que tengo a mi disposición son eficaces para protegerme contra el COVID-19 grave.

1 Totalmente de acuerdo

2 Algo de acuerdo

3 No estoy seguro/sin opinión

4 Algo en desacuerdo

5 Totalmente en desacuerdo

Q7 ¿Ha recibido al menos una dosis de la vacuna contra el COVID-19?

1 Sí, recibí una dosis

2 Sí, recibí dos o más dosis

3 No

Q8 ¿Cuál de las siguientes vacunas contra el COVID-19 recibió? Seleccione todas las que correspondan:

1 Moderna (Spikevax)

2 Pfizer (Comirnaty)

3 Johnson&Johnson (Janssen o J&J)

4 Sinovac

5 Astra-Zeneca (Oxford, Vaxzevria)

6 COVAXIN

7 Otras (especifique)

8 No sé

Q9 Tomaré el refuerzo de COVID-19 recomendado.

1 Totalmente de acuerdo

2 Algo de acuerdo

3 No estoy seguro/sin opinión

4 Algo en desacuerdo

5 Totalmente en desacuerdo

Q10 Creo que la pandemia de COVID-19 ha terminado.

1 Totalmente de acuerdo

2 Algo de acuerdo

3 No estoy seguro/sin opinión

4 Algo en desacuerdo

5 Totalmente en desacuerdo

Q11 ¿En qué medida confió en la gestión gubernamental de la pandemia de COVID-19 en su país?

1 No confié

2 No confié en absoluto

3 No estoy seguro

4 Confié un poco

5 Confíe

6 No lo sé o no me acuerdo

Q12 ¿Afectó el desarrollo de las vacunas contra el COVID-19 su confianza en la industria farmacéutica?

1 Incrementó mi confianza

2 Sin efecto

3 Disminuyó mi confianza

4 No lo sé o no me acuerdo

Q13 ¿Afectó el desarrollo de las vacunas contra el COVID-19 su confianza en la ciencia en general?

1 Incrementó mi confianza

2 Sin efecto

3 Disminuyó mi confianza

4 No lo sé o no me acuerdo

Q14 ¿Hasta qué punto confía en las autoridades sanitarias que le recomendaron vacunarse contra el COVID-19?

1 En absoluto

2 Un poco

3 Moderadamente

4 Mucho

5 No lo sé o no me acuerdo

Q15 Durante la pandemia, ¿tuvo que retrasar o cancelar algún servicio médico que necesitaba no relacionado con el COVID-19?

1 Sí

2 No

3 No lo sé o no me acuerdo

Q16 ¿Seguiría en el futuro una exigencia (mandato) de vacunación de alguno de los siguientes? Seleccione todas las que correspondan:

1 Gobierno

2 Empleador

3 Escuela o universidad

4 Ninguna de las anteriores

5 No sé

Q17 ¿Seguiría en el futuro una exigencia (mandato) de uso de mascarilla de alguno de los siguientes? Seleccione todas las que correspondan:

1 Gobierno

2 Empleador

3 Escuela o universidad

4 Ninguna de las anteriores

5 No sé

Q18 Que usted sepa, ¿tiene o ha tenido COVID-19?

1 Sí

2 No

3 No lo sé o no me acuerdo

Q19 "El COVID prolongado se ha definido como "síntomas que pueden durar semanas o meses después de la recuperación de una enfermedad aguda". Que usted sepa, ¿tiene o ha tenido COVID prolongada? "

1 Sí

2 No

3 No lo sé o no me acuerdo

Q20 ¿Tomó alguno de los siguientes medicamentos para tratar el COVID-19? Seleccione todas las que correspondan:

1 Paxlovid

2 Molnupiravir (Lagevrio)

3 Anticuerpos monoclonales (Olumiant/Baricitinib)

4 Ivermectina

5 Medicina tradicional, extractos y tratamientos a base de plantas

6 No lo sé o no me acuerdo

7 No, no he tomado medicamentos para el COVID-19

Q21 ¿Ha perdido a un familiar o amigo cercano a causa del COVID-19? Seleccione todas las que correspondan:

1 Sí, en el último año

2 Sí, hace más de un año

3 No

Q22 Estuve satisfecho con los esfuerzos de comunicación sobre las vacunas contra el COVID-19 realizados por las autoridades sanitarias de mi país.

1 Totalmente de acuerdo

2 Algo de acuerdo

3 No estoy seguro/sin opinión/no me acuerdo

4 Algo en desacuerdo

5 Totalmente en desacuerdo

Q23 Para mí es fácil distinguir entre información exacta y falsa sobre la vacuna contra el COVID-19 en Internet.

1 Totalmente de acuerdo

2 Algo de acuerdo

3 No estoy seguro/sin opinión/no me acuerdo

4 Algo en desacuerdo

5 Totalmente en desacuerdo

Q24 Sigo prestando atención a la información sobre las vacunas contra el COVID-19.

1 Totalmente de acuerdo

2 Algo de acuerdo

3 No estoy seguro/sin opinión/no me acuerdo

4 Algo en desacuerdo

5 Totalmente en desacuerdo

Q25 Sigo prestando atención a la información sobre las vacunas en general.

1 Totalmente de acuerdo

2 Algo de acuerdo

3 No estoy seguro/sin opinión

4 Algo en desacuerdo

5 Totalmente en desacuerdo

Q26 La pandemia de COVID-19 ha hecho que esté más dispuesto a vacunarme contra otras enfermedades (p. ej., gripe, sarampión, hepatitis B).

1 Totalmente de acuerdo

2 Algo de acuerdo

3 No estoy seguro/sin opinión

4 Algo en desacuerdo

5 Totalmente en desacuerdo

Q27 Mi experiencia con la vacuna contra el COVID-19 ha afectado mi confianza en otras vacunas.

1 Incrementó mi confianza

2 Sin efecto

3 Disminuyó mi confianza

4 No sé

Q28 La cobertura de los medios de comunicación sobre las vacunas contra el COVID-19 ha afectado mi confianza en otras vacunas.

1 Incrementó mi confianza

2 Sin efecto

3 Disminuyó mi confianza

4 No sé

Q29 La cobertura de las redes sociales sobre las vacunas contra el COVID-19 ha afectado mi confianza en otras vacunas.

1 Incrementó mi confianza

2 Sin efecto

3 Disminuyó mi confianza

4 No sé

Q30 Si se aprobara una vacuna de ARNm para una enfermedad de riesgo, ¿la tomaría?

1 Definitivamente no

2 No estoy seguro, pero me inclino por el no

3 No estoy seguro, pero me inclino por el sí

4 Definitivamente sí

Q31 Si la Organización Mundial de la Salud (OMS) anunciara una nueva amenaza de pandemia, ¿confiaría en esta información?

1 Sí

2 No

3 No sé

Q32 Si la Organización Mundial de la Salud (OMS) anunciara una nueva amenaza de pandemia y recomendara vacunarse, ¿lo haría usted?

1 Sí

2 No

3 No sé

Q33 "En una escala del 1 (confía plenamente) al 10 (no confía en absoluto), ¿en qué medida confía en las siguientes fuentes de información sobre las vacunas contra el COVID-19? Mi familia y mis amigos"

Q34 Mi empleador

Q35 Mi médico o enfermero

Q36 Mi gobierno

Q37 Líderes religiosos

Q38 Medios de comunicación (p. ej., televisión, Internet, radio, periódicos, etc.)

Q39 Redes sociales (p. ej., Facebook, Twitter [X], Instagram, WhatsApp, LinkedIn, TikTok)

Q40 Organización Mundial de la Salud (OMS)

Q41 Centros para el Control y la Prevención de Enfermedades (CDC), EE. UU.

Q42 Centro Europeo para la Prevención y el Control de Enfermedades (ECDC)

Q43 Las autoridades sanitarias de mi país

Q44 ¿Hasta qué punto confía en que gestionaremos la próxima crisis sanitaria mejor que la pandemia de COVID-19?

1 No confío en absoluto

2 Confío un poco

3 Confío mucho

4 No sé

Q45 Por lo que ha leído o escuchado, ¿cree que el cambio climático/calentamiento global es una amenaza real o no?

1 Es una amenaza real

2 No es una amenaza real

Q46 ¿Cuál es su edad?

Q47 ¿Cuál es su género?

1 Hombre

2 Mujer

3 Prefiero no decirlo

Q48 Educación (según el país)

1 Menos de Bachillerato

2 Bachillerato o GRE

3 Algunos estudios universitarios

4 Diplomatura o Formación Profesional

5 Licenciatura

6 Título de postgrado (máster, abogado, doctor)

Q49 Nivel promedio de ingresos (según el país)

1 Sí, mis ingresos mensuales superan los 1,997 Peruvian Nuevo Sol.

2 Sí, mis ingresos mensuales son inferiores a 1,997 Peruvian Nuevo Sol.

3 No, no tengo ingresos.

4 Rechazado/No contestó

Q50 Regiones (según el país)

1 Costa (zona costera)

2 Sierra

3 Selva amazónica.

Poland

COVID-SZCZEPIENIA: Globalna ankieta poświęcona postrzeganiu sczepień przeciwko COVID-19

Celem ankiety jest pomiar reakcji rezydentów na działania związane z pandemią COVID-19. Ryzyko w tej rutynowej ankiecie jest uważane za minimalne. Może się Pan(i) poczuć niekomfortowo, mając na uwadze implikacje pandemii COVID-19, więc udział w tym badaniu jest całkowicie dobrowolny i może Pan(i) przerwać udział w dowolnym czasie. Pana/Pani odpowiedzi będą traktowane jako anonimowe i poufne. Wypełnienie ankiety powinno Panu/Pani zająć mniej niż 5 minut. Udzielenie odpowiedzi na pytania oznacza, że zapoznał(a) się Pan(i) z opisem badania, ma Pan(i) ukończone 18 lat i akceptuje Pan(i) warunki ankiety.

W razie jakichkolwiek pytań dotyczących praw przysługujących Panu/Pani jako uczestnikowi badania może się Pan(i) kontaktować z radą Emerson College Institutional Review Board (IRB), która dba o bezpieczeństwo wolontariuszy w projektach badawczych. Może się Pan(i) skontaktować z głównym badaczem po adresem emersonpolling@emerson.edu lub z przewodniczącym rady IRB pod adresem human_subjects@emerson.edu.

Q1 "Po pierwsze proszę wskazać, w jakim stopniu zgadza się Pan/Pani z następującym stwierdzeniem: COVID-19 nadal pozostaje poważnym zagrożeniem dla zdrowia."

1 Stanowczo się zgadzam

2 Raczej się zgadzam

3 Nie mam pewności/nie mam zdania

4 Raczej się nie zgadzam

5 Stanowczo się nie zgadzam

Q2 "Teraz zadamy kilka pytań dotyczących szczepionek przeciwko COVID-19. Zakażeniu się COVID-19 można zapobiegać dzięki szczepieniu."

1 Stanowczo się zgadzam

2 Raczej się zgadzam

3 Nie mam pewności/nie mam zdania

4 Raczej się nie zgadzam

5 Stanowczo się nie zgadzam

Q3 Ryzyko związane z chorobą COVID-19 jest większe niż ryzyko związane ze szczepionką.

1 Stanowczo się zgadzam

2 Raczej się zgadzam

3 Nie mam pewności/nie mam zdania

4 Raczej się nie zgadzam

5 Stanowczo się nie zgadzam

Q4 Dostępne dla mnie szczepionki przeciwko COVID-19 są bezpieczne.

1 Stanowczo się zgadzam

2 Raczej się zgadzam

3 Nie mam pewności/nie mam zdania

4 Raczej się nie zgadzam

5 Stanowczo się nie zgadzam

Q5 Wierzę w naukę, która stworzyła dostępne dla mnie szczepionki przeciwko COVID-19.

1 Stanowczo się zgadzam

2 Raczej się zgadzam

3 Nie mam pewności/nie mam zdania

4 Raczej się nie zgadzam

5 Stanowczo się nie zgadzam

Q6 Dostępne dla mnie szczepionki przeciwko COVID-19 skutecznie chronią przed ciężkim przebiegiem choroby COVID-19.

1 Stanowczo się zgadzam

2 Raczej się zgadzam

3 Nie mam pewności/nie mam zdania

4 Raczej się nie zgadzam

5 Stanowczo się nie zgadzam

Q7 Czy przyjął Pan/przyjęła Pani co najmniej jedną dawkę szczepionki przeciwko COVID-19?

1 Tak, przyjąłem/przyjęłam jedną dawkę

2 Tak, przyjąłem/przyjęłam dwie lub więcej dawek

3 Nie

Q8 Którą z następujących szczepionek przeciwko COVID-19 Pan przyjął/Pani przyjęła? Proszę wybrać wszystkie odpowiedzi, które mają zastosowanie:

1 Moderna (Spikevax)

2 Pfizer (Comirnaty)

3 Johnson&Johnson (Janssen lub J&J)

4 Sinovac

5 Astra-Zeneca (Oxford, Vaxzevria)

6 COVAXIN

7 Inna (proszę sprecyzować)

8 Nie wiem

Q9 Wezmę zalecaną dawkę przypominającą szczepionki przeciwko COVID-19.

1 Stanowczo się zgadzam

2 Raczej się zgadzam

3 Nie mam pewności/nie mam zdania

4 Raczej się nie zgadzam

5 Stanowczo się nie zgadzam

Q10 Wierzę, że pandemia COVID-19 się skończyła.

1 Stanowczo się zgadzam

2 Raczej się zgadzam

3 Nie mam pewności/nie mam zdania

4 Raczej się nie zgadzam

5 Stanowczo się nie zgadzam

Q11 Jak bardzo ufał Pan/ufała Pani sposobowi, w jaki rząd w Pana/Pani kraju zarządzał pandemią COVID-19?

1 Nie ufałem(-am)

2 Raczej nie ufałem(-am)

3 Nie mam pewności

4 Raczej ufałem(-am)

5 Ufałem(-am)

6 Nie wiem lub nie pamiętam

Q12 Czy opracowanie szczepionek przeciwko COVID-19 wpłynęło na Pana/Pani zaufanie do przemysłu farmaceutycznego?

1 Zwiększyło zaufanie

2 Nie miało wpływu

3 Zmniejszyło zaufanie

4 Nie wiem lub nie pamiętam

Q13 Czy opracowanie szczepionej przeciwko COVID-19 wpłynęło na Pana/Pani zaufanie do nauki ogólnie?

1 Zwiększyło zaufanie

2 Nie miało wpływu

3 Zmniejszyło zaufanie

4 Nie wiem lub nie pamiętam

Q14 Jak bardzo ufa Pan/Pani władzom służby zdrowia, które zalecają przyjmowanie szczepionek przeciwko COVID-19?

1 W ogólne nie ufam

2 W niewielkim stopniu ufam

3 Umiarkowanie ufam

4 Bardzo ufam

5 Nie wiem lub nie pamiętam

Q15 Czy podczas pandemii był Pan zmuszony/była Pani zmuszona do przełożenia lub anulowania jakichkolwiek potrzebnych Panu/Pani usług medycznych niezwiązanych z COVID-19?

1 Tak

2 Nie

3 Nie wiem lub nie pamiętam

Q16 Czy przestrzegałby Pan/przestrzegałaby Pani w przyszłości wymogu (obowiązku) szczepienia wprowadzonego przez następujące organy? Proszę wybrać wszystkie odpowiedzi, które mają zastosowanie:

1 Rząd

2 Pracodawca

3 Szkoła lub uniwersytet

4 Żadne z powyższych

5 Nie wiem

Q17 Czy przestrzegałby Pan/przestrzegałaby Pani w przyszłości wymogu (obowiązku) noszenia maski wprowadzonego przez następujące organy? Proszę wybrać wszystkie odpowiedzi, które mają zastosowanie:

1 Rząd

2 Pracodawca

3 Szkoła lub uniwersytet

4 Żadne z powyższych

5 Nie wiem

Q18 Według Pana/Pani wiedzy czy choruje Pan/Pani lub chorował Pan/chorowała Pani na COVID-19?

1 Tak

2 Nie

3 Nie wiem lub nie pamiętam

Q19 "Długi COVID został zdefiniowany jako „objawy, które mogą trwać przez całe tygodnie lub miesiące po wyzdrowieniu z poważnej choroby”. Według Pana/Pani wiedzy czy choruje Pan/Pani lub chorował Pan/chorowała Pani na długi COVID? "

1 Tak

2 Nie

3 Nie wiem lub nie pamiętam

Q20 Czy przyjmował Pan/przyjmowała Pani whichkolwiek z następujących leków w celu wyleczenia się z COVID-19? Proszę wybrać wszystkie odpowiedzi, które mają zastosowanie:

1 Paxlovid

2 Molnupiravir (Lagevrio)

3 Antyciała monocyklonalne (Olumiant/Baricitinib)

4 Ivermectin

5 Tradycyjna medycyna, wyciągi ziołowe i inne zabiegi

6 Nie wiem lub nie pamiętam

7 Nie, nie przyjmowałem(-am) żadnych lekarstw na COVID-19

Q21 Czy stracił Pan/straciła Pani członka rodziny lub bliskiego przyjaciela z powodu choroby COVID-19? Proszę wybrać wszystkie odpowiedzi, które mają zastosowanie:

1 Tak, w ostatnim roku

2 Tak, ponad rok temu

3 Nie

Q22 Byłem zadowolony/byłam zadowolona z polityki informowania o szczepionkach przeciwko COVID-19 prowadzonej przez władze służby zdrowia w moim kraju.

1 Stanowczo się zgadzam

2 Raczej się zgadzam

3 Nie mam pewności/nie mam zdania/nie pamiętam

4 Raczej się nie zgadzam

5 Stanowczo się nie zgadzam

Q23 Z łatwością odróżniałem(-am) prawdziwe i fałszywe informacje w Internecie na temat szczepienia przeciwko COVID-19.

1 Stanowczo się zgadzam

2 Raczej się zgadzam

3 Nie mam pewności/nie mam zdania/nie pamiętam

4 Raczej się nie zgadzam

5 Stanowczo się nie zgadzam

Q24 Nadal z uwagą śledzę informacje na temat szczepionek przeciwko COVID-19.

1 Stanowczo się zgadzam

2 Raczej się zgadzam

3 Nie mam pewności/nie mam zdania/nie pamiętam

4 Raczej się nie zgadzam

5 Stanowczo się nie zgadzam

Q25 Nadal z uwagą śledzę informacje na temat szczepionek ogólnie.

1 Stanowczo się zgadzam

2 Raczej się zgadzam

3 Nie mam pewności/nie mam zdania

4 Raczej się nie zgadzam

5 Stanowczo się nie zgadzam

Q26 Pandemia COVID-19 sprawiła, że chętniej szczepię się przeciwko innym chorobom (np. grupie, odrze, WZW typu B).

1 Stanowczo się zgadzam

2 Raczej się zgadzam

3 Nie mam pewności/nie mam zdania

4 Raczej się nie zgadzam

5 Stanowczo się nie zgadzam

Q27 Moje doświadczenie ze szczepionką przeciwko COVID-19 miało wpływ na moje zaufanie do innych szczepionek.

1 Zwiększyło zaufanie

2 Nie miało wpływu

3 Zmniejszyło zaufanie

4 Nie wiem

Q28 Informowanie w mediach tradycyjnych o szczepionkach przeciwko COVID-19 miało wpływ na moje zaufanie do innych szczepionek.

1 Zwiększyło zaufanie

2 Nie miało wpływu

3 Zmniejszyło zaufanie

4 Nie wiem

Q29 Informowanie w mediach społecznościowych o szczepionkach przeciwko COVID-19 miało wpływ na moje zaufanie do innych szczepionek.

1 Zwiększyło zaufanie

2 Nie miało wpływu

3 Zmniejszyło zaufanie

4 Nie wiem

Q30 Czy gdyby zatwierdzono szczepionkę mRNA przeciwko chorobie, na którą jest Pan narażony/Pani narażona, przyjęłaby Pan/przyjęłaby Pani ją?

1 Stanowczo nie

2 Nie mam pewności, ale raczej nie

3 Nie mam pewności, ale raczej tak

4 Stanowczo tak

Q31 Czy jeśli Światowa Organizacja Zdrowia (WHO) ogłosiłaby nowe zagrożenie pandemiczne, to uwierzyłby Pan/uwierzyłaby Pani takiej informacji?

1 Tak

2 Nie

3 Nie wiem

Q32 Jeśli Światowa Organizacja Zdrowia (WHO) ogłosiłaby nowe zagrożenie pandemią i zaleciła szczepienia, czy zaszczepiłby się Pan/zaszczepiłaby się Pani?

1 Tak

2 Nie

3 Nie wiem

Q33 "W skali od 1 (w pełni ufam) do 10 (w ogóle nie ufam) jak bardzo ufa Pan/Pani następującym źródłom informacji na temat szczepionek przeciwko COVID-19: Moja rodzina i przyjaciele/znajomi"

Q34 Mój pracodawca

Q35 Mój lekarz lub pielęgniarka

Q36 Mój rząd

Q37 Przywódcy religijni

Q38 Media tradycyjne (np. telewizja, radio, Internet, gazety)

Q39 Media społecznościowe (np. Facebook, Twitter [X], Instagram, WhatsApp, LinkedIn, TikTok)

Q40 Światowa Organizacja Zdrowia (WHO)

Q41 Centra Zwalczania i Zapobiegania Chorobom (Centers for Disease Control and Prevention, CDC), USA

Q42 Europejskie Centrum ds. Zapobiegania i Kontroli Chorób (ECDC)

Q43 Publiczne instytucje służby zdrowia w moim kraju

Q44 Jak bardzo jest Pan przekonany/Pani przekonana, że lepiej poradzimy sobie z kolejnym kryzysem zdrowotnym niż w przypadku pandemii COVID-19?

1 W ogólne nie mam takiej pewności

2 Raczej jestem przekonany(-a)

3 Jestem bardzo przekonany(-a)

4 Nie wiem

Q45 Z tego co Pan przeczytał lub usłyszał/Pani przeczytała lub usłyszała, czy Pana/Pani zdaniem zmiana klimatu/globalne ocieplenie to realne zagrożenie czy nierealne zagrożenie?

1 Realne zagrożenie

2 Nierealne zagrożenie

Q46 Proszę określić swój wiek

Q47 Proszę określić swoją płeć

1 Mężczyzna

2 Kobieta

3 Wolę nie odpowiadać

Q48 Wykształcenie (zależnie od danego kraju)

1 Niepełne średnie wykształcenie

2 Pełne średnie wykształcenie

3 Niepełne wyższe wykształcenie lub dyplom zawodowy

4 Pełne wyższe wykształcenie

5 Wykształcenie podyplomowe

6 Doktorat, habilitacja lub równoważny stopień

Q49 Mediana dochodu (konkretnie dla danego kraju)

1 Tak, mój dochód miesięczny to ponad 6 149 PLN.

2 Tak, mój dochód miesięczny to poniżej 6 149 PLN.

3 Nie, nie mam żadnego dochodu.

4 Odmawiam odpowiedzi/brak odpowiedzi

Q50 Regiony (konkretnie dla danego kraju)

1 Wielkopolskie

2 Kujawsko-pomorskie

3 Małopolskie

4 Łódzkie

5 Dolnośląskie

6	Lubelskie
7	Lubuskie
8	Mazowieckie
9	Opolskie
10	Podlaskie
11	Pomorskie
12	Śląskie
13	Podkarpackie
14	Świętokrzyskie
15	Warmińsko-mazurskie
16	Zachodniopomorskie

Russia

COVID-VAC: Глобальный опрос на тему восприятия вакцины от COVID-19

Цель этого опроса заключается в том, чтобы измерить реакции резидентов на усилия по борьбе с распространением COVID-19. Риски участия в этом текущем опросе рассматриваются как минимальные. Вы можете почувствовать себя некомфортно с учетом потенциальных последствий COVID-19, участие в этом экспериментальном исследовании является полностью добровольным, и Вы можете отказаться от участия в нем в любое время. Ваши ответы останутся анонимными и конфиденциальными. Участие в опросе займет менее 5 минут. Отвечая на вопросы, Вы подтверждаете, что Вы прочитали описание исследования, что Вам более 18 лет и что Вы соглашаетесь с описанными условиями.

Если у Вас есть вопросы о Ваших правах в качестве участника исследования, Вы можете обратиться в экспертный совет медицинского учреждения Emerson College (колледж Эмерсон), который занимается защитой волонтеров, принимающих участие в исследовательских проектах. Вы можете связаться с ведущим исследователем по адресу электронной почты emersonpolling@emerson.edu или обратиться на кафедру экспертного совета медицинского учреждения по адресу электронной почты human_subjects@emerson.edu.

Q1 "Вначале укажите, насколько Вы согласны со следующим утверждением: COVID-19 остается опасной угрозой для здоровья."

1 Полностью согласен(-на)

2 Скорее согласен(-на)

3 Не уверен(-а) / не знаю

4 Скорее не согласен(-на)

5 Категорически не согласен(-на)

Q2 "Теперь мы зададим несколько вопросов, связанных с вакцинацией от COVID-19. Covid-19 можно предотвратить с помощью вакцинации."

1 Полностью согласен(-на)

2 Скорее согласен(-на)

3 Не уверен(-а) / не знаю

4 Скорее не согласен(-на)

5 Категорически не согласен(-на)

Q3 Риски заболевания COVID-19 выше, чем риски, связанные с побочными эффектами от вакцины.

1 Полностью согласен(-на)

2 Скорее согласен(-на)

3 Не уверен(-а) / не знаю

4 Скорее не согласен(-на)

5 Категорически не согласен(-на)

Q4 Доступные мне вакцины от COVID-19 являются безопасными.

1 Полностью согласен(-на)

2 Скорее согласен(-на)

3 Не уверен(-а) / не знаю

4 Скорее не согласен(-на)

5 Категорически не согласен(-на)

Q5 Я доверяю исследованиям, на основе которых разработаны доступные мне вакцины от COVID-19.

1 Полностью согласен(-на)

2 Скорее согласен(-на)

3 Не уверен(-а) / не знаю

4 Скорее не согласен(-на)

5 Категорически не согласен(-на)

Q6 Доступные мне вакцины от COVID-19 являются эффективными для защиты от тяжелой формы COVID-19.

1 Полностью согласен(-на)

2 Скорее согласен(-на)

3 Не уверен(-а) / не знаю

4 Скорее не согласен(-на)

5 Категорически не согласен(-на)

Q7 Вы получили хотя бы одну дозу вакцины от COVID-19?

1 Да, я получил(-а) одну дозу

2 Да, я получил(-а) две или больше дозы

3 Нет

Q8 Какую из следующих вакцин от COVID-19 Вы получили? Выберите все подходящие варианты:

1 Moderna (Spikevax)

2 Pfizer (Comirnaty)

3 Johnson&Johnson (Janssen or J&J)

4 Sinovac

5 Astra-Zeneca (Oxford, Vaxzevria)

6 COVAXIN

7 Другое (укажите)

8 Не знаю

Q9 Я получу рекомендуемую бустерную дозу вакцины от COVID-19.

1 Полностью согласен(-на)

2 Скорее согласен(-на)

3 Не уверен(-а) / не знаю

4 Скорее не согласен(-на)

5 Категорически не согласен(-на)

Q10 Я считаю, что пандемия Covid-19 закончилась.

1 Полностью согласен(-на)

2 Скорее согласен(-на)

3 Не уверен(-а) / не знаю

4 Скорее не согласен(-на)

5 Категорически не согласен(-на)

Q11 Насколько Вы доверяли правительству в борьбе с пандемией COVID-19 в Вашей стране?

1 Не доверял(-а)

2 В целом не доверял(-а)

3 Нейтральное мнение

4 В целом доверял

5 Доверял(-а)

6 Не знаю или не помню

Q12 Повлияла ли разработка вакцины от COVID-19 на уровень Вашего доверия к фармацевтической промышленности?

1 Уровень доверия увеличился

2 Без изменений

3 Уровень доверия снизился

4 Не знаю или не помню

Q13 Повлияла ли разработка вакцины от COVID-19 на уровень Вашего доверия к науке в целом?

1 Уровень доверия увеличился

2 Без изменений

3 Уровень доверия снизился

4 Не знаю или не помню

Q14 Насколько Вы доверяете органам здравоохранения, которые рекомендовали Вам сделать вакцину от COVID-19?

1 Совсем не доверяю

2 Небольшой уровень доверия

3 Средний уровень доверия

4 Полностью доверяю

5 Не знаю или не помню

Q15 Приходилось ли Вам во время пандемии откладывать или отменять какие-либо необходимые Вам медицинские услуги, которые не были связаны с COVID-19?

1 Да

2 Нет

3 Не знаю или не помню

Q16 Вы будете в будущем выполнять (обязательные) требования, связанные с вакцинацией, полученные от следующих органов или лиц? Выберите все подходящие варианты:

1 Правительство

2 Работодатель

3 Школа или университет

4 Ни один из перечисленных выше вариантов

5 Не знаю

Q17 Вы будете в будущем выполнять (обязательные) требования, связанные с ношением маски, полученные от следующих органов или лиц? Выберите все подходящие варианты:

1 Правительство

2 Работодатель

3 Школа или университет

4 Ни один из перечисленных выше вариантов

5 Не знаю

Q18 Насколько Вам известно, Вы болеете в настоящее время или болели в прошлом на COVID-19?

1 Да

2 Нет

3 Не знаю или не помню

Q19 "«Длительный ковид» определяется как «симптомы, которые могут сохраняться в течение недель или месяцев после выздоровления после острого заболевания». Насколько Вам известно, у Вас был «длительный ковид»? "

1 Да

2 Нет

3 Не знаю или не помню

Q20 Вы принимали какие-либо из следующих лекарств для лечения COVID-19? Выберите все подходящие варианты:

1 Paxlovid

2 Molnupiravir (Lagevrio)

3 Моноклональные антитела (Olumiant/Baricitinib)

4 Ivermectin

5 Лекарственные средства традиционной медицины, экстракты трав и траволечение

6 Не знаю или не помню

7 Нет, я не принимал(-а) лекарства от COVID-19.

Q21 Вы потеряли члена семьи или близкого друга из-за болезни COVID-19? Выберите все подходящие варианты:

1 Да, за последний год

2 Да, больше года назад

3 Нет

Q22 Я был(-а) удовлетворен(-а) усилиями, которые органы здравоохранения моей страны прилагали по информированию о вакцинах от COVID-19.

1 Полностью согласен(-на)

2 Скорее согласен(-на)

3 Не уверен(-а) / нет мнения / не помню

4 Скорее не согласен(-на)

5 Категорически не согласен(-на)

Q23 Я легко могу понять разницу между точной и ложной информацией о вакцине от COVID-19 в интернете.

1 Полностью согласен(-на)

2 Скорее согласен(-на)

3 Не уверен(-а) / нет мнения / не помню

4 Скорее не согласен(-на)

5 Категорически не согласен(-на)

Q24 Я продолжаю обращать внимание на информацию о вакцинах от COVID-19.

1 Полностью согласен(-на)

2 Скорее согласен(-на)

3 Не уверен(-а) / нет мнения / не помню

4 Скорее не согласен(-на)

5 Категорически не согласен(-на)

Q25 Я продолжаю обращать внимание на информацию о вакцинах в целом.

1 Полностью согласен(-на)

2 Скорее согласен(-на)

3 Не уверен(-а) / не знаю

4 Скорее не согласен(-на)

5 Категорически не согласен(-на)

Q26 Пандемия COVID-19 побудила меня сделать вакцинацию от других заболеваний (например, гриппа, кори, вирусного гепатита B).

1 Полностью согласен(-на)

2 Скорее согласен(-на)

3 Не уверен(-а) / не знаю

4 Скорее не согласен(-на)

5 Категорически не согласен(-на)

Q27 Мой опыт применения вакцины от COVID-19 повлиял на мое доверие к другим вакцинам.

1 Уровень доверия увеличился

2 Без изменений

3 Уровень доверия снизился

4 Не знаю

Q28 Освещение в средствах массовой информации вакцин от COVID-19 повлияло на мое доверие к другим вакцинам.

1 Уровень доверия увеличился

2 Без изменений

3 Уровень доверия снизился

4 Не знаю

Q29 Информация в социальных сетях о вакцинах от COVID-19 повлияла на мое доверие к другим вакцинам.

1 Уровень доверия увеличился

2 Без изменений

3 Уровень доверия снизился

4 Не знаю

Q30 Если бы мРНК-вакцина была одобрена для лечения заболевания, которым Вы можете заболеть, Вы бы сделали вакцинацию?

1 Определенно нет

2 Не уверен(-а), но скорее нет

3 Не уверен(-а), но скорее да

4 Определенно да

Q31 Если бы Всемирная организация здравоохранения (ВОЗ) объявила о новой угрозе пандемии, Вы бы поверили этой информации?

1 Да

2 Нет

3 Не знаю

Q32 Если бы Всемирная организация здравоохранения (ВОЗ) объявила о новой угрозе пандемии и посоветовала сделать вакцинацию, Вы бы сделали ее?

1 Да

2 Нет

3 Не знаю

Q33 "По шкале от 1 (полностью доверяю) до 10 (совсем не доверяю), насколько Вы доверяете следующим источникам информации о вакцинах от COVID-19: Члены моей семьи и друзья"

Q34 Мой работодатель

Q35 Мой врач или медсестра

Q36 Мое правительство

Q37 Религиозные лидеры

Q38 Средства массовой информации (например, телевидение, интернет, радио, газеты)

Q39 Социальные сети (например, Facebook, Twitter [X], Instagram, WhatsApp, LinkedIn, TikTok)

Q40 Всемирная организация здравоохранения (ВОЗ)

Q41 Центры по контролю и профилактике заболеваний (CDC), США

Q42 Европейский центр профилактики и контроля заболеваний (ECDC)

Q43 Органы общественного здравоохранения в моей стране

Q44 Насколько Вы уверены, что мы справимся со следующим кризисом в сфере здравоохранения лучше, чем с пандемией COVID-19?

1 Совсем не уверен(-а)

2 Скорее уверен(-а)

3 Полностью уверен(-а)

4 Не знаю

Q45 Судя по тому, что Вы прочитали или услышали, Вы считаете, что изменение климата / глобальное потепление представляет собой реальную угрозу или нет?

1 Представляет реальную угрозу

2 Не представляет реальной угрозы

Q46 Сколько Вам лет?

Q47 Укажите Ваш пол.

1 Мужской

2 Женский

3 Предпочитаю не указывать

Q48 Образование (зависит от страны)

1 Начальное

2 Неполное среднее

3 Старшая средняя школа

4 Техническое или профессиональное образование

5 Степень бакалавра

6 Степень магистра

7 Докторантура

Q49 Средний уровень дохода (в зависимости от страны)

1 Да, мой ежемесячный доход составляет более 96 654 российских рубля.

2 Да, мой ежемесячный доход составляет менее 96 654 российских рублей.

3 Нет, у меня нет дохода.

4 Отказываюсь отвечать / нет ответа

Q50 Регионы (зависит от страны)

1 Центральная Европа

2 Северная и Северо-Западная Европа

3 Поволжье

4 Северный Кавказ

5 Урал

6 Западная Сибирь

7 Восточная Сибирь

8 Северная и Северо-Западная сибирь

9 Дальний Восток России

South Africa

COVID-VAC: A global survey of COVID-19 vaccine perceptions

The purpose of the survey is to measure resident's reactions to COVID-19 response efforts. The risks in this routine survey are considered minimal. You may feel uncomfortable considering the implications of COVID-19 and participation in this research study is completely voluntary and you may discontinue participation at any time. Your responses will remain anonymous and confidential. The survey should take less than 5 minutes to complete. By answering the questions, you are indicating that you have read the description of the study, are over the age of 18, and that you agree to the terms as described.

If you have questions about your rights as a research participant, you may contact the Emerson College Institutional Review Board (IRB), which is concerned with the protection of volunteers in research projects. You may reach the lead investigator via email at emersonpolling@emerson.edu or the Chair of the IRB by e-mailing human_subjects@emerson.edu.

Q1 "First, please indicate your level of agreement with this statement: COVID-19 remains a dangerous health threat."

1 Strongly agree

2 Somewhat agree

3 Unsure/no opinion

4 Somewhat disagree

5 Strongly disagree

Q2 "Now, here are some questions related to COVID-19 vaccines. COVID-19 can be prevented by vaccination."

1 Strongly agree

2 Somewhat agree

3 Unsure/no opinion

4 Somewhat disagree

5 Strongly disagree

Q3 The risks of COVID-19 disease are greater than the risks of the vaccine.

1 Strongly agree

2 Somewhat agree

3 Unsure/no opinion

4 Somewhat disagree

5 Strongly disagree

Q4 The COVID-19 vaccines available to me are safe.

1 Strongly agree

2 Somewhat agree

3 Unsure/no opinion

4 Somewhat disagree

5 Strongly disagree

Q5 I trust the science behind the COVID-19 vaccines available to me.

1 Strongly agree

2 Somewhat agree

3 Unsure/no opinion

4 Somewhat disagree

5 Strongly disagree

Q6 The COVID-19 vaccines available to me are effective in protecting against severe COVID-19.

1 Strongly agree

2 Somewhat agree

3 Unsure/no opinion

4 Somewhat disagree

5 Strongly disagree

Q7 Have you received at least one dose of a COVID-19 vaccine?

1 Yes, I received one dose

2 Yes, I received two or more doses

3 No

Q8 Which of the following COVID-19 vaccines did you receive? Select all that apply:

1 Moderna (Spikevax)

2 Pfizer (Comirnaty)

3 Johnson&Johnson (Janssen or J&J)

4 Sinovac

5 Astra-Zeneca (Oxford, Vaxzevria)

6 COVAXIN

7 Other (please specify)

8 Don’t know

Q9 I will take the recommended COVID-19 booster.

1 Strongly agree

2 Somewhat agree

3 Unsure/no opinion

4 Somewhat disagree

5 Strongly disagree

Q10 I believe the COVID-19 pandemic is over.

1 Strongly agree

2 Somewhat agree

3 Unsure/no opinion

4 Somewhat disagree

5 Strongly disagree

Q11 How much did you trust your government's management of the COVID-19 pandemic in your country?

1 Did not trust

2 Somewhat did not trust

3 Unsure

4 Somewhat trust

5 Trust

6 Don't know or don't remember

Q12 Did the development of the COVID-19 vaccines affect your trust in the pharmaceutical industry?

1 Increased trust

2 No effect

3 Decreased trust

4 Don't know or don't remember

Q13 Did the development of COVID-19 vaccines affect your trust in science generally?

1 Increased trust

2 No effect

3 Decreased trust

4 Don't know or don't remember

Q14 How much do you trust the health authorities that recommended you get a COVID-19 vaccine?

1 Not at all

2 A little

3 Moderately

4 Very much

5 Don’t know or don’t remember

Q15 During the pandemic, did you have to delay or cancel any medical services that you needed unrelated to COVID-19?

1 Yes

2 No

3 Don’t know or don’t remember

Q16 Would you follow a vaccine requirement (mandate) in the future from any of the following? Select all that apply:

1 Government

2 Employer

3 School or university

4 None of the above

5 Don’t know

Q17 Would you follow a face mask requirement (mandate) in the future from any of the following? Select all that apply:

1 Government

2 Employer

3 School or university

4 None of the above

5 Don’t know

Q18 To your knowledge, do you have or have you had COVID-19?

1 Yes

2 No

3 Don’t know or don’t remember

Q19 "Long-COVID has been defined as 'symptoms that can last for weeks or months after recovery from acute illness'. To your knowledge, do you have or have you had Long COVID? "

1 Yes

2 No

3 Don’t know or don’t remember

Q20 Did you take any of the following medicines to treat COVID-19? Select all that apply:

1 Paxlovid

2 Molnupiravir (Lagevrio)

3 Monoclonal antibodies (Olumiant/Baricitinib)

4 Ivermectin

5 Traditional medicine, herbal extracts and treatments

6 Don’t know or don’t remember

7 No, I did not take medicines for COVID-19

Q21 Have you lost a family member or close friend to COVID-19 disease? Select all that apply:

1 Yes, within the past year

2 Yes, more than a year ago

3 No

Q22 I was satisfied with the communication efforts on COVID-19 vaccines made by health authorities in my country.

1 Strongly agree

2 Somewhat agree

3 Unsure/no opinion/don’t remember

4 Somewhat disagree

5 Strongly disagree

Q23 It is easy for me to know the difference between accurate and false information about the COVID-19 vaccine on the Internet.

1 Strongly agree

2 Somewhat agree

3 Unsure/no opinion/don’t remember

4 Somewhat disagree

5 Strongly disagree

Q24 I continue to pay attention to information on COVID-19 vaccines.

1 Strongly agree

2 Somewhat agree

3 Unsure/no opinion/don't remember

4 Somewhat disagree

5 Strongly disagree

Q25 I continue to pay attention to information on vaccines in general.

1 Strongly agree

2 Somewhat agree

3 Unsure/no opinion

4 Somewhat disagree

5 Strongly disagree

Q26 The COVID-19 pandemic has made me more willing to get vaccinated against other diseases (e.g., flu, measles, viral hepatitis B).

1 Strongly agree

2 Somewhat agree

3 Unsure/no opinion

4 Somewhat disagree

5 Strongly disagree

Q27 My experience with the COVID-19 vaccine has affected my trust in other vaccines.

1 Increased trust

2 No effect

3 Decreased trust

4 Don't know

Q28 Broadcast media coverage on COVID-19 vaccines has affected my trust in other vaccines.

1 Increased trust

2 No effect

3 Decreased trust

4 Don’t know

Q29 Social media coverage on COVID-19 vaccines has affected my trust in other vaccines.

1 Increased trust

2 No effect

3 Decreased trust

4 Don’t know

Q30 If an mRNA vaccine is approved for a disease for which you are at risk, would you take it?

1 Definitely no

2 Unsure, but leaning towards no

3 Unsure, but leaning towards yes

4 Definitely yes

Q31 If the World Health Organization (WHO) announced a new pandemic threat, would you trust this information?

1 Yes

2 No

3 Don’t know

Q32 If the World Health Organization (WHO) announced a new pandemic threat and advised getting vaccinated, would you?

1 Yes

2 No

3 Don’t know

Q33 "On a scale of 1 (trust completely) to 10 (do not trust at all) how much do you trust the following sources of information about COVID-19 vaccines: My family and friends"

Q34 My employer

Q35 My doctor or nurse

Q36 My government

Q37 Religious leaders

Q38 News media (e.g., television, Internet, radio, newspapers)

Q39 Social media (e.g., Facebook, Twitter [X], Instagram, WhatsApp, LinkedIn, TikTok)

Q40 World Health Organization (WHO)

Q41 Centers for Disease Control and Prevention (CDC), USA

Q42 European Centre for Disease Prevention and Control (ECDC)

Q43 The public health authorities in my country

Q44 How confident are you that we will manage the next health crisis better than the COVID-19 pandemic?

1 Not at all confident

2 Somewhat confident

3 Very confident

4 Don’t know

Q45 From what you have read or heard, do you think climate change/global warming is a real threat or not a real threat?

1 Real threat

2 Not a real threat

Q46 What is your age?

Q47 What is your gender?

1 Male

2 Female

3 Prefer not to say

Q48 Education (country specific)

1 Grade Ten (Standard Eight)

2 Grade Four to Grade Nine (Standard Two to Standard Seven)

3 Grade Eleven (Standard Nine)

4 Grade Twelve (Standard Ten / Matric)

5 National Certificate National Diploma

6 Trade Certificate

7 Bachelors Degree (Three Years)

8 Occupational Certificate

9 Higher Diploma

10 Honours Degree

11 Post Graduate Certificate

12 Doctorate

13 Masters

Q49 Median Income Level (country specific)

1 Yes, my monthly income is more than 10,094 South African Rand.

2 Yes, my monthly income is less than 10,094 South African Rand.

3 No, I do not have an income.

4 Refused/ Did not answer

Q50 Regions (country specific)

1 Western Cape

2 Eastern Cape

3 Northern Cape

4 North West

5 Free State

6 KwaZulu-Natal

7 Gauteng

8 Limpopo

9 Mpumalanga

South Korea

코로나 백신: 코로나19 인식에 대한 글로벌 설문조사

본 설문조사의 목적은 코로나19 대응 노력에 대한 거주민 여러분의 반응을 측정하기 위한 것입니다. 본 정기 설문조사의 위험도는 낮은 편입니다. 코로나19의 여파를 고려할 때 본 설문조사가 불편하게 느껴지실 수도 있으나, 전적으로 자발적으로 이뤄지는 본 설문조사는 진행 도중 언제든지 중단하실 수 있습니다. 귀하의 답변은 익명이 보장되며 기밀로 처리됩니다. 본 설문조사는 완료까지 5분도 걸리지 않습니다. 질문에 답함으로써 귀하는 본 설문조사에 대한 설명을 확인했으며, 자신의 연령이 만 18세보다 많으며, 설명된 조건에 동의함을 확인합니다.

설문조사 참여자로서 자신의 권리에 대해 질문이 있을 경우, IRB(Emerson College Institutional Review Board)에 문의하실 수 있습니다. IRB는 연구 프로젝트 자원 참여자의 보호를 관할하는 기구입니다. 귀하는 emersonpolling@emerson.edu로 선임 조사관에게 이메일로 문의하시거나 human_subjects@emerson.edu로 IRB 의장에게 이메일로 문의하실 수 있습니다.

Q1 "먼저 다음 진술에 얼마나 동의하시는지 알려주세요. 코로나19는 여전히 건강을 위협하는 위험요인이다."

1 매우 동의

2 다소 동의

3 중립/의견 없음

4 다소 동의하지 않음

5 전혀 동의하지 않음

Q2 "이제 코로나19 백신과 관련하여 몇 가지 질문을 드리겠습니다. 코로나19는 백신으로 예방할 수 있다."

1 매우 동의

2 다소 동의

3 중립/의견 없음

4 다소 동의하지 않음

5 전혀 동의하지 않음

Q3 코로나19의 질병 위험은 백신 위험보다 더 크다.

1 매우 동의

2 다소 동의

3 중립/의견 없음

4 다소 동의하지 않음

5 전혀 동의하지 않음

Q4 내가 이용할 수 있는 코로나19 백신은 안전하다.

1 매우 동의

2 다소 동의

3 중립/의견 없음

4 다소 동의하지 않음

5 전혀 동의하지 않음

Q5 내가 이용할 수 있는 코로나19 백신을 뒷받침하는 과학을 신뢰한다.

1 매우 동의

2 다소 동의

3 중립/의견 없음

4 다소 동의하지 않음

5 전혀 동의하지 않음

Q6 내가 이용할 수 있는 코로나19 백신은 중증 코로나19를 예방하는 데 효과적이다.

1 매우 동의

2 다소 동의

3 중립/의견 없음

4 다소 동의하지 않음

5 전혀 동의하지 않음

Q7 코로나19 백신을 최소 1회 이상 접종하셨습니까?

1 예, 1회 접종했습니다

2 예, 2회 이상 접종했습니다

3 아니요

Q8 다음 중 귀하가 접종하신 코로나19 백신은 무엇입니까? 해당 사항을 모두 고르세요.

1 모더나(스파이크백스)

2 화이자(코미나티)

3 존슨앤존슨(얀센 또는 J&J)

4 시노백

5 아스트라제네카(옥스포드, 백스제브리아)

6 코백신

7 기타(자세히 알려 주세요)

8 모르겠음

Q9 나는 추천받은 코로나19 부스터를 맞겠다.

1 매우 동의

2 다소 동의

3 중립/의견 없음

4 다소 동의하지 않음

5 전혀 동의하지 않음

Q10 나는 코로나19 팬데믹이 끝났다고 생각한다.

1 매우 동의

2 다소 동의

3 중립/의견 없음

4 다소 동의하지 않음

5 전혀 동의하지 않음

Q11 자국의 코로나19 팬데믹에 대한 정부의 대처를 얼마나 신뢰하셨습니까?

1 신뢰하지 않음

2 다소 신뢰하지 않음

3 중립

4 다소 신뢰

5 신뢰

6 모르겠음/기억 안 남

Q12 코로나19 백신 개발이 제약업계에 대한 귀하의 신뢰에 영향을 미쳤습니까?

1 더 신뢰하게 됨

2 영향 없음

3 덜 신뢰하게 됨

4 모르겠음/기억 안 남

Q13 코로나19 백신 개발이 과학 전반에 대한 귀하의 신뢰에 영향을 미쳤습니까?

1 더 신뢰하게 됨

2 영향 없음

3 덜 신뢰하게 됨

4 모르겠음/기억 안 남

Q14 코로나19 백신 접종을 권고한 보건당국을 얼마나 신뢰하십니까?

1 전혀 하지 않음

2 다소 신뢰

3 중간 정도 신뢰

4 매우 많이 신뢰

5 모르겠음/기억 안 남

Q15 팬데믹 동안 코로나19와 관련 없이 필요한 의료 서비스를 미루거나 취소해야 했습니까?

1 예

2 아니요

3 모르겠음/기억 안 남

Q16 귀하가 앞으로 백신 조건(의무사항)을 준수할 의향이 있는 곳은 어디입니까? 해당 사항을 모두 고르세요.

1 정부

2 고용주

3 학교/대학교

4 해당 없음

5 모르겠음

Q17 귀하가 향후 얼굴마스크 조건(의무사항)을 준수할 의향이 있는 곳은 어디입니까? 해당 사항을 모두 고르세요.

1 정부

2 고용주

3 학교/대학교

4 해당 없음

5 모르겠음

Q18 귀하의 생각에 귀하는 코로나19에 걸렸거나 앓은 적이 있습니까?

1 예

2 아니요

3 모르겠음/기억 안 남

Q19 "만성 코로나는 '급성 질환에서 회복한 후 수주 또는 수개월 동안 지속되는 증상'으로 정의되었습니다. 귀하의 생각에 귀하는 만성 코로나에 걸렸거나 앓은 적이 있습니까? "

1 예

2 아니요

3 모르겠음/기억 안 남

Q20 다음 중 코로나19 치료를 위해 드신 약은 무엇입니까? 해당 사항을 모두 고르세요.

1 팍스로비드

2 몰누피라비르(라게브리오)

3 단일클론항체(올루미언트/바리시티닙)

4 이버멕틴

5 전통의약, 한약추출물, 한방

6 모르겠음/기억 안 남

7 아니요, 코로나19 약을 먹지 않음

Q21 코로나19로 귀하의 가족이나 가까운 친구 중 세상을 떠난 분이 계십니까? 해당 사항을 모두 고르세요.

1 예, 작년에 있었음

2 예, 1년 이상 전에 있었음

3 아니요

Q22 나는 코로나19 백신에 대해 우리나라 보건당국이 소통하고자 노력했던 것이 만족스러웠다.

1 매우 동의

2 다소 동의

3 중립/의견 없음/기억 안 남

4 다소 동의하지 않음

5 전혀 동의하지 않음

Q23 나는 인터넷에서 코로나19 백신에 대해 정확한 정보와 허위 정보가 다른 점을 쉽게 알 수 있다.

1 매우 동의

2 다소 동의

3 중립/의견 없음/기억 안 남

4 다소 동의하지 않음

5 매우 동의하지 않음

Q24 나는 코로나19 백신에 대한 정보에 지속적으로 관심을 기울이고 있다.

1 매우 동의

2 다소 동의

3 중립/의견 없음/기억 안 남

4 다소 동의하지 않음

5 매우 동의하지 않음

Q25 나는 백신 전반에 대한 정보에 지속적으로 관심을 기울이고 있다.

1 매우 동의

2 다소 동의

3 중립/의견 없음

4 다소 동의하지 않음

5 전혀 동의하지 않음

Q26 나는 코로나19 팬데믹으로 다른 질병(예: 독감, 홍역, 바이러스성 B형 간염)의 예방접종을 받고 싶은 마음이 더 커졌다.

1 매우 동의

2 다소 동의

3 중립/의견 없음

4 다소 동의하지 않음

5 매우 동의하지 않음

Q27 코로나19 백신 경험이 다른 백신에 대한 신뢰에 영향을 미쳤다.

1 더 신뢰하게 됨

2 영향 없음

3 덜 신뢰하게 됨

4 모르겠음

Q28 코로나19 백신에 대한 방송매체의 보도가 다른 백신에 대한 신뢰에 영향을 미쳤다.

1 더 신뢰하게 됨

2 영향 없음

3 덜 신뢰하게 됨

4 모르겠음

Q29 코로나19 백신에 대한 소셜미디어의 게시물이 다른 백신에 대한 신뢰에 영향을 미쳤다.

1 더 신뢰하게 됨

2 영향 없음

3 덜 신뢰하게 됨

4 모르겠음

Q30 귀하가 위중한 상태에 있는 병에 대해 mRNA 백신이 승인되어 있다면 이 백신을 맞으시겠습니까?

1 절대 안 맞겠음

2 중립, 안 맞을 가능성이 조금 더 높음

3 중립, 맞을 가능성이 조금 더 높음

4 꼭 맞겠음

Q31 세계보건기구(WHO)가 새로운 전염병의 위협을 발표한다면, 이 정보를 신뢰하시겠습니까?

1 예

2 아니요

3 모르겠음

Q32 세계보건기구(WHO)가 새로운 팬데믹 위협을 발표하고 백신 접종을 권고한다면, 귀하는 그렇게 하시겠습니까?

1 예

2 아니요

3 모르겠음

Q33 "1-10척도로 다음 정보출처에 대한 귀하의 신뢰도를 평가해주세요. 1=완전 신뢰, 10=전혀 신뢰하지 않음을 의미합니다 코로나19 백신: 가족이나 친구"

Q34 고용주

Q35 의사나 간호사

Q36 정부

Q37 종교 리더

Q38 뉴스매체(예: TV, 인터넷, 라디오, 신문)

Q39 소셜미디어(예: 페이스북, 트위터[X], 인스타그램, 왓츠앱, 링크드인, 틱톡)

Q40 세계보건기구(WHO)

Q41 미국 질병통제예방센터(CDC)

Q42 유럽 질병예방통제센터(ECDC)

Q43 우리나라 보건당국

Q44 우리가 다음 번 보건 위기를 코로나19 팬데믹보다 더 잘 관리할 거라고 얼마나 확신하십니까?

1 전혀 확신하지 않음

2 다소 확신

3 매우 확신

4 모르겠음

Q45 귀하가 읽거나 들은 바를 기준으로 귀하는 기후변화/지구온난화가 진짜 위협이라고 생각하시나요 아니면 진짜 위험이 아니라고 생각하십니까?

1 진짜 위협이다

2 진짜 위협이 아니다

Q46 나이가 어떻게 되시나요?

Q47 성별을 알려주시겠습니까?

1 남

2 여

3 답하고 싶지 않음

Q48 교육(국가별)

1 초등학교

2 중등학교

3 고등학교

4 학사 학위

5 석사 과정

6 박사 과정

Q49 중위소득 수준(국가별)

1 예, 제 월수입은 한화로 3,839,830원을 넘습니다.

2 예, 제 월수입은 한화로 3,839,830원이 안됩니다.

3 아니요, 월수입이 없습니다.

4 응답 거부/답하지 않음

Q50 지역(국가별)

1 충북

2 충남

3 강원

4 경기

5 경북

6 경남

7 전북

8 전남

9 제주

Singapore

COVID-VAC: A global survey of COVID-19 vaccine perceptions

The purpose of the survey is to measure resident's reactions to COVID-19 response efforts. The risks in this routine survey are considered minimal. You may feel uncomfortable considering the implications of COVID-19 and participation in this research study is completely voluntary and you may discontinue participation at any time. Your responses will remain anonymous and confidential. The survey should take less than 5 minutes to complete. By answering the questions, you are indicating that you have read the description of the study, are over the age of 18, and that you agree to the terms as described.

If you have questions about your rights as a research participant, you may contact the Emerson College Institutional Review Board (IRB), which is concerned with the protection of volunteers in research projects. You may reach the lead investigator via email at emersonpolling@emerson.edu or the Chair of the IRB by e-mailing human_subjects@emerson.edu.

Q1 "First, please indicate your level of agreement with this statement: COVID-19 remains a dangerous health threat."

1 Strongly agree

2 Somewhat agree

3 Unsure/no opinion

4 Somewhat disagree

5 Strongly disagree

Q2 "Now, here are some questions related to COVID-19 vaccines. COVID-19 can be prevented by vaccination."

1 Strongly agree

2 Somewhat agree

3 Unsure/no opinion

4 Somewhat disagree

5 Strongly disagree

Q3 The risks of COVID-19 disease are greater than the risks of the vaccine.

1 Strongly agree

2 Somewhat agree

3 Unsure/no opinion

4 Somewhat disagree

5 Strongly disagree

Q4 The COVID-19 vaccines available to me are safe.

1 Strongly agree

2 Somewhat agree

3 Unsure/no opinion

4 Somewhat disagree

5 Strongly disagree

Q5 I trust the science behind the COVID-19 vaccines available to me.

1 Strongly agree

2 Somewhat agree

3 Unsure/no opinion

4 Somewhat disagree

5 Strongly disagree

Q6 The COVID-19 vaccines available to me are effective in protecting against severe COVID-19.

1 Strongly agree

2 Somewhat agree

3 Unsure/no opinion

4 Somewhat disagree

5 Strongly disagree

Q7 Have you received at least one dose of a COVID-19 vaccine?

1 Yes, I received one dose

2 Yes, I received two or more doses

3 No

Q8 Which of the following COVID-19 vaccines did you receive? Select all that apply:

1 Moderna (Spikevax)

2 Pfizer (Comirnaty)

3 Johnson&Johnson (Janssen or J&J)

4 Sinovac

5 Astra-Zeneca (Oxford, Vaxzevria)

6 COVAXIN

7 Other (please specify)

8 Don't know

Q9 I will take the recommended COVID-19 booster.

1 Strongly agree

2 Somewhat agree

3 Unsure/no opinion

4 Somewhat disagree

5 Strongly disagree

Q10 I believe the COVID-19 pandemic is over.

1 Strongly agree

2 Somewhat agree

3 Unsure/no opinion

4 Somewhat disagree

5 Strongly disagree

Q11 How much did you trust your government's management of the COVID-19 pandemic in your country?

1 Did not trust

2 Somewhat did not trust

3 Unsure

4 Somewhat trust

5 Trust

6 Don't know or don't remember

Q12 Did the development of the COVID-19 vaccines affect your trust in the pharmaceutical industry?

1 Increased trust

2 No effect

3 Decreased trust

4 Don't know or don't remember

Q13 Did the development of COVID-19 vaccines affect your trust in science generally?

1 Increased trust

2 No effect

3 Decreased trust

4 Don't know or don't remember

Q14 How much do you trust the health authorities that recommended you get a COVID-19 vaccine?

1 Not at all

2 A little

3 Moderately

4 Very much

5 Don’t know or don’t remember

Q15 During the pandemic, did you have to delay or cancel any medical services that you needed unrelated to COVID-19?

1 Yes

2 No

3 Don’t know or don’t remember

Q16 Would you follow a vaccine requirement (mandate) in the future from any of the following? Select all that apply:

1 Government

2 Employer

3 School or university

4 None of the above

5 Don’t know

Q17 Would you follow a face mask requirement (mandate) in the future from any of the following? Select all that apply:

1 Government

2 Employer

3 School or university

4 None of the above

5 Don’t know

Q18 To your knowledge, do you have or have you had COVID-19?

1 Yes

2 No

3 Don’t know or don’t remember

Q19 "Long-COVID has been defined as 'symptoms that can last for weeks or months after recovery from acute illness'. To your knowledge, do you have or have you had Long COVID? "

1 Yes

2 No

3 Don’t know or don’t remember

Q20 Did you take any of the following medicines to treat COVID-19? Select all that apply:

1 Paxlovid

2 Molnupiravir (Lagevrio)

3 Monoclonal antibodies (Olumiant/Baricitinib)

4 Ivermectin

5 Traditional medicine, herbal extracts and treatments

6 Don’t know or don’t remember

7 No, I did not take medicines for COVID-19

Q21 Have you lost a family member or close friend to COVID-19 disease? Select all that apply:

1 Yes, within the past year

2 Yes, more than a year ago

3 No

Q22 I was satisfied with the communication efforts on COVID-19 vaccines made by health authorities in my country.

1 Strongly agree

2 Somewhat agree

3 Unsure/no opinion/don’t remember

4 Somewhat disagree

5 Strongly disagree

Q23 It is easy for me to know the difference between accurate and false information about the COVID-19 vaccine on the Internet.

1 Strongly agree

2 Somewhat agree

3 Unsure/no opinion/don’t remember

4 Somewhat disagree

5 Strongly disagree

Q24 I continue to pay attention to information on COVID-19 vaccines.

1 Strongly agree

2 Somewhat agree

3 Unsure/no opinion/don't remember

4 Somewhat disagree

5 Strongly disagree

Q25 I continue to pay attention to information on vaccines in general.

1 Strongly agree

2 Somewhat agree

3 Unsure/no opinion

4 Somewhat disagree

5 Strongly disagree

Q26 The COVID-19 pandemic has made me more willing to get vaccinated against other diseases (e.g., flu, measles, viral hepatitis B).

1 Strongly agree

2 Somewhat agree

3 Unsure/no opinion

4 Somewhat disagree

5 Strongly disagree

Q27 My experience with the COVID-19 vaccine has affected my trust in other vaccines.

1 Increased trust

2 No effect

3 Decreased trust

4 Don't know

Q28 Broadcast media coverage on COVID-19 vaccines has affected my trust in other vaccines.

1 Increased trust

2 No effect

3 Decreased trust

4 Don’t know

Q29 Social media coverage on COVID-19 vaccines has affected my trust in other vaccines.

1 Increased trust

2 No effect

3 Decreased trust

4 Don’t know

Q30 If an mRNA vaccine is approved for a disease for which you are at risk, would you take it?

1 Definitely no

2 Unsure, but leaning towards no

3 Unsure, but leaning towards yes

4 Definitely yes

Q31 If the World Health Organization (WHO) announced a new pandemic threat, would you trust this information?

1 Yes

2 No

3 Don’t know

Q32 If the World Health Organization (WHO) announced a new pandemic threat and advised getting vaccinated, would you?

1 Yes

2 No

3 Don’t know

Q33 "On a scale of 1 (trust completely) to 10 (do not trust at all) how much do you trust the following sources of information about COVID-19 vaccines: My family and friends"

Q34 My employer

Q35 My doctor or nurse

Q36 My government

Q37 Religious leaders

Q38 News media (e.g., television, Internet, radio, newspapers)

Q39 Social media (e.g., Facebook, Twitter [X], Instagram, WhatsApp, LinkedIn, TikTok)

Q40 World Health Organization (WHO)

Q41 Centers for Disease Control and Prevention (CDC), USA

Q42 European Centre for Disease Prevention and Control (ECDC)

Q43 The public health authorities in my country

Q44 How confident are you that we will manage the next health crisis better than the COVID-19 pandemic?

1 Not at all confident

2 Somewhat confident

3 Very confident

4 Don’t know

Q45 From what you have read or heard, do you think climate change/global warming is a real threat or not a real threat?

1 Real threat

2 Not a real threat

Q46 What is your age?

Q47 What is your gender?

1 Male

2 Female

3 Prefer not to say

Q48 Education (country specific)

1 Less than High School

2 High School degree or GRE

3 Some College

4 Associate or Vocational degree

5 Bachelor Degree

6 Post Graduate Degree (Master, Lawyer, Doctor)

Q49 Median Income Level (country specific)

1 Yes, my monthly income is more than 7,453 Singapore dollars.

2 Yes, my monthly income is less than 7,453 Singapore dollars.

3 No, I do not have an income.

4 Refused/ Did not answer

Q50 Regions (country specific)

1 Core Central Region: Downtown Core (Marina Bay, Marina Centre, Raffles Place, Tanjong Pagar); Outram, Sentosa, Rochor, Orchard, Newton, River Valley, Bukit Timah, Holland Road, Tanglin, Novena, Thomson

2 Rest of Central Region: Bishan, Bukit Merah, Geylang, Kallang, Marine Parade, Queenstown, Southern Islands (except Sentosa), Toa Payoh

3 North Region: Central Water Catchment, Lim Chu Kang, Mandai, Sembawang, Simpang, Sungei Kadut, Woodlands, Yishun

4 North East Region: Ang Mo Kio, Hougang, North-Eastern Islands, Punggol, Seletar Sengkang,(Sengkang New Town, Rivervale, Compassvale, Buangkok, Anchorvale, Fernvale, Jalan Kayu), Serangoon

5 East Region: Bedok, Changi, Changi Bay, Paya Lebar, Pasir Ris, Tampines

6 West Region: Bukit Batok, Bukit Panjang, Boon Lay, Pioneer, Choa Chu Kang, Clementi, Jurong East, Jurong West, Tengah, Tuas, Western Islands, Western Water Catchment, Benoi, Ghim Moh, Gul, Pandan Gardens, Jurong Island, Kent Ridge, Nanyang, Pioneer, Pasir Laba, Teban Gardens, Toh Tuck, Tuas South

Spain

COVID-VAC: Una encuesta mundial sobre la percepción de la vacuna contra la COVID-19

El propósito de la encuesta es medir las reacciones de los residentes a los esfuerzos de respuesta de la COVID-19. Los riesgos de esta encuesta rutinaria se consideran mínimos. Es posible que se sienta incómodo al considerar las implicaciones de la COVID-19 y la participación en este estudio de investigación es completamente voluntaria y puede interrumpir su participación en cualquier momento. Sus respuestas serán anónimas y confidenciales. La encuesta debería durar menos de 5 minutos. Al responder a las preguntas, indica que ha leído la descripción del estudio, que es mayor de 18 años y que acepta los términos descritos.

Si tiene preguntas sobre sus derechos como participante en una investigación, puede ponerse en contacto con la Junta de Revisión Institucional (IRB) de Emerson College, que se ocupa de la protección de los voluntarios en los proyectos de investigación. Puede ponerse en contacto con el investigador principal a través del correo electrónico emersonpolling@emerson.edu o con el Presidente del IRB a través del correo electrónico human_subjects@emerson.edu.

Q1 "En primer lugar, por favor, indique en qué medida está de acuerdo con esta afirmación: El COVID-19 sigue siendo una peligrosa amenaza para la salud."

1 Sumamente de acuerdo

2 Algo de acuerdo

3 No estoy seguro(a)/no tengo una opinión

4 Algo en desacuerdo

5 Sumamente en desacuerdo

Q2 "Ahora, he aquí algunas preguntas relacionadas con las vacunas contra el COVID-19. El COVID-19 se puede prevenir mediante la vacunación."

1 Sumamente de acuerdo

2 Algo de acuerdo

3 No estoy seguro(a)/no tengo una opinión

4 Algo en desacuerdo

5 Sumamente en desacuerdo

Q3 Los riesgos de la enfermedad del COVID-19 son mayores que los riesgos de la vacuna.

1 Sumamente de acuerdo

2 Algo de acuerdo

3 No estoy seguro(a)/no tengo una opinión

4 Algo en desacuerdo

5 Sumamente en desacuerdo

Q4 Las vacunas contra el COVID-19 que hay disponibles para mí son seguras.

1 Sumamente de acuerdo

2 Algo de acuerdo

3 No estoy seguro(a)/no tengo una opinión

4 Algo en desacuerdo

5 Sumamente en desacuerdo

Q5 Confío en la ciencia que hay detrás de las vacunas contra el COVID-19 que hay disponibles para mí.

1 Sumamente de acuerdo

2 Algo de acuerdo

3 No estoy seguro(a)/no tengo una opinión

4 Algo en desacuerdo

5 Sumamente en desacuerdo

Q6 Las vacunas contra el COVID-19 que hay disponibles para mí son efectivas protegiéndome contra el COVID-19 grave.

1 Sumamente de acuerdo

2 Algo de acuerdo

3 No estoy seguro(a)/no tengo una opinión

4 Algo en desacuerdo

5 Sumamente en desacuerdo

Q7 ¿Ha recibido usted al menos una dosis de vacuna contra el COVID-19?

1 Sí, recibí una dosis

2 Sí, recibí dos o más dosis

3 No

Q8 ¿Cuál o cuáles de las siguientes vacunas contra el COVID-19 recibió? Seleccione todas las opciones pertinentes:

1 Moderna (Spikevax)

2 Pfizer (Comirnaty)

3 Johnson&Johnson (Janssen o J&J)

4 Sinovac

5 Astra-Zeneca (Oxford, Vaxzevria)

6 COVAXIN

7 Otra opción (por favor, especifique)

8 No lo sé

Q9 Me administraré la dosis de refuerzo contra el COVID-19 recomendada.

1 Sumamente de acuerdo

2 Algo de acuerdo

3 No estoy seguro(a)/no tengo una opinión

4 Algo en desacuerdo

5 Sumamente en desacuerdo

Q10 Creo que la pandemia de COVID-19 ha terminado.

1 Sumamente de acuerdo

2 Algo de acuerdo

3 No estoy seguro(a)/no tengo una opinión

4 Algo en desacuerdo

5 Sumamente en desacuerdo

Q11 ¿En qué medida confió en la gestión que hizo su gobierno de la pandemia de COVID-19 en su país?

1 No confié

2 No confié demasiado

3 No estoy seguro(a)

4 Confié algo

5 Confié

6 No lo sé o no lo recuerdo

Q12 ¿Afectó el desarrollo de las vacunas contra el COVID-19 a su confianza en la industria farmacéutica?

1 Aumentó mi confianza

2 No tuvo efecto

3 Disminuyó mi confianza

4 No lo sé o no lo recuerdo

Q13 ¿Afectó el desarrollo de vacunas contra el COVID-19 a su confianza en la ciencia en general?

1 Aumentó mi confianza

2 No tuvo efecto

3 Disminuyó mi confianza

4 No lo sé o no lo recuerdo

Q14 ¿En qué medida confía en las autoridades sanitarias que recomendaron que se administrara una vacuna contra el COVID-19?

1 Nada en absoluto

2 Un poco

3 Moderadamente

4 Mucho

5 No lo sé o no lo recuerdo

Q15 Durante la pandemia, ¿tuvo que retrasar o cancelar algún servicio médico que necesitaba, no relacionado con el COVID-19?

1 Sí

2 No

3 No lo sé o no lo recuerdo

Q16 ¿Acataría usted una exigencia (mandato) de vacunación en el futuro de alguna de las siguientes partes? Seleccione todas las opciones pertinentes:

1 Gobierno

2 Empleador

3 Escuela o universidad

4 Ninguna de las opciones anteriores

5 No lo sé

Q17 ¿Acataría usted una exigencia (mandato) de mascarilla en el futuro de alguna de las siguientes partes? Seleccione todas las opciones pertinentes:

1 Gobierno

2 Empleador

3 Escuela o universidad

4 Ninguna de las opciones anteriores

5 No lo sé

Q18 Por lo que sabe, ¿tiene o ha tenido usted COVID-19?

1 Sí

2 No

3 No lo sé o no lo recuerdo

Q19 "El COVID persistente ha sido definido como "síntomas que pueden durar semanas o meses con posterioridad a la recuperación de una enfermedad grave". Por lo que sabe, ¿tiene o ha tenido usted COVID-19 persistente? "

1 Sí

2 No

3 No lo sé o no lo recuerdo

Q20 ¿Tomó alguno de los siguientes medicamentos para tratar el COVID-19? Seleccione todas las opciones pertinentes:

1 Paxlovid

2 Molnupiravir (Lagevrio)

3 Anticuerpos monoclonales (Olumiant/Baricitinib)

4 Ivermectin

5 Medicina tradicional, extractos y tratamientos herbarios

6 No lo sé o no lo recuerdo

7 No, no tomé medicamentos contra el COVID-19

Q21 ¿Ha perdido usted a un miembro de su familia o un amigo cercano debido a la enfermedad del COVID-19? Seleccione todas las opciones pertinentes:

1 Sí, durante el año pasado

2 Sí, hace más de un año

3 No

Q22 Estuve satisfecho(a) con los esfuerzos de comunicación al respecto de vacunas contra el COVID-19 que hicieron las autoridades sanitarias en mi país.

1 Sumamente de acuerdo

2 Algo de acuerdo

3 No estoy seguro(a)/no tengo una opinión/no lo recuerdo

4 Algo en desacuerdo

5 Sumamente en desacuerdo

Q23 Para mí es fácil saber la diferencia entre información exacta y falsa que hay en internet acerca de la vacuna contra el COVID-19.

1 Sumamente de acuerdo

2 Algo de acuerdo

3 No estoy seguro(a)/no tengo una opinión/no lo recuerdo

4 Algo en desacuerdo

5 Sumamente en desacuerdo

Q24 Sigo prestando atención a la información sobre vacunas contra el COVID-19.

1 Sumamente de acuerdo

2 Algo de acuerdo

3 No estoy seguro(a)/no tengo una opinión/no lo recuerdo

4 Algo en desacuerdo

5 Sumamente en desacuerdo

Q25 Sigo prestando atención a la información sobre vacunas en general.

1 Sumamente de acuerdo

2 Algo de acuerdo

3 No estoy seguro(a)/no tengo una opinión

4 Algo en desacuerdo

5 Sumamente en desacuerdo

Q26 La pandemia de COVID-19 ha hecho que esté más dispuesto(a) a vacunarme contra otras enfermedades (p.ej. gripe, sarampión, hepatitis B vírica).

1 Sumamente de acuerdo

2 Algo de acuerdo

3 No estoy seguro(a)/no tengo una opinión

4 Algo en desacuerdo

5 Sumamente en desacuerdo

Q27 Mi experiencia con la vacuna contra el COVID-19 ha afectado a mi confianza en otras vacunas.

1 Aumentó mi confianza

2 No tuvo efecto

3 Disminuyó mi confianza

4 No lo sé

Q28 La cobertura de los medios al respecto de vacunas contra el COVID-19 ha afectado a mi confianza en otras vacunas.

1 Aumentó mi confianza

2 No tuvo efecto

3 Disminuyó mi confianza

4 No lo sé

Q29 La cobertura de las redes sociales al respecto de vacunas contra el COVID-19 ha afectado a mi confianza en otras vacunas.

1 Aumentó mi confianza

2 No tuvo efecto

3 Disminuyó mi confianza

4 No lo sé

Q30 Si se aprobara una vacuna ARNm para una enfermedad de la que usted estuviera en riesgo, ¿se la administraría?

1 Definitivamente no

2 No estoy seguro(a), pero me inclino por el no

3 No estoy seguro(a), pero me inclino por el sí

4 Definitivamente sí

Q31 Si la Organización Mundial de la Salud (OMS) anunciara la amenaza de una nueva pandemia, ¿confiaría usted en esta información?

1 Sí

2 No

3 No lo sé

Q32 Si la Organización Mundial de la Salud (OMS) anunciara la amenaza de una nueva pandemia y aconsejara vacunarse, ¿usted lo haría?

1 Sí

2 No

3 No lo sé

Q33 "En una escala de 1 (confío completamente) y 10 (no confío nada en absoluto), por favor, indique en qué medida confía en las siguientes fuentes de información sobre vacunas contra el COVID-19: Mi familia y amigos"

Q34 Mi empleador

Q35 Mi médico o enfermero

Q36 Mi gobierno

Q37 Líderes religiosos

Q38 Medios de noticias (p.ej., televisión, internet, radio, periódicos)

Q39 Redes sociales (p.ej., Facebook, Twitter [X], Instagram, WhatsApp, LinkedIn, TikTok)

Q40 Organización Mundial de la Salud (OMS)

Q41 Centros para el Control y la Prevención de Enfermedades (CMC, por sus siglas en inglés), EE.UU.

Q42 Centro Europeo para la Prevención y el Control de Enfermedades (ECDC, por sus siglas en inglés)

Q43 Las autoridades sanitarias públicas en mi país

Q44 ¿En qué medida está usted seguro(a) de que la próxima crisis sanitaria la gestionaremos mejor que la pandemia de COVID-19?

1 Nada seguro(a) en absoluto

2 Algo seguro(a)

3 Muy seguro(a)

4 No lo sé

Q45 En base a lo que ha leído u oído, ¿cree que el cambio climático/calentamiento global es una amenaza real, o no es una amenaza real?

1 Amenaza real

2 No una amenaza real

Q46 ¿Cuál es su edad?

Q47 ¿Cuál es su género?

1 Masculino

2 Femenino

3 Prefiero no decirlo

Q48 Educación (específica por país)

1 Educación primaria

2 Educación secundaria

3 Certificado de bachillerato

4 Formación profesional

5 Título de licenciatura universitaria

6 Postgrado universitario

Q49 Nivel de ingresos medio (específico por país)

1 Sí, mis ingresos mensuales son superiores a 2.380 euros.

2 Sí, mis ingresos mensuales son inferiores a 2.380 euros.

3 No, no tengo ingresos.

4 Rehúsa/ No respondió

Q50 Regiones (específicas por país)

1 Andalucía

2 Sevilla

3 Cataluña

4 Comunidad de Madrid

5 Comunidad Valenciana

6 Galicia Castilla y León

7 Comunidad Autónoma Vasca

8 Castilla-La Mancha

9 Islas Canarias

10 Región de Murcia

11 Aragón

12 Extremadura

13 Islas Baleares

14 Principado de Asturias

15 Comunidad Foral de Navarra

16 Cantabria

17 La Rioja

Sweden

COVID-VAC: En global undersökning av uppfattningar om covid-19-vaccin

Syftet med undersökningen är att mäta invånares reaktioner på covid-19-insatserna. Riskerna med denna rutinundersökning anses vara minimala. Du kan känna dig obekväm när du tänker på följderna av covid-19. Deltagandet i denna studie är helt frivilligt och du kan när som helst avbryta din medverkan. Dina svar kommer att förbli anonyma och konfidentiella. Undersökningen tar mindre än fem minuter att genomföra. Genom att svara på frågorna bekräftar du att du har läst beskrivningen av studien, att du är över 18 år och att du godkänner de villkor som beskrivs.

Om du har frågor om dina rättigheter som deltagare i forskningen kan du kontakta Emerson College Institutional Review Board (IRB), som har till uppgift att skydda frivilliga i forskningsprojekt. Du kan nå den ledande utredaren via e-post på emersonpolling@emerson.edu eller ordföranden för IRB genom att skicka e-post till human_subjects@emerson.edu.

Q1 "Börja med att ange i vilken utsträckning du håller med om detta påstående: Covid-19 fortsätter att vara ett farligt hot mot hälsan."

1 Håller helt med

2 Håller delvis med

3 Osäker/Ingen uppfattning

4 Håller inte helt med

5 Håller inte alls med

Q2 "Här följer nu några frågor som rör covid-19-vacciner. Covid-19 kan förhindras genom vaccination."

1 Håller helt med

2 Håller delvis med

3 Osäker/Ingen uppfattning

4 Håller inte helt med

5 Håller inte alls med

Q3 Riskerna med covid-19-sjukdomen är större än riskerna med vaccinet.

1 Håller helt med

2 Håller delvis med

3 Osäker/Ingen uppfattning

4 Håller inte helt med

5 Håller inte alls med

Q4 Covid-19-vaccinerna som finns tillgängliga för mig är säkra.

1 Håller helt med

2 Håller delvis med

3 Osäker/Ingen uppfattning

4 Håller inte helt med

5 Håller inte alls med

Q5 Jag litar på vetenskapen bakom covid-19-vaccinerna som finns tillgängliga för mig.

1 Håller helt med

2 Håller delvis med

3 Osäker/Ingen uppfattning

4 Håller inte helt med

5 Håller inte alls med

Q6 Covid-19-vaccinerna som finns tillgängliga för mig skyddar effektivt mot allvarlig covid-19.

1 Håller helt med

2 Håller delvis med

3 Osäker/Ingen uppfattning

4 Håller inte helt med

5 Håller inte alls med

Q7 Har du fått minst en dos av ett covid-19-vaccin?

1 Ja, jag har fått en dos

2 Ja, jag har fått två eller fler doser

3 Nej

Q8 Vilket av följande covid-19-vacciner fick du? Du kan välja flera alternativ:

1 Moderna (Spikevax)

2 Pfizer (Comirnaty)

3 Johnson&Johnson (Janssen eller J&J)

4 Sinovac

5 Astra-Zeneca (Oxford, Vaxzevria)

6 COVAXIN

7 Annat (specificera)

8 Vet ej

Q9 Jag ska ta den rekommenderade påfyllnadsdosen av covid-19-vaccinet.

1 Håller helt med

2 Håller delvis med

3 Osäker/Ingen uppfattning

4 Håller inte helt med

5 Håller inte alls med

Q10 Jag tror att covid-19-pandemin är över.

1 Håller helt med

2 Håller delvis med

3 Osäker/Ingen uppfattning

4 Håller inte helt med

5 Håller inte alls med

Q11 I vilken utsträckning litade du på myndigheternas hantering av covid-19-pandemin i ditt land?

1 Hade inget förtroende

2 Hade inte riktigt förtroende

3 Osäker

4 Hade lite förtroende

5 Hade förtroende

6 Vet inte eller kommer inte ihåg

Q12 Påverkade utvecklingen av covid-19-vaccinerna din tilltro till läkemedelsindustrin?

1 Ökat tilltron

2 Ingen påverkan

3 Minskat tilltron

4 Vet inte eller kommer inte ihåg

Q13 Påverkade utvecklingen av covid-19-vaccinerna din tilltro till vetenskap i allmänhet?

1 Ökat tilltron

2 Ingen påverkan

3 Minskat tilltron

4 Vet inte eller kommer inte ihåg

Q14 I vilken utsträckning litar du på hälso- och sjukvårdsmyndigheterna som rekommenderade att du skulle vaccinera dig mot covid-19?

1 Inte alls

2 Lite

3 Måttligt

4 Väldigt mycket

5 Vet inte eller kommer inte ihåg

Q15 Behövde du under pandemin skjuta upp eller avbryta någon medicinsk åtgärd utan samband med covid-19?

1 Ja

2 Nej

3 Vet inte eller kommer inte ihåg

Q16 Skulle du följa ett krav (obligatoriskt) på vaccination i framtiden från någon av följande? Du kan välja flera alternativ:

1 Regering

2 Arbetsgivare

3 Skola eller universitet

4 Ingen av ovanstående

5 Vet ej

Q17 Skulle du följa ett krav (obligatoriskt) på munskydd i framtiden från någon av följande? Du kan välja flera alternativ:

1 Regering

2 Arbetsgivare

3 Skola eller universitet

4 Ingen av ovanstående

5 Vet ej

Q18 Har du eller har du, såvitt du vet, haft covid-19?

1 Ja

2 Nej

3 Vet inte eller kommer inte ihåg

Q19 "Postcovid (långtidscovid) har definierats som ""symtom som kan vara i veckor eller månader efter att man tillfrisknat från akut sjukdom”. Har du eller har du, såvitt du vet, haft postcovid (långtidscovid)? "

1 Ja

2 Nej

3 Vet inte eller kommer inte ihåg

Q20 Har du tagit något av följande läkemedel för att behandla covid-19? Du kan välja flera alternativ:

1 Paxlovid

2 Molnupiravir (Lagevrio)

3 Monoklonala antikroppar (Olumiant/Baricitinib)

4 Ivermectin

5 Klassisk läkekonst, örtextrakt och behandlingar

6 Vet inte eller kommer inte ihåg

7 Nej, jag har inte tagit läkemedel mot covid-19

Q21 Har du förlorat familjemedlemmar eller nära vänner i covid-19-sjudomen? Du kan välja flera alternativ:

1 Ja, under det senaste året

2 Ja, för mer än ett år sedan

3 Nej

Q22 Jag var nöjd med hälso- och sjukvårdsmyndigheternas kommunikationsarbete beträffande covid-19-vaccinerna i mitt land.

1 Håller helt med

2 Håller delvis med

3 Osäker/ingen uppfattning/kommer inte ihåg

4 Håller inte helt med

5 Håller inte alls med

Q23 Jag har lätt för att skilja mellan sann och falsk information om covid-19-vaccin på internet.

1 Håller helt med

2 Håller delvis med

3 Osäker/ingen uppfattning/kommer inte ihåg

4 Håller inte helt med

5 Håller inte alls med

Q24 Jag fortsätter att vara uppmärksam på information om covid-19-vacciner.

1 Håller helt med

2 Håller delvis med

3 Osäker/ingen uppfattning/kommer inte ihåg

4 Håller inte helt med

5 Håller inte alls med

Q25 Jag fortsätter att vara uppmärksam på information om vacciner i allmänhet.

1 Håller helt med

2 Håller delvis med

3 Osäker/Ingen uppfattning

4 Håller inte helt med

5 Håller inte alls med

Q26 Covid-19-pandemin har gjort mig mer villig att vaccinera mig mot andra sjukdomar [t.ex. influensa, mässling, HBV (hepatit B-virus)].

1 Håller helt med

2 Håller delvis med

3 Osäker/Ingen uppfattning

4 Håller inte helt med

5 Håller inte alls med

Q27 Min erfarenhet av covid-19-vaccinet har påverkat min tillit till andra vacciner.

1 Ökat tilltron

2 Ingen påverkan

3 Minskat tilltron

4 Vet ej

Q28 Etermedias täckning av covid-19-vaccinerna har påverkat min tilltro till andra vacciner.

1 Ökat tilltron

2 Ingen påverkan

3 Minskat tilltron

4 Vet ej

Q29 Sociala mediers täckning av covid-19-vaccinerna har påverkat min tillit till andra vacciner.

1 Ökat tilltron

2 Ingen påverkan

3 Minskat tilltron

4 Vet ej

Q30 Om ett mRNA-vaccin som godkänns för en sjukdom som du löper risk att få, skulle du ta det?

1 Absolut inte

2 Osäker, men lutar åt nej

3 Osäker, men lutar åt ja

4 Ja, absolut

Q31 Om WHO (Världshälsoorganisationen) tillkännagav ett nytt pandemihot, skulle du lita på informationen?

1 Ja

2 Nej

3 Vet ej

Q32 Om WHO (Världshälsoorganisationen) tillkännagav ett nytt pandemihot och uppmanade till vaccination, skulle du göra det?

1 Ja

2 Nej

3 Vet ej

Q33 "På en skala mellan 1 (har fullt förtroende) och 10 (har inget förtroende alls), hur mycket skulle du lita på följande informationskällor om Covid-19-vacciner: Min familj och mina vänner"

Q34 Min arbetsgivare

Q35 Min läkare eller sjuksköterska

Q36 Min regering

Q37 Religiösa ledare

Q38 Nyhetsmedia (t.ex. TV, internet, radio, dagstidinga)

Q39 Sociala medier (t.ex. Facebook, Twitter [X], Instagram, WhatsApp, LinkedIn, TikTok)

Q40 WHO (Världshälsoorganiationen)

Q41 Centers for Disease Control and Prevention (CDC), USA

Q42 Europeiskt centrum för förebyggande och kontroll av sjukdomar (ECDC)

Q43 Hälso- och sjukvårdsmyndigheterna i mitt land

Q44 Hur säker är du på att vi kommer att klara nästa hälsokris bättre än covid-19-pandemin?

1 Inte alls säker

2 Lite säker

3 Mycket säker

4 Vet ej

Q45 Med utgångspunkt från vad du har läst eller hört, tror du att klimatförändringarna/den globala uppvärmningen är ett verkligt hot eller inte?

1 Verkligt hot

2 Inget verkligt hot

Q46 Hur gammal är du?

Q47 Är du ...?

1 Man

2 Kvinna

3 Föredrar att inte svara

Q48 Utbildning (landsspecifik)

1 Gymnasieutbildning eller lägre

2 Viss universitetsutbildning

3 Kandidatexamen

4 Masterexamen eller högre

Q49 Medianinkomstnivå (landsspecifik)

1 Ja, min månadsinkomst är över 54 613 kronor.

2 Ja, min månadsinkomst är lägre än 54 613 kronor.

3 Nej, jag saknar inkomst.

4 Vägrade svara/Svarade inte

Q50 Regioner (landsspecifik)

1 Norrland

2 Svealand

3 Götaland

Türkiye

COVID-VAC: COVID-19 aşı algısı kapsamında küresel anket

Bu anketin amacı kişinin COVID-19 müdahale çabalarına yönelik tepkilerini ölçmektir. Bu rutin anketin risklerinin asgari düzeyde olduğu düşünülmektedir. COVID-19'un etkileri göz önünde bulundurulduğunda huzursuz hissedebilirsiniz; bu araştırmaya katılım tamamen gönüllülüğe dayalıdır, dilediğiniz zaman katılımınıza son verebilirsiniz. Cevaplarınız tamamen isimsiz ve gizli kalacaktır. Anketin tamamlanması 5 dakikadan kısa sürer. Bu soruları cevaplayarak çalışmanın tanımını okuduğunuzu, 18 yaşından büyük olduğunuzu ve tanımlandığı şekilde koşulları kabul ettiğinizi beyan etmiş olursunuz.

Bir araştırma katılımcısı olarak haklarınız konusunda sorularınız olursa araştırma projelerinde gönüllü katılımcıların korunması konusuyla ilgilenen Emerson College Institutional Review Board (IRB) ile iletişime geçebilirsiniz. Araştırma sorumlusuna emersonpolling@emerson.edu veya IRB Başkanı'na human_subjects@emerson.edu adresleri üzerinden ulaşabilirsiniz.

1 "Öncelikle, lütfen bu ifadeye ne ölçüde katıldığınızı belirtin: COVID-19 tehlikeli bir sağlık tehdidi olmaya devam etmektedir."

1 Kesinlikle katılıyorum

2 Kısmen katılıyorum

3 Emin değilim/fikrim yok

4 Pek katılmıyorum

5 Kesinlikle katılmıyorum

Q2 "Şimdi COVID-19 aşıları ile ilgili bazı sorular soracağız. COVID-19 aşılama ile önlenebilir."

1 Kesinlikle katılıyorum

2 Kısmen katılıyorum

3 Emin değilim/fikrim yok

4 Pek katılmıyorum

5 Kesinlikle katılmıyorum

Q3 COVID-19 hastalığının riskleri aşının risklerinden daha fazladır.

1 Kesinlikle katılıyorum

2 Kısmen katılıyorum

3 Emin değilim/fikrim yok

4 Pek katılmıyorum

5 Kesinlikle katılmıyorum

Q4 Bana sunulan COVID-19 aşıları güvenlidir.

1 Kesinlikle katılıyorum

2 Kısmen katılıyorum

3 Emin değilim/fikrim yok

4 Pek katılmıyorum

5 Kesinlikle katılmıyorum

Q5 Bana sunulan COVID-19 aşılarının arkasındaki bilime güveniyorum.

1 Kesinlikle katılıyorum

2 Kısmen katılıyorum

3 Emin değilim/fikrim yok

4 Pek katılmıyorum

5 Kesinlikle katılmıyorum

Q6 Bana sunulan COVID-19 aşıları ağır seviyedeki COVID-19'a karşı korumada etkilidir.

1 Kesinlikle katılıyorum

2 Kısmen katılıyorum

3 Emin değilim/fikrim yok

4 Pek katılmıyorum

5 Kesinlikle katılmıyorum

Q7 En az bir doz COVID-19 aşısı oldunuz mu?

1 Evet, bir doz aşı oldum

2 Evet, iki veya daha fazla doz aşı oldum

3 Hayır

Q8 Aşağıdaki COVID-19 aşılarından hangilerini oldunuz? Uygun olanların tümünü seçin:

1 Moderna (Spikevax)

2 Pfizer (Comirnaty)

3 Johnson&Johnson (Janssen veya J&J)

4 Sinovac

5 Astra-Zeneca (Oxford, Vaxzevria)

6 COVAXIN

7 Diğer (lütfen belirtin)

8 Bilmiyorum

Q9 Tavsiye edilen COVID-19 aşısı takviye dozunu olacağım.

1 Kesinlikle katılıyorum

2 Kısmen katılıyorum

3 Emin değilim/fikrim yok

4 Pek katılmıyorum

5 Kesinlikle katılmıyorum

Q10 COVID-19 pandemisinin bittiğine inanıyorum.

1 Kesinlikle katılıyorum

2 Kısmen katılıyorum

3 Emin değilim/fikrim yok

4 Pek katılmıyorum

5 Kesinlikle katılmıyorum

Q11 Ülkenizde COVID-19 pandemisinin yönetilmesi konusunda hükümetinize ne kadar güveniyordunuz?

1 Hiç güvenmedim

2 Pek güvenmedim

3 Emin değilim

4 Kısmen güvendim

5 Güvendim

6 Bilmiyorum veya hatırlamıyorum

Q12 COVID-19 aşılarının geliştirilmesi ilaç sektörüne olan güveninizi etkiledi mi?

1 Güvenim arttı

2 Etkilemedi

3 Güvenim azaldı

4 Bilmiyorum veya hatırlamıyorum

Q13 COVID-19 aşılarının geliştirilmesi genel anlamda bilime olan güveninizi etkiledi mi?

1 Güvenim arttı

2 Etkilemedi

3 Güvenim azaldı

4 Bilmiyorum veya hatırlamıyorum

Q14 COVID-19 aşısı olmanızı tavsiye eden sağlık otoritelerine ne kadar güveniyorsunuz?

1 Hiç

2 Az

3 Kısmen

4 Çok

5 Bilmiyorum veya hatırlamıyorum

Q15 Pandemi sırasında COVID-19 haricinde ihtiyacınız olan başka tıbbi hizmetleri ertelemek veya iptal etmek zorunda kaldınız mı?

1 Evet

2 Hayır

3 Bilmiyorum veya hatırlamıyorum

Q16 Gelecekte aşağıdakilerden herhangi birinin ilan ettiği bir aşı zorunluluğuna (talimatına) uyacak mısınız? Uygun olanların tümünü seçin:

1 Hükümet

2 İşveren

3 Okul veya üniversite

4 Yukarıdakilerin hiçbiri

5 Bilmiyorum

Q17 Gelecekte aşağıdakilerden herhangi birinin ilan ettiği bir maske zorunluluğuna (talimatına) uyacak mısınız? Uygun olanların tümünü seçin:

1 Hükümet

2 İşveren

3 Okul veya üniversite

4 Yukarıdakilerin hiçbiri

5 Bilmiyorum

Q18 Bildiğiniz kadarıyla COVID geçirdiniz mi veya hala devam ediyor mu?

1 Evet

2 Hayır

3 Bilmiyorum veya hatırlamıyorum

Q19 "Uzun süreli COVID, ""akut hastalıktan iyileştikten sonra haftalar veya aylar boyunca sürebilen semptomlar"" olarak tanımlanmıştır. Bildiğiniz kadarıyla Uzun COVID geçirdiniz mi veya hala devam ediyor mu? "

1 Evet

2 Hayır

3 Bilmiyorum veya hatırlamıyorum

Q20 COVID-19 tedavisi için aşağıdaki ilaçlardan herhangi birini kullandınız mı? Uygun olanların tümünü seçin:

1 Paxlovid

2 Molnupiravir (Lagevrio)

3 Monoklonal antikorlar (Olumiant/Baricitinib)

4 Ivermectin

5 Geleneksel ilaç, bitki özleri ve tedavileri

6 Bilmiyorum veya hatırlamıyorum

7 Hayır, COVID-19 için ilaç kullanmadım

Q21 COVID-19 hastalığı nedeniyle bir aile üyesini veya yakın arkadaşınızı kaybettiniz mi? Uygun olanların tümünü seçin:

1 Evet, son bir yıl içinde

2 Evet, bir yıldan daha önce

3 Hayır

Q22 Ülkemde COVID-19 aşıları ile ilgili olarak sağlık otoriteleri tarafından yapılan iletişim çalışmalarından memnum kaldım.

1 Kesinlikle katılıyorum

2 Kısmen katılıyorum

3 Emin değilim/fikrim yok/hatırlamıyorum

4 Pek katılmıyorum

5 Kesinlikle katılmıyorum

Q23 COVID-19 aşısı ile ilgili olarak internette dolaşan doğru ve yanlış bilgiler arasındaki farkı kolayca ayırt edebiliyorum.

1 Kesinlikle katılıyorum

2 Kısmen katılıyorum

3 Emin değilim/fikrim yok/hatırlamıyorum

4 Pek katılmıyorum

5 Kesinlikle katılmıyorum

Q24 COVID-19 aşıları ile ilgili bilgiler hala ilgimi çekiyor.

1 Kesinlikle katılıyorum

2 Kısmen katılıyorum

3 Emin değilim/fikrim yok/hatırlamıyorum

4 Pek katılmıyorum

5 Kesinlikle katılmıyorum

Q25 Genel anlamda aşılarla ilgili bilgiler hala ilgimi çekiyor.

1 Kesinlikle katılıyorum

2 Kısmen katılıyorum

3 Emin değilim/fikrim yok

4 Pek katılmıyorum

5 Kesinlikle katılmıyorum

Q26 COVID-19 pandemisinden sonra diğer hastalıklara (ör. grip, kızamık, viral hepatit B) karşı aşı olmaya daha istekli hale geldim.

1 Kesinlikle katılıyorum

2 Kısmen katılıyorum

3 Emin değilim/fikrim yok

4 Pek katılmıyorum

5 Kesinlikle katılmıyorum

Q27 COVID-19 aşısı ile yaşadığım deneyim diğer aşılara olan güvenimi etkiledi.

1 Güvenim arttı

2 Etkilemedi

3 Güvenim azaldı

4 Bilmiyorum

Q28 COVID-19 aşıları ile ilgili olarak görsel medyada çıkan haberler diğer aşılara olan güvenimi etkiledi.

1 Güvenim arttı

2 Etkilemedi

3 Güvenim azaldı

4 Bilmiyorum

Q29 COVID-19 aşıları ile ilgili olarak sosyal medyada çıkan haberler diğer aşılara olan güvenimi etkiledi.

1 Güvenim arttı

2 Etkilemedi

3 Güvenim azaldı

4 Bilmiyorum

Q30 Risk grubunda olduğunuz bir hastalık için bir mRNA aşısına onay verilse, bu aşıyı olur musunuz?

1 Kesinlikle hayır

2 Emin değilim, fakat büyük olasılıkla hayır

3 Emin değilim, fakat büyük olasılıkla evet

4 Kesinlikle evet

Q31 Dünya Sağlık Örgütü (DSÖ) yeni bir pandemi tehdidi olduğunu açıklarsa, bu bilgiye güvenir misiniz?

1 Evet

2 Hayır

3 Bilmiyorum

Q32 Dünya Sağlık Örgütü (DSÖ) yeni bir pandemi tehdidi olduğunu açıklarsa ve aşı olunmasını tavsiye ederse, aşı olur musunuz?

1 Evet

2 Hayır

3 Bilmiyorum

Q33 "1'den (tamamen güveniyorum) 10'a (hiç güvenmiyorum) kadar bir ölçeğe göre, COVID-19 aşıları ile ilgili aşağıdaki bilgi kaynaklarına ne kadar güveniyorsunuz: Ailem ve arkadaşlarım"

Q34 İşverenim

Q35 Doktorum veya hemşirem

Q36 Hükümetim

Q37 Dini liderler

Q38 Haber medyası (ör. televizyon, internet, radyo, gazeteler)

Q39 Sosyal medya (ör. Facebook, Twitter [X], Instagram, WhatsApp, LinkedIn, TikTok)

Q40 Dünya Sağlık Örgütü (DSÖ)

Q41 Hastalık Kontrol ve Önleme Merkezleri (CDC), ABD

Q42 Avrupa Hastalık Önleme ve Kontrol Merkezi (ECDC)

Q43 Ülkemdeki halk sağlığı otoriteleri

Q44 Bir sonraki sağlık krizini COVID-19 pandemisinden daha iyi yöneteceğimize ne kadar güveniyorsunuz?

1 Hiç güvenmiyorum

2 Kısmen güveniyorum

3 Çok güveniyorum

4 Bilmiyorum

Q45 Okuduklarınıza veya duyduklarınıza dayanarak iklim değişikliğinin/küresel ısınmanın gerçek bir tehdit olduğunu düşünüyor musunuz?

1 Gerçek bir tehdit

2 Gerçek bir tehdit değil

Q46 Kaç yaşındasınız?

Q47 Cinsiyetiniz nedir?

1 Erkek

2 Kadın

3 Belirtmek istemiyorum

Q48 Eğitim (ülkeye özgü)

1 Liseden az

2 Lise derecesi

3 Üniversite terk

4 Ön Lisans veya Meslek derecesi

5 Üniversite Derecesi

6 Yüksek Lisans Derecesi (Master, Avukat, Doktor)

Q49 Ortalama Gelir Seviyesi (ülkeye özgü)

1 Evet, aylık gelirim 23.788 TL'den fazla.

2 Evet, aylık gelirim 23.788 TL'den az.

3 Hayır, gelirim yok.

4 Ret/ Cevap Yok

Q50 Bölgeler (ülkeye özgü)

1 Marmara Bölgesi

2 Ege Bölgesi

3 Karadeniz Bölgesi

4 Akdeniz Bölgesi

5 İç Anadolu Bölgesi

6 Güneydoğu Anadolu Bölgesi

7 Doğu Anadolu Bölgesi

United Kingdom

COVID-VAC: A global survey of COVID-19 vaccine perceptions

The purpose of the survey is to measure resident's reactions to COVID-19 response efforts. The risks in this routine survey are considered minimal. You may feel uncomfortable considering the implications of COVID-19 and participation in this research study is completely voluntary, and you may discontinue participation at any time. Your responses will remain anonymous and confidential. The survey should take less than 5 minutes to complete. By answering the questions, you are indicating that you have read the description of the study, that you are over the age of 18 and that you agree to the terms as described.

If you have questions about your rights as a research participant, you may contact the Emerson College Institutional Review Board (IRB), which is concerned with the protection of volunteers in research projects. You may reach the lead investigator via email at emersonpolling@emerson.edu or the Chair of the IRB by e-mailing human_subjects@emerson.edu.

Q1 "First, please indicate your level of agreement with this statement: COVID-19 remains a dangerous health threat."

1 Strongly agree

2 Somewhat agree

3 Unsure/no opinion

4 Somewhat disagree

5 Strongly disagree

Q2 "Now, here are some questions related to COVID-19 vaccines. COVID-19 can be prevented by vaccination."

1 Strongly agree

2 Somewhat agree

3 Unsure/no opinion

4 Somewhat disagree

5 Strongly disagree

Q3 The risks of COVID-19 disease are greater than the risks of the vaccine.

1 Strongly agree

2 Somewhat agree

3 Unsure/no opinion

4 Somewhat disagree

5 Strongly disagree

Q4 The COVID-19 vaccines available to me are safe.

1 Strongly agree

2 Somewhat agree

3 Unsure/no opinion

4 Somewhat disagree

5 Strongly disagree

Q5 I trust the science behind the COVID-19 vaccines available to me.

1 Strongly agree

2 Somewhat agree

3 Unsure/no opinion

4 Somewhat disagree

5 Strongly disagree

Q6 The COVID-19 vaccines available to me are effective in protecting against severe COVID-19.

1 Strongly agree

2 Somewhat agree

3 Unsure/no opinion

4 Somewhat disagree

5 Strongly disagree

Q7 Have you received at least one dose of a COVID-19 vaccine?

1 Yes, I received one dose

2 Yes, I received two or more doses

3 No

Q8 Which of the following COVID-19 vaccines did you receive? Select all that apply:

1 Moderna (Spikevax)

2 Pfizer (Comirnaty)

3 Johnson&Johnson (Janssen or J&J)

4 Sinovac

5 Astra-Zeneca (Oxford, Vaxzevria)

6 COVAXIN

7 Other (please specify)

8 Don't know

Q9 I will take the recommended COVID-19 booster.

1 Strongly agree

2 Somewhat agree

3 Unsure/no opinion

4 Somewhat disagree

5 Strongly disagree

Q10 I believe the COVID-19 pandemic is over.

1 Strongly agree

2 Somewhat agree

3 Unsure/no opinion

4 Somewhat disagree

5 Strongly disagree

Q11 How much did you trust your government's management of the COVID-19 pandemic in your country?

1 Did not trust

2 Somewhat did not trust

3 Unsure

4 Somewhat trust

5 Trust

6 Don't know or don't remember

Q12 Did the development of the COVID-19 vaccines affect your trust in the pharmaceutical industry?

1 Increased trust

2 No effect

3 Decreased trust

4 Don't know or don't remember

Q13 Did the development of COVID-19 vaccines affect your trust in science generally?

1 Increased trust

2 No effect

3 Decreased trust

4 Don't know or don't remember

Q14 How much do you trust the health authorities that recommended you get a COVID-19 vaccine?

1 Not at all

2 A little

3 Moderately

4 Very much

5 Don’t know or don’t remember

Q15 During the pandemic, did you have to delay or cancel any medical services that you needed unrelated to COVID-19?

1 Yes

2 No

3 Don’t know or don’t remember

Q16 Would you follow a vaccine requirement (mandate) in the future from any of the following? Select all that apply:

1 Government

2 Employer

3 School or university

4 None of the above

5 Don’t know

Q17 Would you follow a face mask requirement (mandate) in the future from any of the following? Select all that apply:

1 Government

2 Employer

3 School or university

4 None of the above

5 Don’t know

Q18 To your knowledge, do you have or have you had COVID-19?

1 Yes

2 No

3 Don’t know or don’t remember

Q19 "Long-COVID has been defined as 'symptoms that can last for weeks or months after recovery from acute illness'. To your knowledge, do you have or have you had Long COVID? "

1 Yes

2 No

3 Don’t know or don’t remember

Q20 Did you take any of the following medicines to treat COVID-19? Select all that apply:

1 Paxlovid

2 Molnupiravir (Lagevrio)

3 Monoclonal antibodies (Olumiant/Baricitinib)

4 Ivermectin

5 Traditional medicine, herbal extracts and treatments

6 Don’t know or don’t remember

7 No, I did not take medicines for COVID-19

Q21 Have you lost a family member or close friend to COVID-19 disease? Select all that apply:

1 Yes, within the past year

2 Yes, more than a year ago

3 No

Q22 I was satisfied with the communication efforts on COVID-19 vaccines made by health authorities in my country.

1 Strongly agree

2 Somewhat agree

3 Unsure/no opinion/don’t remember

4 Somewhat disagree

5 Strongly disagree

Q23 It is easy for me to know the difference between accurate and false information about the COVID-19 vaccine on the Internet.

1 Strongly agree

2 Somewhat agree

3 Unsure/no opinion/don’t remember

4 Somewhat disagree

5 Strongly disagree

Q24 I continue to pay attention to information on COVID-19 vaccines.

1 Strongly agree

2 Somewhat agree

3 Unsure/no opinion/don’t remember

4 Somewhat disagree

5 Strongly disagree

Q25 I continue to pay attention to information on vaccines in general.

1 Strongly agree

2 Somewhat agree

3 Unsure/no opinion

4 Somewhat disagree

5 Strongly disagree

Q26 The COVID-19 pandemic has made me more willing to get vaccinated against other diseases (e.g., flu, measles, viral hepatitis B).

1 Strongly agree

2 Somewhat agree

3 Unsure/no opinion

4 Somewhat disagree

5 Strongly disagree

Q27 My experience with the COVID-19 vaccine has affected my trust in other vaccines.

1 Increased trust

2 No effect

3 Decreased trust

4 Don’t know

Q28 Broadcast media coverage on COVID-19 vaccines has affected my trust in other vaccines.

1 Increased trust

2 No effect

3 Decreased trust

4 Don’t know

Q29 Social media coverage on COVID-19 vaccines has affected my trust in other vaccines.

1 Increased trust

2 No effect

3 Decreased trust

4 Don’t know

Q30 If an mRNA vaccine is approved for a disease for which you are at risk, would you take it?

1 Definitely no

2 Unsure, but leaning towards no

3 Unsure, but leaning towards yes

4 Definitely yes

Q31 If the World Health Organization (WHO) announced a new pandemic threat, would you trust this information?

1 Yes

2 No

3 Don’t know

Q32 If the World Health Organization (WHO) announced a new pandemic threat and advised getting vaccinated, would you?

1 Yes

2 No

3 Don’t know

Q33 "On a scale of 1 (trust completely) to 10 (do not trust at all) how much do you trust the following sources of information about COVID-19 vaccines: My family and friends"

Q34 My employer

Q35 My doctor or nurse

Q36 My government

Q37 Religious leaders

Q38 News media (e.g., television, Internet, radio, newspapers)

Q39 Social media (e.g., Facebook, Twitter [X], Instagram, WhatsApp, LinkedIn, TikTok)

Q40 World Health Organization (WHO)

Q41 Centers for Disease Control and Prevention (CDC), USA

Q42 European Centre for Disease Prevention and Control (ECDC)

Q43 The public health authorities in my country

Q44 How confident are you that we will manage the next health crisis better than the COVID-19 pandemic?

1 Not at all confident

2 Somewhat confident

3 Very confident

4 Don’t know

Q45 From what you have read or heard, do you think climate change/global warming is a real threat or not a real threat?

1 Real threat

2 Not a real threat

Q46 What is your age?

Q47 What is your gender?

1 Male

2 Female

3 Prefer not to say

Q48 Education (country specific)

1 Incomplete Secondary Education (Below GCSE / O Level)

2 Secondary Education Completed (GCSE / O Level / CSE or equivalent)

3 Secondary Education Completed (A Level or equivalent)

4 Vocational or Technical Qualifications Completed (e.g. HND, NVQ)

5 University Education Completed (First Degree, e.g. BA, BSc)

6 Postgraduate Education Completed (e.g. Masters)

7 Doctorate, Post-doctorate or equivalent (Higher Degree)

Q49 Median Income Level (country specific)

1 Yes, my monthly income is more than 3,173 British pounds.

2 Yes, my monthly income is less than 3,173 British pounds.

3 No, I do not have an income.

4 Refused/ Did not answer

Q50 Regions (country specific)

1 Scotland

2 Northern Ireland

3 Wales

4 South East

5 London

6 North West

7 East of England

8 West Midlands

9 South West

10 Yorkshire and the Humber

11 East Midlands

12 North East

United States

COVID-VAC: A global survey of COVID-19 vaccine perceptions

The purpose of the survey is to measure resident's reactions to COVID-19 response efforts. The risks in this routine survey are considered minimal. You may feel uncomfortable considering the implications of COVID-19 and participation in this research study is completely voluntary and you may discontinue participation at any time. Your responses will remain anonymous and confidential. The survey should take less than 5 minutes to complete. By answering the questions, you are indicating that you have read the description of the study, are over the age of 18, and that you agree to the terms as described.

If you have questions about your rights as a research participant, you may contact the Emerson College Institutional Review Board (IRB), which is concerned with the protection of volunteers in research projects. You may reach the lead investigator via email at emersonpolling@emerson.edu or the Chair of the IRB by e-mailing human_subjects@emerson.edu.

Q1 "First, please indicate your level of agreement with this statement: COVID-19 remains a dangerous health threat."

1 Strongly agree

2 Somewhat agree

3 Unsure/no opinion

4 Somewhat disagree

5 Strongly disagree

Q2 "Now, here are some questions related to COVID-19 vaccines. COVID-19 can be prevented by vaccination."

1 Strongly agree

2 Somewhat agree

3 Unsure/no opinion

4 Somewhat disagree

5 Strongly disagree

Q3 The risks of COVID-19 disease are greater than the risks of the vaccine.

1 Strongly agree

2 Somewhat agree

3 Unsure/no opinion

4 Somewhat disagree

5 Strongly disagree

Q4 The COVID-19 vaccines available to me are safe.

1 Strongly agree

2 Somewhat agree

3 Unsure/no opinion

4 Somewhat disagree

5 Strongly disagree

Q5 I trust the science behind the COVID-19 vaccines available to me.

1 Strongly agree

2 Somewhat agree

3 Unsure/no opinion

4 Somewhat disagree

5 Strongly disagree

Q6 The COVID-19 vaccines available to me are effective in protecting against severe COVID-19.

1 Strongly agree

2 Somewhat agree

3 Unsure/no opinion

4 Somewhat disagree

5 Strongly disagree

Q7 Have you received at least one dose of a COVID-19 vaccine?

1 Yes, I received one dose

2 Yes, I received two or more doses

3 No

Q8 Which of the following COVID-19 vaccines did you receive? Select all that apply:

1 Moderna (Spikevax)

2 Pfizer (Comirnaty)

3 Johnson&Johnson (Janssen or J&J)

4 Sinovac

5 Astra-Zeneca (Oxford, Vaxzevria)

6 COVAXIN

7 Other (please specify)

8 Don’t know

Q9 I will take the recommended COVID-19 booster.

1 Strongly agree

2 Somewhat agree

3 Unsure/no opinion

4 Somewhat disagree

5 Strongly disagree

Q10 I believe the COVID-19 pandemic is over.

1 Strongly agree

2 Somewhat agree

3 Unsure/no opinion

4 Somewhat disagree

5 Strongly disagree

Q11 How much did you trust your government's management of the COVID-19 pandemic in your country?

1 Did not trust

2 Somewhat did not trust

3 Unsure

4 Somewhat trust

5 Trust

6 Don't know or don't remember

Q12 Did the development of the COVID-19 vaccines affect your trust in the pharmaceutical industry?

1 Increased trust

2 No effect

3 Decreased trust

4 Don't know or don't remember

Q13 Did the development of COVID-19 vaccines affect your trust in science generally?

1 Increased trust

2 No effect

3 Decreased trust

4 Don't know or don't remember

Q14 How much do you trust the health authorities that recommended you get a COVID-19 vaccine?

1 Not at all

2 A little

3 Moderately

4 Very much

5 Don’t know or don’t remember

Q15 During the pandemic, did you have to delay or cancel any medical services that you needed unrelated to COVID-19?

1 Yes

2 No

3 Don’t know or don’t remember

Q16 Would you follow a vaccine requirement (mandate) in the future from any of the following? Select all that apply:

1 Government

2 Employer

3 School or university

4 None of the above

5 Don’t know

Q17 Would you follow a face mask requirement (mandate) in the future from any of the following? Select all that apply:

1 Government

2 Employer

3 School or university

4 None of the above

5 Don’t know

Q18 To your knowledge, do you have or have you had COVID-19?

1 Yes

2 No

3 Don’t know or don’t remember

Q19 "Long-COVID has been defined as 'symptoms that can last for weeks or months after recovery from acute illness'. To your knowledge, do you have or have you had Long COVID? "

1 Yes

2 No

3 Don’t know or don’t remember

Q20 Did you take any of the following medicines to treat COVID-19? Select all that apply:

1 Paxlovid

2 Molnupiravir (Lagevrio)

3 Monoclonal antibodies (Olumiant/Baricitinib)

4 Ivermectin

5 Traditional medicine, herbal extracts and treatments

6 Don’t know or don’t remember

7 No, I did not take medicines for COVID-19

Q21 Have you lost a family member or close friend to COVID-19 disease? Select all that apply:

1 Yes, within the past year

2 Yes, more than a year ago

3 No

Q22 I was satisfied with the communication efforts on COVID-19 vaccines made by health authorities in my country.

1 Strongly agree

2 Somewhat agree

3 Unsure/no opinion/don’t remember

4 Somewhat disagree

5 Strongly disagree

Q23 It is easy for me to know the difference between accurate and false information about the COVID-19 vaccine on the Internet.

1 Strongly agree

2 Somewhat agree

3 Unsure/no opinion/don’t remember

4 Somewhat disagree

5 Strongly disagree

Q24 I continue to pay attention to information on COVID-19 vaccines.

1 Strongly agree

2 Somewhat agree

3 Unsure/no opinion/don’t remember

4 Somewhat disagree

5 Strongly disagree

Q25 I continue to pay attention to information on vaccines in general.

1 Strongly agree

2 Somewhat agree

3 Unsure/no opinion

4 Somewhat disagree

5 Strongly disagree

Q26 The COVID-19 pandemic has made me more willing to get vaccinated against other diseases (e.g., flu, measles, viral hepatitis B).

1 Strongly agree

2 Somewhat agree

3 Unsure/no opinion

4 Somewhat disagree

5 Strongly disagree

Q27 My experience with the COVID-19 vaccine has affected my trust in other vaccines.

1 Increased trust

2 No effect

3 Decreased trust

4 Don’t know

Q28 Broadcast media coverage on COVID-19 vaccines has affected my trust in other vaccines.

1 Increased trust

2 No effect

3 Decreased trust

4 Don’t know

Q29 Social media coverage on COVID-19 vaccines has affected my trust in other vaccines.

1 Increased trust

2 No effect

3 Decreased trust

4 Don’t know

Q30 If an mRNA vaccine is approved for a disease for which you are at risk, would you take it?

1 Definitely no

2 Unsure, but leaning towards no

3 Unsure, but leaning towards yes

4 Definitely yes

Q31 If the World Health Organization (WHO) announced a new pandemic threat, would you trust this information?

1 Yes

2 No

3 Don’t know

Q32 If the World Health Organization (WHO) announced a new pandemic threat and advised getting vaccinated, would you?

1 Yes

2 No

3 Don’t know

Q33 "On a scale of 1 (trust completely) to 10 (do not trust at all) how much do you trust the following sources of information about COVID-19 vaccines: My family and friends"

Q34 My employer

Q35 My doctor or nurse

Q36 My government

Q37 Religious leaders

Q38 News media (e.g., television, Internet, radio, newspapers)

Q39 Social media (e.g., Facebook, Twitter [X], Instagram, WhatsApp, LinkedIn, TikTok)

Q40 World Health Organization (WHO)

Q41 Centers for Disease Control and Prevention (CDC), USA

Q42 European Centre for Disease Prevention and Control (ECDC)

Q43 The public health authorities in my country

Q44 How confident are you that we will manage the next health crisis better than the COVID-19 pandemic?

1 Not at all confident

2 Somewhat confident

3 Very confident

4 Don’t know

Q45 From what you have read or heard, do you think climate change/global warming is a real threat or not a real threat?

1 Real threat

2 Not a real threat

Q46 What is your age?

Q47 What is your gender?

1 Male

2 Female

3 Prefer not to say

Q48 Education (country specific)

1 Incomplete Secondary Education (Below GCSE / O Level)

2 Secondary Education Completed (GCSE / O Level / CSE or equivalent)

3 Secondary Education Completed (A Level or equivalent)

4 Vocational or Technical Qualifications Completed (e.g. HND, NVQ)

5 University Education Completed (First Degree, e.g. BA, BSc)

6 Postgraduate Education Completed (e.g. Masters)

7 Doctorate, Post-doctorate or equivalent (Higher Degree)

Q49 Median Income Level (country specific)

1 Yes, my monthly income is more than 3,173 British pounds.

2 Yes, my monthly income is less than 3,173 British pounds.

3 No, I do not have an income.

4 Refused/ Did not answer

Q50 Regions (country specific)

1 Northeast

2 South

3 Midwest

4 West

Q51 For statistical purposes only, can you please tell me your ethnicity?

1 Hispanic or Latino of any race

2 White or Caucasian

3 Black or African American

4 Asian

5 Other or multiple races
